# Single Nucleotide Polymorphisms with *Cis*-Regulatory Effects on Long Non-Coding Transcripts in Human Primary Monocytes

**DOI:** 10.1371/journal.pone.0102612

**Published:** 2014-07-15

**Authors:** Jonas Carlsson Almlöf, Per Lundmark, Anders Lundmark, Bing Ge, Tomi Pastinen, Cardiogenics Consortium, Alison H. Goodall, François Cambien, Panos Deloukas, Willem H. Ouwehand, Ann-Christine Syvänen

**Affiliations:** 1 Department of Medical Sciences, Molecular Medicine and Science for Life Laboratory, Uppsala University, Uppsala, Sweden; 2 Department of Human Genetics, McGill University, Montréal, Canada; 3 Department of Cardiovascular Science, University of Leicester, Leicester, United Kingdom; 4 Leicester NIHR Biomedical Research Unit in Cardiovascular Disease, Glenfield Hospital, Leicester, United Kingdom; 5 INSERM UMRS 937, Pierre and Marie Curie University and Medical School, Paris, France; 6 William Harvey Research Institute, Barts and The London School of Medicine and Dentistry, Queen Mary University of London, Heart Centre, Charterhouse Square London, United Kingdom; 7 Wellcome Trust Sanger Institute, Hinxton, Cambridge, United Kingdom; 8 Princess Al-Jawhara Al-Brahim Centre of Excellence in Research of Hereditary Disorders (PACER-HD), King Abdulaziz University, Jeddah, Saudi Arabia; 9 National Health Service Blood and Transplant, Cambridge Centre, Long Road, Cambridge, United Kingdom; Medical University Hamburg, University Heart Center, Germany

## Abstract

We applied genome-wide allele-specific expression analysis of monocytes from 188 samples. Monocytes were purified from white blood cells of healthy blood donors to detect *cis*-acting genetic variation that regulates the expression of long non-coding RNAs. We analysed 8929 regions harboring genes for potential long non-coding RNA that were retrieved from data from the ENCODE project. Of these regions, 60% were annotated as intergenic, which implies that they do not overlap with protein-coding genes. Focusing on the intergenic regions, and using stringent analysis of the allele-specific expression data, we detected robust *cis-*regulatory SNPs in 258 out of 489 informative intergenic regions included in the analysis. The *cis*-regulatory SNPs that were significantly associated with allele-specific expression of long non-coding RNAs were enriched to enhancer regions marked for active or bivalent, poised chromatin by histone modifications. Out of the lncRNA regions regulated by *cis-*acting regulatory SNPs, 20% (n = 52) were co-regulated with the closest protein coding gene. We compared the identified *cis*-regulatory SNPs with those in the catalog of SNPs identified by genome-wide association studies of human diseases and traits. This comparison identified 32 SNPs in loci from genome-wide association studies that displayed a strong association signal with allele-specific expression of non-coding RNAs in monocytes, with p-values ranging from 6.7×10^−7^ to 9.5×10^−89^. The identified *cis*-regulatory SNPs are associated with diseases of the immune system, like multiple sclerosis and rheumatoid arthritis.

## Introduction

Determination of allele-specific gene expression (ASE) levels by quantitative genotyping of heterozygous SNPs on the RNA level [1], with genome-wide panels of SNPs [2], [3] can be used as a guide for identifying *cis*-acting genetic variants that regulate gene expression. ASE analysis is more powerful for detecting *cis*-regulated gene expression than the total expression levels of genes determined by regular eQTL analysis [4]. The power and precision of ASE analysis to detect *cis*-regulatory SNPs (*cis*-rSNPs) stems from the fact that the differential expression of the two alleles of a SNP are measured in the same environment and have been exposed to the same environmental conditions in the cells from which the RNA was extracted [4]. Thus *trans-*acting and environmental factors that may affect gene expression are controlled for in ASE analysis. To adjust for possible methodological differences in the efficiency of the genotyping assay to measure the two alleles of a SNP, such as the sequence context of a SNP or copy number variations, the relative amounts of the alleles measured in DNA, is used as a reference for quantification of the allelic expression. Genome-wide ASE analysis by SNP genotyping has been applied to map *cis*-regulation of protein coding genes associated with human diseases and traits in lymphoblastoid cell lines [2], [5], osteoblasts [6], fibroblasts [5], [7], T-cells [8], and monocytes [5].

Long non-coding RNAs (lncRNAs) are involved in gene regulation and other cellular processes. LncRNAs such as XIST and TSIX, which are involved in X chromosome inactivation [9], [10], are well known. Other well known examples of lncRNAs are AIR that suppresses gene expression via hypermethylation and HOTAIR that interacts with the Polycomb Repressive Complex 2 to silence the HOXD locus [11]. New functions for lncRNAs are continuously being discovered. LncRNAs can affect gene expression in many ways, as scaffolds or guides for chromatin modifications, as decoys for reducing the amount of transcription factors interacting with chromatin, as signaling molecules reflecting active transcription factor complexes, or as reservoirs for microRNAs [12]. The mechanism for regulation of gene expression by lncRNAs usually involves formation of RNA-protein complexes that influence the gene expression. Recent large studies [13], [14], [15], [16], [17], [18] have found that lncRNAs display positive correlations with expression of protein-coding genes in *cis*, and especially with genes that overlap with the antisense strand of a lncRNA. By investigating the effect of regulatory SNPs on the expression of lncRNAs using traditional microarray-based expressed quantiative trait locus (eQTL) analysis of peripheral blood cells a recent study identified 112 *cis*-regulated lncRNAs [19].

The aim of our study was to apply ASE-analysis to identify *cis*-acting SNPs that regulate the expression of lncRNAs and to highlight previously unknown roles for lncRNAs in human complex diseases. We reasoned that ASE-analysis that is highly sensitive for detecting *cis*-rSNPs would be a powerful tool for studying *cis*-regulation of lncRNAs that are expressed at lower levels than protein-coding genes. To our knowledge ASE-analysis has not been previously used for studies of lncRNAs.

We analysed RNA extracted from human monocytes purified from white blood cells of 188 healthy blood donors using a genome-wide panel of SNPs. Monocytes were selected for analysis because they are a relevant cell type for multiple diseases. Our analysis included 8929 genomic regions that harbor genes for potential long non-coding RNA that were retrieved from the GENCODE database [18]. Of these gene regions, 60% were annotated as intergenic, and thus they do not overlap with protein-coding genes. Using stringent criteria for the identification of SNPs that regulate the expression of lncRNAs we identified 8267 *cis*-acting regulatory SNPs out of which 3910 are located in intergenic regions and 571 of these are located in enhancer regions marked as active or poised by histone modifications in monocytes. We also compared the indentified SNPs that regulate expression of lncRNAs with risk SNPs for complex diseases and traits previously identified in genome-wide association studies (GWAS). In this way we were able to obtain new functional clues for these disease associated GWAS loci. Additionally, we analysed co-expression between lncRNA genes and nearby protein coding genes, and found that 20% of the intergenic lncRNAs with a *cis*-rSNP were co-regulated with a protein-coding gene.

## Materials and Methods

### Samples

Circulating monocytes were collected from healthy adult blood donors of European origin (n = 188) recruited from the United Kingdom National Blood Service Centre in Cambridge, UK as part of the Cardiogenics Transcriptomic Study [20]. Volunteer donors with a self-reported recent or acute illness were excluded. Donors with measured full blood cell count and C-reactive plasma protein levels outside the normal ranges were also excluded from the study.

DNA was extracted from peripheral blood leukocytes using the guanidine hydrochloride - chloroform method. CD14+ magnetic microbeads (autoMACS Pro, Miltenyi Biotec, Bergisch Gladbach, Germany) were used to isolate monocytes from whole blood. RNA was extracted from cell pellets of freshly isolated monocytes by homogenization with Trizol-reagent (Invitrogen, Paisley, UK), chloroform-ethanol extraction and purification using Qiagen RNAeasy columns and reagents, followed by on-column DNase treatment. cDNA was synthesized using reagents from the Illumina TotalPrep RNA Amplification Kit, except that the poly-dT primers were substituted by random decamers (Applied Biosystems, Carlsbad, California, US).

### Ethics statement

All participants in the study provided their written consent after a personal meeting with a study nurse. Written consent from each participant was recorded by name, date and signature on a standardized consent form. Both the consent form and the study has been reviewed and approved by the Cambridgeshire 1 Research Ethics Committee. The Research Ethics Committee is independent from the research institute and part of the UK Health Research Authority.

### ENCODE lncRNA Regions

A total of 8929 genomic regions containing lncRNAs were retrieved from the GENCODE version 7 database [18]. From regions containing several transcripts, only the longest transcript was included to avoid analyzing completely overlapping regions. Of the lncRNA regions, 5346 were annotated as intergenic, which implies that that there is no overlap between the lncRNA and any known protein coding gene. The remaining 3583 regions overlap with either exons, introns or encompass an entire gene on the sense or anti- sense strand.

### Allele-specific expression analysis

RNA (cDNA) and genomic DNA (gDNA) from the monocyte samples were genotyped using the Infinium assay and Human 1.2 M Duo custom BeadChips (Illumina, San Diego, California, USA) as described previously [4]. The genotype data from cDNA is used as a quantitative measure for gene expression in the calculation of the ASE-levels. The genotypes called in gDNA are used for three different purposes i.e. (i) to phase the SNP alleles on each chromosome to facilitate ASE-calling using multiple SNPs per transcript, (ii) to correct the ASE-levels in cDNA for possible allelic bias in gDNA due to sequence context of the SNPs, and (iii) to test for associations between potential *cis*-rSNPs and the ASE-levels of lncRNAS in a genomic region. Genotypes were called in gDNA using Genome Studio version 2009.2 (Illumina) with a call rate of 99% as the threshold for genotype calls for SNPs and 98% for samples. SNPs were further filtered on deviations from Hardy-Weinberg equilibrium with a p-value cutoff of 10^−6^ (Chi-squared test). One sample with higher than 40% call rate in cDNA were removed due to possible DNA contamination. The raw two-colour fluorescence signals from the Infinium assay were normalized to remove dependency of the allele fractions on the signal intensities of the fluorophores using a quadratic function with medians of bins for the two fluorescence colors. From the regression, the intensities were predicted based on the log10 values of the raw signal, which were used to adjust the allele fraction, see Methods S1 for further details and equations. ASE levels were calculated for each heterozygote SNP as the difference in normalized allele fractions between cDNA and gDNA: [Allele1_cDNA_/(Allele1_cDNA_+Allele2_cDNA_)] – [Allele1_gDNA_/(Allele1_gDNA_+Allele2_gDNA_)]. Here, gDNA, where the two SNP alleles are present in 1∶1 ratio serves as a quantification standard for the relative expression levels of the two alleles in RNA.

A gene region without ASE will obtain an ASE-level close to 0, while a gene region with ASE, where the gDNA fraction is 0.5, will have an absolute ASE-level 0<X< = 0.5. The software IMPUTE 2 [21] (v2.1.0) was used to impute missing genotypes and to phase the genotypes across lncRNA gene regions in each individual sample. As a reference panel prefiltered haplotypes from HapMap3 release #2 [22] and 1000 Genomes pilot1 [23] available at the IMUPUTE 2 website were used. The ASE levels were assigned in each individual sample as the average ASE level for all heterozygous SNPs within a genomic region (ASE window), corresponding to the region of an annotated lncRNA. Windows of lncRNAs with less than three informative heterozygous SNPs were excluded. The association between SNPs and the ASE levels of lncRNAs was assessed by linear regression of the ASE levels in the groups of samples with heterozygous SNPs and homozygous SNPs [4]. In summary, homozygous SNPs (AA and BB) are in one group with an expected ASE-level close to zero, and heterozygous SNPs are either AB or BA with a stochastically determined direction during phasing. In the case of significant ASE the AB and BA group will on average obtain different signs of the ASE-level. SNPs with significant associations with allele specific expression are denoted as *cis*-rSNPs (Figure S1).

All analyses described above were performed and data is presented using the NCBI36/hg18 assembly as reference genome.

### Refseq protein coding regions

Protein coding regions extracted from the Refseq database were analysed to detect SNPs with association signals that overlap with those from lncRNAs. The total number of transcripts in Refseq (downloaded 12^th^ of January 2013) was 42797. To retain only protein coding genes, all genes annotated as RNA genes in the GeneCards database [24] were removed, leaving 38387 transcripts. To avoid completely overlapping transcripts, 19654 transcripts that are a subsequence of another transcript were removed. As for lncRNAs, only regions that contained at least three informative SNPs were included in the analysis, leaving 10345 protein-coding regions for further analysis.

### Determination of total gene expression levels using the genotype data

The sum of the raw fluorescence signal intensities from both alleles of a SNP in cDNA was used as a measure of total gene expression in the position of the SNP. The average sum of the SNP signals across each lncRNA window and across all samples represents the total expression value for each lncRNA window. A stringent average signal threshold of 1000 fluorescence units for all 188 samples was set for the summed signal intensities, see Figure S2.

### Enhancer regions

Monocyte chromatin immunoprecipitation sequencing (ChIP-seq) data was retrieved from the Blueprint project [25]. We analysed the four samples available in the Blueprint data using two chromatin signatures for each sample; H3K27ac, corresponding to an active enhancer [26], [27] and H3K4me1, corresponding to a poised enhancer [28], [29]. The peaks supplied in the Blueprint data had been called by MACS2 using the standard parameters for each signature. For a ChIP-seq peak to be included in our analysis, we required a p-value of 10^−5^ in at least two out of four overlapping peaks. To be counted as an overlapping peak the nucleotide overlap was required to be over 50%. This resulted in 22091 peaks for H3K27ac and 32692 peaks for H3K4me1.

### Co-expression of lncRNA and closest protein coding gene

We investigated to what extent expression of lncRNA genes with a *cis*-rSNP were correlated with gene expression of the closest protein coding gene. For this purpose we divided the samples into three groups based on three genotype combinations of the most significantly associated *cis*-rSNP (AA, AB/BA, BB). For each expressed gene region we calculated the average signal intensity for both alleles and used this value as a measure of gene expression levels. Next, we performed linear regression analysis with the three genotype groups as x-values and the average total expression levels as y-values in order to obtain a p-value for the co-expression.

## Results

### Allele-specific expression of lncRNA regions

**Figure 1:**
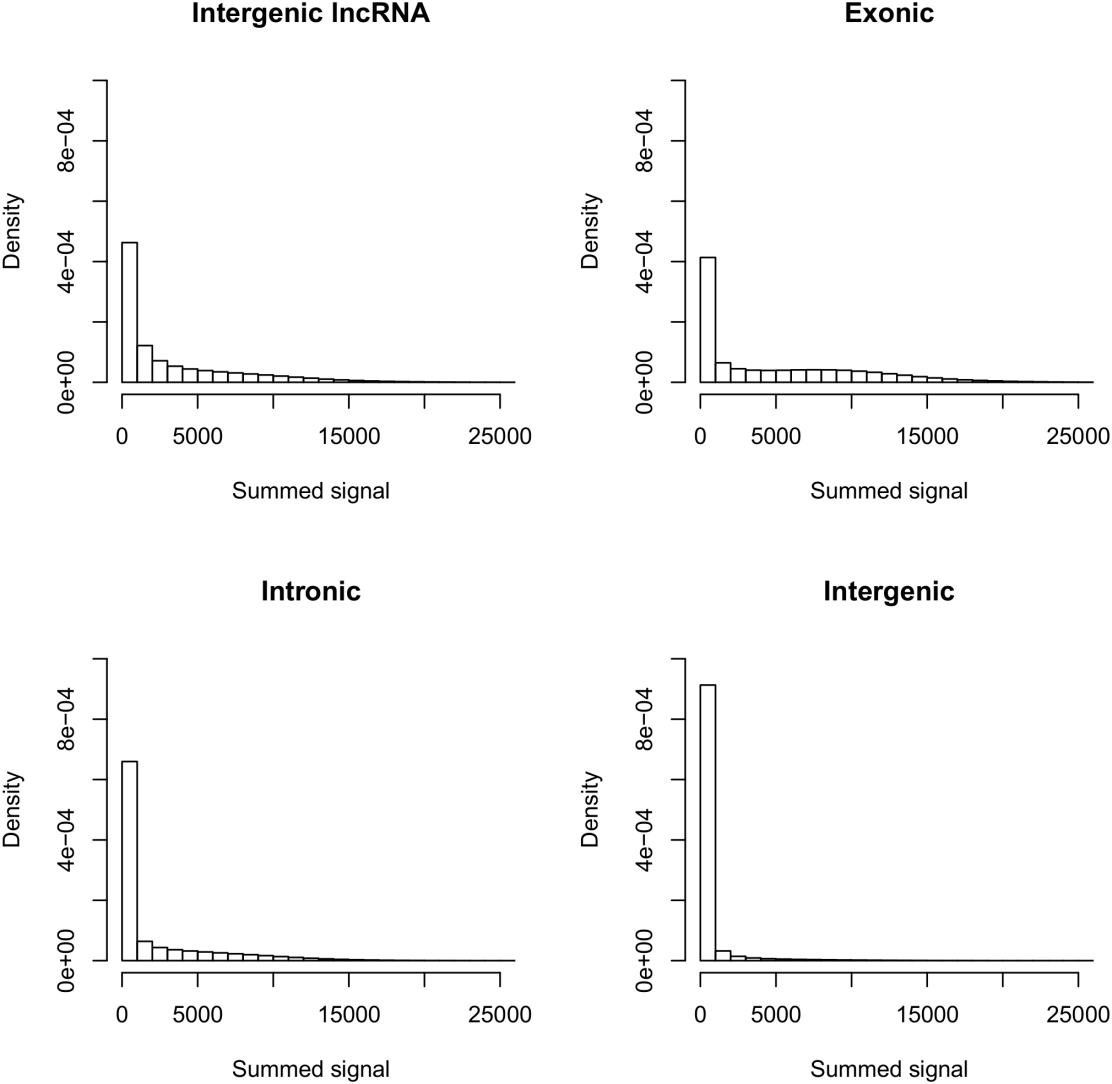
Comparison of total expression levels of regions annotated to intergenic lncRNAs, exons, introns, and intergenic regions. The horizontal axes in the panels show bins of fluorescence signals from the genotyping data, summed for both alleles to give a measure for total expression. The average expression levels of annotated transcripts were 4900 fluorescence units in exons, 2100 in introns, 590 in intergenic regions, compared to 3300 in the intergenic lncRNA regions that were used in the ASE analysis. The vertical axes show the number of observations in each bin.

We explored *cis*-regulation of 8929 genomic regions harboring lncRNA from the ENCODE project using allele-specific expression (ASE) analysis of 188 RNA samples from human primary monocytes. To determine ASE, we genotyped RNA (cDNA) and genomic DNA from the monocytes using a genome-wide panel of 1.2 million SNP markers [4]. To be informative for the detection of ASE, a SNP has to be heterozygous in DNA and expressed at a detectable level in RNA. Out of the 8929 lncRNA regions, 60% were annotated as intergenic. 1298 regions contain at least three informative SNPs and out of these 1122 regions (489 intergenic regions) showed a signal intensity above background and were considered to be expressed in the monocytes and were thus included in the ASE analysis. Of the individual SNPs in the lncRNA regions, two thirds have an intensity level above 1000 in at least 90% of the samples. We found that the mean expression levels of the lncRNAs were 1.5-fold lower than the expression levels of exons (Figure 1), while the lncRNAs were expressed at a 1.5-fold and 5.5-fold higher level than intronic and inter protein coding gene regions, respectively.

The definitions of exon and intron boundaries were taken from Refseq. As protein coding genes are on average expressed at higher levels than lncRNAs, the ASE signal for lncRNA that overlap with protein coding genes is hard to distinguish from that of a protein-coding gene. Because of this, we focus on the intergenic lncRNA regions where the ASE data is most reliable.

**Figure 2:**
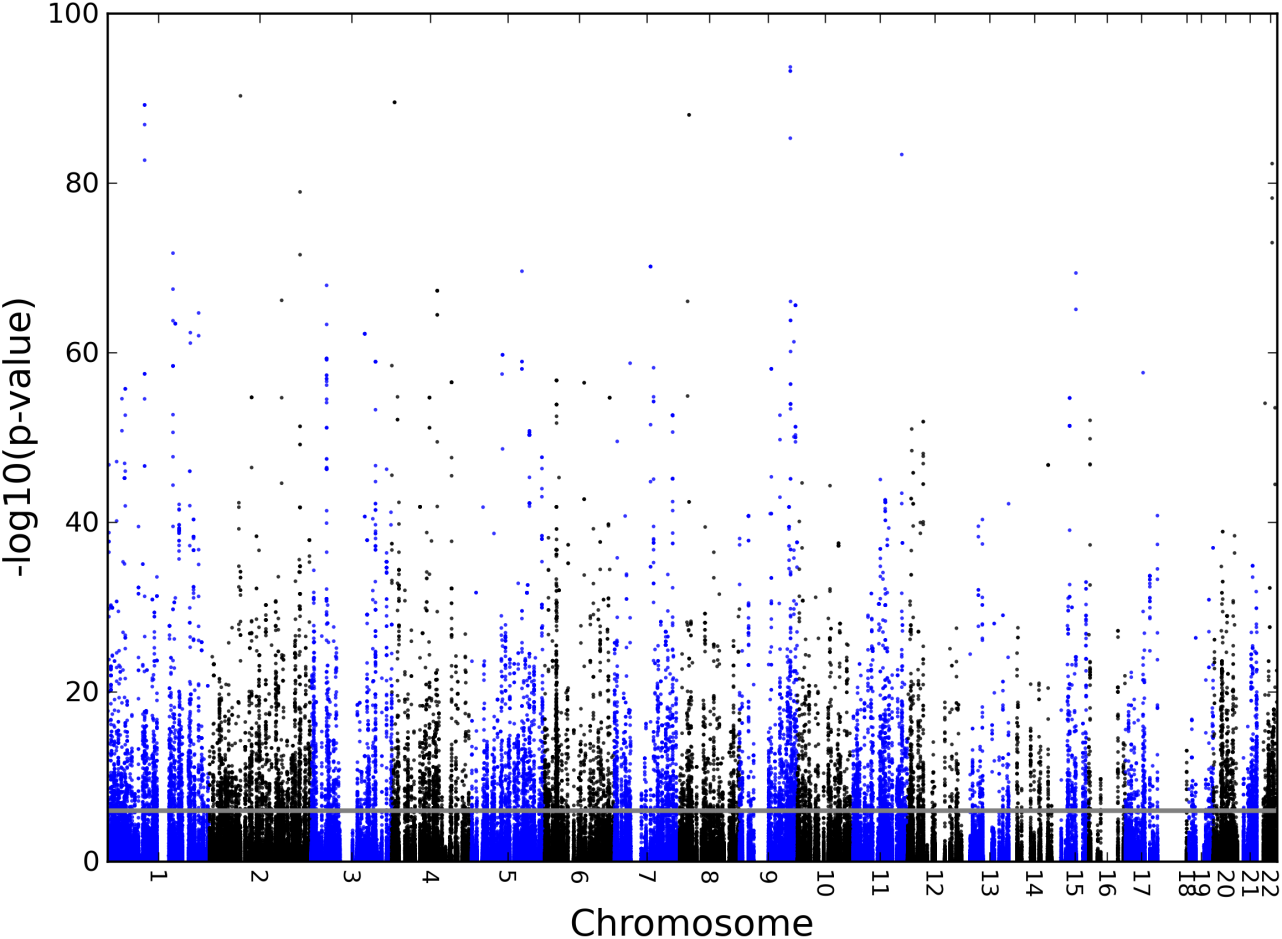
Manhattan plot. Manhattan plot with the p-values from ASE association tests between SNPs and lncRNAs on the vertical axis and the genomic lncRNA regions analysed in the study on the horizontal axis. The p-value cut-off of 10^−6^ is shown as a grey line.

For the ASE analysis, the genotypes of 194530 SNPs located within 250 kb upstream and downstream of the lncRNA regions were tested for their association with the ASE levels of the corresponding expressed lncRNAs, using linear regression, see Manhattan plot in Figure 2. A p-value cut-off of p<10^−6^, based on the number of tests performed and adjusted for SNPs in high LD (>0.9), was used to correct the association signals for multiple testing. In addition a threshold of 0.05 for the slope of the regression line was applied to exclude SNPs with low effects on ASE. This value corresponds to approximately a 20% expression difference between the two alleles and was set as a reasonable threshold for biological relevance. To avoid false positives with inflated p-values, we only retained associations that were based on at least four data points for each allele combination for the calculation of the p-value [4] (see example regression plots in Figure S3). Using these criteria, we detected 258 intergenic (53%) lncRNAs with at least one associated *cis*-rSNP (Table S1). The associated intergenic lncRNA regions varied in size from 0.9 kb to 547 kb. A total of 8267 of the 194530 tested SNPs (4.2%) were associated with ASE of a lncRNA region (p-value<10^−6^) in the 188 analysed monocyte samples, and for the intergenic lncRNA regions 3910 out of 79628 tested SNPs (4.9%) were significantly associated *cis*-rSNPs.

To allow comparison between ASE of lncRNA genes and protein coding Refseq genes, we applied the same procedure for ASE analysis to the Refseq gene regions as for the lncRNAs. This analysis identified 8604 *cis*-rSNP out of 108881 tested SNPs (7.9%) in 471 out of 10345 regions (4.6%) that were associated with ASE of a Refseq gene.

### Associated SNPs in enhancer regions

We identified 571 *cis*-rSNPs located in enhancer regions marked as active or poised by histone modifications in monocytes. To determine if the *cis*-rSNPs for lncRNAs are enriched in enhancer regions, we calculated the fold enrichment of significantly associated *cis*-rSNPs defined by ASE analysis of intergenic lncRNA regions to histone marks for active enhancers (H3K27ac) and bivalent, poised enhancers (H3K4me1) in monocytes. The fold enrichment was determined by comparing the fraction of significantly associated SNPs in enhancer regions with the fraction of non-significant (p-value>0.5) SNPs in enhancer regions. Of the significantly associated SNPs, 2.1% were located in H3K27ac peaks compared to 1.2% for non-significantly associated SNPs, which corresponds to a 1.72-fold enrichment (p-value 9.0×10^−6^, Fisher’s exact test). For H3K4me1 we observed a 1.54-fold enrichment (p-value 3.1×10^−16^, Fisher’s exact test) of significantly associated SNPs, with 12.5% of the significant SNPs located in the enhancer peaks, while this number was 8.1% for non-significant SNPs. For protein coding genes the percentages and fold enrichment are similar to those for lncRNA for H3K27ac with 2.2% and 1.5% for significant and non-significant SNPs, respectively, and 1.43 fold enrichment (p-value 7.0×10^−6^, Fisher’s exact test) and less pronounced for H3K4me1 with 9.8% and 8.5% for significant and non-significant SNPs, respectively, and 1.15 fold enrichment (p-value 3.5×10^−4^, Fisher’s exact test). This result shows that enhancers have a regulatory *cis*-effect on expression of not only protein coding genes, but also lncRNAs.

### Co-expression of lncRNA and closest protein coding gene

Next we investigated whether the lncRNA genes and nearby protein coding genes are co-expressed and co-regulated by a *cis*-rSNP. In our data 52 (20%) of the identified intergenic lncRNA regions to which a *cis*-acting regulatory SNP was associated were also correlated with the expression level of a protein-coding gene at a false discovery rate (FDR) of 10%, suggesting co-regulation of the genes, see Table 1. We found that 21 of the *cis*-rSNPs are significantly associated both with ASE of a lncRNA and ASE of the closest protein coding gene, which implies that the expression of the lncRNA and the protein coding genes are regulated by the same *cis*-rSNP. For 19 *cis*-rSNPs the ASE signal is unique to a lncRNA, which could be an indication of involvement of the lncRNA in the regulation of the expression of an adjacent protein coding gene as has been suggested in previous studies [14], [16]. The remaining 12 *cis*-rSNPs had a distance >250 kb to the nearest protein coding gene and the ASE signal could therefore not be evaluated.

**Table 1:** Co-expressed lncRNA regions and protein-coding genes.

SNP^1^	lncRNAregion	Refseqgene	p-value forco-expression^2^	p-value forASE of lincRNA^3^	p-value forASE ofRefseq gene^4^
**Co-regulated lncRNA and protein-coding by the same ** ***cis*** **-rSNP**					
rs644234	9∶135120874–135140438	ABO	5.38E-26	2.69E-66	2.69E-66
rs1056787	4∶67965618–68016564	CENPC1	6.75E-06	9.89E-25	1.37E-57
rs7313235	12∶9980445–9987361	CLEC12A	1.92E-18	2.23E-37	5.00E-56
rs2271101	4∶178603127–178835274	AGA	1.81E-12	4.79E-23	4.68E-54
rs1112956	5∶127304017–127446691	SLC12A2	1.62E-10	2.56E-70	7.74E-49
rs10240848	7∶38347741–38384763	AMPH	3.45E-05	1.80E-59	2.24E-45
rs2071904	22∶48328333–48336125	C22orf34	1.92E-08	3.14E-23	4.02E-43
rs2254177	17∶45713937–45720215	TMEM92	1.02E-12	1.99E-16	2.02E-41
rs1044303	6∶131190239–131199966	SMLR1	1.46E-05	2.26E-30	2.26E-30
rs2949192	7∶1166536–1171429	ZFAND2A	1.24E-03	1.70E-26	2.60E-30
rs9355652	6∶158623283–158653378	TULP4	1.38E-02	2.12E-55	2.31E-28
rs8112960	19∶21562075–21568648	ZNF429	1.85E-24	4.41E-27	7.21E-24
rs7647643	3∶158363935–158369616	CCNL1	2.53E-06	2.91E-31	1.77E-22
rs7336525	13∶20775651–20820860	MIPEPP3	1.91E-11	3.63E-25	5.02E-21
rs12366	2∶75020893–75023305	POLE4	1.83E-04	5.44E-91	3.18E-19
rs672527	1∶180685904–180796316	RGSL1	1.40E-07	1.06E-18	1.06E-18
rs17802159	4∶114420–147779	ZNF876P	6.86E-06	3.48E-59	6.90E-15
rs10463951	5∶135493095–135498478	SMAD5	7.96E-03	6.44E-14	4.58E-14
rs178255	22∶19641380–19648967	AIFM3	1.04E-04	2.34E-20	5.08E-14
rs12711793	2∶114453616–114481349	ACTR3	2.34E-03	1.50E-25	5.58E-14
rs9605146	22∶15462934–15514699	TPTEP1	3.68E-16	5.12E-08	5.12E-08
**Regulation of lncRNA by ** ***cis*** **-rSNP, but not of protein-coding gene**					
rs246105	5∶108600720–108689969	PJA2	1.20E-03	5.12E-24	2.83E-06
rs3862666	11∶60579785–60591583	CD5	7.26E-03	4.08E-08	6.24E-06
rs948421	8∶61459701–61591893	RAB2A	8.47E-03	6.08E-30	9.84E-06
rs3737813	1∶178162399–178177554	TOR1AIP2	2.40E-03	3.38E-10	4.38E-05
rs850942	12∶13044652–13084880	KIAA1467	1.01E-02	9.33E-20	9.90E-05
rs269782	5∶139517088–139528554	CYSTM1	3.41E-03	2.11E-32	1.20E-04
rs12192704	6∶30874410–30906415	DDR1	6.61E-04	1.85E-37	1.55E-04
rs4978941	9∶112401576–112407320	SVEP1	3.13E-08	1.61E-14	3.30E-03
rs8041057	15∶38118804–38146783	SRP14	9.96E-04	1.60E-28	4.44E-03
rs6744457	2∶216119166–216286863	FN1	3.71E-06	1.85E-10	9.65E-03
rs10167593	2∶71083169–71145381	NAGK	1.53E-03	5.13E-43	3.61E-02
rs3809472	15∶43458687–43481812	SPATA5L1	1.24E-07	2.28E-55	4.00E-02
rs10982360	9∶116468537–116473850	C9orf91	9.63E-04	3.25E-24	1.74E-01
rs1887784	9∶116458405–116464475	C9orf91	6.83E-05	2.50E-22	2.66E-01
rs628383	3∶152071522–152094779	CLRN1	4.74E-34	2.20E-14	2.67E-01
rs2798686	1∶113355832–113417250	LRIG2	1.07E-02	4.45E-30	3.36E-01
rs12343516	9∶122645199–122654702	PHF19	2.96E-06	2.11E-94	6.18E-01
rs8112960	19∶21561436–21697351	ZNF100	8.66E-06	7.45E-09	8.47E-01
rs4916908	5∶87600489–87768258	TMEM161B	6.53E-03	1.30E-28	8.50E-01
**Unknown mode of regulation**					
rs1870832	6∶89065388–89187471	CNR1	7.91E-12	1.68E-28	NA
rs17739675	2∶64418705–64422285	LGALSL	5.01E-08	5.71E-17	NA
rs841603	12∶27149351–27205593	C12orf71	1.36E-03	8.20E-28	NA
rs1868841	8∶58568012–58666672	FAM110B	3.55E-05	3.06E-19	NA
rs1348478	5∶119609042–119697096	PRR16	1.69E-02	9.64E-14	NA
rs7046236	9∶70345772–70437996	TMEM252	4.83E-06	6.12E-24	NA
rs10772397	12∶11256393–11295570	PRB3	1.04E-28	1.04E-51	NA
rs9423393	10∶5266321–5295165	AKR1C4	2.19E-03	8.29E-41	NA
rs928736	21∶33352005–33359159	OLIG1	1.31E-10	1.00E-27	NA
rs7132674	12∶11443582–11530879	PRB2	3.28E-33	2.82E-11	NA
rs17739675	2∶64475858–64534436	LGALSL	5.01E-08	1.14E-10	NA
rs10956365	8∶128420701–128474058	POU5F1B	3.22E-11	1.23E-07	NA

1 All 52 significantly co-expressed lncRNA and protein coding genes with a lncRNA associated *cis*-rSNP are listed.

2 Multiple testing correction using FDR of 10%,

3 The p-value cut-off for significant ASE is 10^−6^,

4 ASE p-value for a Refseq gene is shown for all regions that are co-expressed with a lncRNA and have an overlapping ASE analysis window, NA otherwise.

### GWAS lead SNPs overlap with *cis*-rSNP for lncRNAs

**Figure 3:**
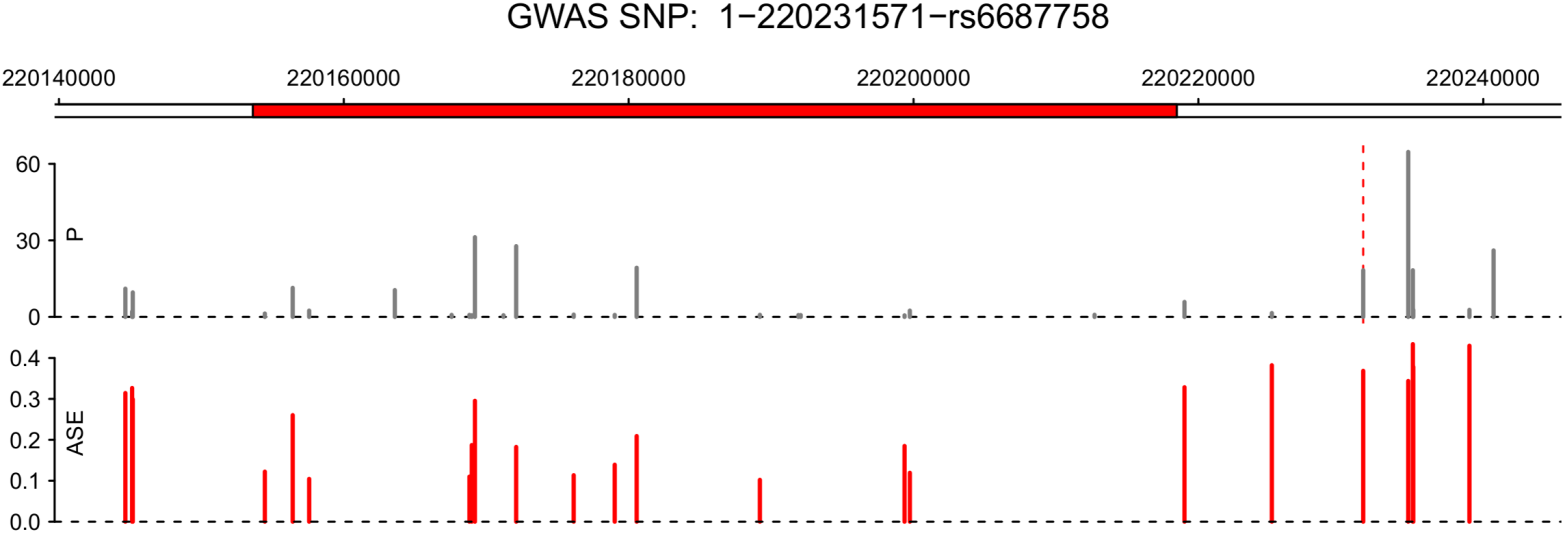
Illustration of a region with a SNP from genome wide association studies (GWAS) which is associated with ASE of lncRNAs. The tracks are from top to bottom in each panel: Horizontal red bars represent lncRNA transcript windows (with genomic coordinates) used for determination of ASE levels; grey lines show p-values for the association of GWAS SNPs with ASE levels in the transcript window; a grey line overlayed with a red dotted line indicates that a *cis*-rSNP overlaps with the reported SNP in the GWAS catalog; red vertical lines are median ASE-levels for each SNP.

Next we superimposed the *cis*-rSNP associated with ASE of intergenic lncRNAs in monocytes with SNPs identified in GWAS of human diseases and traits, listed in the National Human Genome Research Institute (NHGRI) GWAS Catalog [30]. The GWAS catalog (downloaded 12^th^ of January 2013) includes 9617 entries, 641 traits and 7797 unique SNPs. We identified SNPs associated with 32 loci high-lighted by GWAS that are also *cis*-rSNP for lncRNAs. The *cis*-rSNP are the same as the GWAS associated SNP for 25 of the loci, while seven *cis*-SNPs are linkage disequilibrium (LD) proxies to the lead GWAS SNPs, with five SNPs having LD = 1.0 and two having LD> = 0.88. Table 2 summarizes the data for overlapping GWAS SNPs and *cis*-rSNP associated with ASE of lncRNA regions as well as the phenotypes to which these SNPs were associated in GWAS. Figure 3 exemplifies a lncRNA region with association between a SNP reported in GWAS and allele-specific expression. For additional examples see Figure S4. The association signals highlight 26 unique traits, out of which 9 are related to the immune system, which is not unexpected given that monocytes function in the immune system. Notably, three of the *cis*-rSNP that were significantly associated with ASE of a lncRNA are located in an intron of a protein coding gene, illustrating a direct or indirect regulatory function of an intronic SNP. One locus is reported by GWAS to be associated with a non-coding gene (genomic region: 2:19067188–19369296), which we confirm here by a *cis*-rSNP for the same lncRNA gene. Furthermore, when searching for overlaps between *cis*-rSNP associated with lncRNA and GWAS SNPs that are located in active enhancer regions in monocytes, we detected three GWAS SNPs that are located in a ChIP-seq peak for H3K4me1, and seven additional SNPs that are in the 2.5 kb enhancer regions flanking the histone mark. For H3K27ac no overlap between the *cis*-rSNP and the actual ChIP-seq peak was found, but five SNPs were located in the 2.5 kb flanking region. The number of overlapping GWAS SNPs – *cis*-rSNPs that map to an enhancer region are enriched at the same level as *cis*-rSNPs in general. For these GWAS SNPs the effect on the enhancer could be the functional mechanism.

**Table 2:** SNPs associated with allele-specific expression of lncRNA windows with published trait- or disease-associations from genome-wide association studies.

	ASE				GWAS	
Genomicregion oflncRNA	Distance toGWASSNP (bp)^1^	In or closeto histonemark	ASEp-value	Slope^2^	SNP^3^	Trait^4^
1: 22224268–22229983	16228	H3K4m1^5^	2.41E-7	0.13	rs2501276	Immune responseto smallpoxvaccine (IL-6)
1: 22224268–22229983	128475		3.03E-7	0.07	rs16826658	Endometriosis
1: 41480834–41523120	177376		2.66E-12	0.09	rs6686842^6^	Height
1: 220153613–220218488	13083	H3K4m1	8.46E-17	0.22	rs6687758	Colorectalcancer
2: 19067188–19369296	68959		1.36E-7	0.06	rs1876040	Cognitive testperformance
2: 101944950–101970061	59999		1.32E-7	0.08	rs2310173	Ankylosingspondylitis
2: 166359613–166410847	68123		5.60E-9	0.06	rs6710518	Bone mineraldensity
2: 166359613–166410847	50321		1.82E-9	0.06	rs1346004^7^	Bone mineraldensity
3: 157947829–158017517	91268		3.72E-25	0.08	rs12638253	Multiplesclerosis(severity)
6: 28237538–28245351	184924	H3K4m1^5^H3K27ac^5^	2.25E-8	0.06	rs6903823^8^	Pulmonaryfunction
6: 30050868–30054162	7639	H3K4m1	5.90E-11	0.08	rs3893464	Graves’disease
6: 29802357–29824805	257099	H3K27ac^5^	6.18E-7	0.08	rs4313034^9^	Graves’disease
6: 30874410–30906415	204180		4.05E-12	0.11	rs4248154	Graves’disease
6: 58380311–58395738	21176		3.60E-21	0.07	rs9500256^10^	Eosinophilicesophagitis(pediatric)
7: 1166536–1171429	161007		3.40E-14	0.07	rs10256972	Longevity
7: 7261310–7283935	26354		4.86E-9	0.07	rs10259085	Multiplesclerosis(severity)
7: 7261310–7283935	0	H3K4m1^5^	3.41E-7	0.06	rs1299548	Amount of visceraladipose tissue adjustedfor body massindex (BMI)
8: 23138679–23144384	0	H3K4m1^5^H3K27ac^5^	9.45E-89	0.27	rs13278062	Age-relatedmacular degeneration
9: 122645199–122654702	25619	H3K4m1H3K27ac^5^	4.09E-16	0.13	rs1953126	Celiac disease andRheumatoid arthritis
9: 122645199–122654702	38017		1.02E-16	0.13	rs881375	Rheumatoidarthritis
9: 122645199–122654702	75358	H3K4m1^5^H3K27ac^5^	4.13E-17	0.13	rs3761847	Rheumatoidarthritis
9: 135120874–135140438	0		3.39E-50	0.43	rs687621	D-dimerlevels
9: 135120874–135140438	0		2.69E-66	0.43	rs657152	Liver enzymelevels
9: 135120874–135140438	0		2.69E-66	0.43	rs643434	Inflammatorybiomarkers
9: 135120874–135140438	0		5.66E-52	0.44	rs612169	Metabolictraits
9: 135120874–135140438	0		1.00E-50	0.44	rs505922	Proteinquantitativetrait loci
9: 135120874–135140438	0		3.80E-22	0.43	rs507666	SolubleICAM-1
9: 135120874–135140438	3551		8.83E-26	0.43	rs579459	Soluble E-selectinlevels
9: 135120874–135140438	4250		7.93E-25	0.43	rs495828	Angiotensin-convertingenzyme activity
15: 43458687–43481812	30170		1.24E-21	0.14	rs2453533^11^	Chronickidney disease
15: 82975686–82986699	245274		4.86E-13	0.05	rs3743162^12^	Alzheimer’sdisease (ageof onset)
17: 26060783–26121168	150673	H3K4m1	1.28E-8	0.06	rs3760318	Height

1 The distance to the lncRNA region is 0 if the SNP is located within the region and the smallest distance otherwise,

2 Slope is given in absolute numbers,

3 Listed are all cis-rSNPs that are also found in the GWAS catalog together with the associated lncRNA,

4 The trait is taken from the GWAS catalog,

5 Within 2.5 kb,

6 rs6663565 as proxy,

7 rs2303393 as proxy,

8 rs6922111 as proxy,

9 rs7739434 as proxy,

10 rs13214831 as proxy,

11 rs1153862 as proxy,

12 rs12442557 as proxy.

For Refseq protein coding regions we found 458 loci where a reported GWAS SNP overlaps with a *cis*-rSNP or a proxy *cis*-rSNP in high LD (31 SNPs with LD 1.0 and 29 with LD>0.81 (Table S2). As for the lncRNAs a high fraction (n = 112) of the traits are related to immune diseases.

## Discussion

The combination of a large number of samples from primary monocytes and the sensitive genotyping method for detecting *cis*-regulatory SNPs based on allele-specific gene expression [4] renders our study well powered for detecting *cis*-regulatory SNPs that affect expression of long non-coding RNAs, which are expressed at a lower level than protein-coding genes. In the ASE approach transcript are detected using a genome-wide panel of SNPs markers, and hence the use of predefined hybridization probes for each transcript is circumvented. This unbiased detection of expressed transcripts is an advantage of ASE analysis over expression microarrays. This advantage is shared by ASE and “next” generation transcriptome sequencing (RNA-seq). An advantage of RNA-seq compared to ASE analysis using SNP genotyping is that alternatively spliced transcripts and strand specific gene expression can be detected, provided that the sequencing coverage is sufficient. However, ASE analysis using RNA-seq suffers from the drawback of being highly affected by the reference bias [31], [32], although methods that can correct for most of the bias have been developed recently [33], [34]. Moreover, lowly expressed genes obtain very low coverage in RNA-seq.

In this study we focused our ASE analysis of monocytes on 5346 lncRNAs that were annotated by the ENCODE project as intergenic without overlap with protein coding genes. Thus we ensured that our analysis targeted truly lncRNA genes and was not confounded by overlapping protein-coding genes. Using stringent criteria for calling ASE, we identified 258 lncRNAs that were regulated by at least one *cis*-rSNP. We observed correlated expression of 20% of the lncRNAs with their neighbouring protein-coding genes, and co-regulation of a lncRNA and a neighbouring protein coding gene by the same *cis*-rSNP for approximately half of these lncRNA genes. This finding is consistent with a recent study of peripheral blood cells, where 75% of 112 lncRNAs that were mapped using traditional eQTL analysis were found to be regulated independently of nearby protein-coding genes [19]. Because the ASE analysis used in our study only detects *cis*-regulation, ASE analysis allows the dissection of *cis*-effects by lncRNAs that influence the transcription of nearby genes. If the expression level of a lncRNA and a nearby protein coding gene are correlated, and they both show significant ASE association with the same *cis*-rSNP, it is likely that the *cis*-rSNP directly regulates the protein coding gene. If the expression levels between a protein-coding and lncRNA gene are correlated, but there is no shared association with a *cis*-SNP, the lncRNA may be a regulatory factor that affects the expression of the adjacent protein coding gene. In our ASE data set we found that these two putative mechanisms occur at similar frequencies. However, using the ASE approach, trans-acting regulatory mechanisms for co-expressed genes would remain undetected. A recent study used traditional eQTL analysis of monocytes found several trans-regulated modules of co-expressed protein coding genes. However, lncRNA expression was not addressed in this study [35].

A drawback of our study is that this specific dataset was not replicated by an independent method. However, in a previous study we have shown that a similar method for genome-wide ASE-analysis using NS-12 BeadChips (Illumina) was highly reproducible between replicate samples, with correlation coefficients of 0.9969 for gDNA and 0.9956 for cDNA. ASE-levels determined using NS-12 BeadChips and those determined by quantitative Sanger sequencing were also strongly correlated (0.86) in nine representative genes [3]. Furthermore, in the current study the consistency of the ASE-signal for individual SNPs across the lncRNA regions is high, with an average standard deviation of 0.05 over all regions and samples. The consistency of the ASE-signals can be observed in Figure S4, where almost all SNPs in the lncRNA regions show overexpression of the same allele.

In our study we found that 32 of the *cis*-rSNPs that regulate the expression of lncRNAs have been identified in GWAS as risk variants for human diseases or traits. The diseases or phenotypic traits associated with these *cis*-regulatory SNPs according to the lncRNA ASE analysis, include diseases of the immune system; multiple sclerosis, Graves’ disease, eosinophilic esophagitis, celiac disease, rheumatoid arthritis, immune response to small pox vaccine, soluble E-selectin levels, soluble ICAM-1, and ankylosing spondylitis, for which monocytes are a relevant cell type (Table 1). Chronic kidney disease is also a relevant trait as it can be caused both by the autoimmune disease type 1 diabetes and by hypertension, which are diseases for which monocytes are a relevant cell type [25]. By manually classifying the traits in the GWAS catalog into two classes, *ie* traits related to the immune system, and other traits, we tested for over-representation of *cis*-rSNPs in the GWAS-catalog using Fisher’s exact test. For traits associated to the immune system, the p-value for overrepresentation is 0.00027. The SNP rs13278062 that confers risk of age-related macular degeneration detected in our study of monocytes using ASE analysis (Table 1) was also highlighted by traditional eQTL analysis of peripheral blood samples [19], while none of the other detected risk SNPs or traits overlapped between the two studies.

We also found that the identified *cis*-rSNPs for lncRNA are enriched to active enhancer regions in monocytes, which suggests a mechanism for their *cis*-regulatory functions. The reported 32 risk SNPs from GWAS that are strongly associated with the expression of non-coding RNAs provides interesting leads for further characterization and functional clues into immune diseases. Some of the GWAS SNPs are located in enhancer regions, which could be the cause of the allele specific expression of the lncRNA. Thus our study suggests more complex functional mechanisms underlying findings from GWAS than regulatory variants or expression levels of nearby protein coding genes, and provides novel insights into the relationship between genetic variation and human diseases.

## Supporting Information

Figure S1 **Schematic picture of the principles ASE analysis.** In ASE the allele-specific expression level is measured by the difference in fluorophore signal intensity between the two alleles in the same sample. The average ASE-level is calculated for all heterozygous SNPs in the region. This ASE-value is then used in an association test against the genotypes of *cis*-rSNPs (shown in yellow). Figure adapted from Almlöf *et al* [4].
(PDF)

Figure S2 **Signal intensity threshold.** The figure shows the distribution of signal intensities for the two alleles. The x-channel represents the A-allele and the y-channel represents the B-allele. In the xraw panel the fraction of SNPs with the BB genotype having intensity levels above the cutoff level of 1000 is very low and similarly for the yraw panel.
(PDF)

Figure S3 **Regression lines.** Regression lines for the 32 risk SNPs from GWAS that are significantly associated with a lncRNA region.
(PDF)

Figure S4 **lncRNA in the GWAS catalog.** Illustration of all lncRNA regions that have a significant association to a SNP that is also significantly associated in the GWAS catalog. The tracks are from top to bottom in each panel: Horizontal red bars represent transcript windows (with genomic coordinates) used for determination of ASE levels; grey lines show p-values for the association of GWAS SNPs with ASE levels in the transcript window; a grey line overlayed with a red dotted line indicates that this is the SNP that overlaps with the reported SNP in the GWAS catalog; red vertical lines are median ASE-levels for each SNP; annotated transcripts are shown in black below the tracks.
(PDF)

Table S1 **Significantly associated lncRNA regions.**
(DOCX)

Table S2 **SNPs associated with allele-specific expression of Refseq protein coding genes with published trait- or disease-associations from genome-wide association studies.**
(DOCX)

Methods S1 **Quadratic normalization.** Detailed explanation of the quadratic normalization performed on the intensity levels obtained from the genotyping.
(DOCX)
