# Supplementary figures and images for: Single Nucleotide Polymorphisms with Cis-Regulatory Effects on Long Non-Coding Transcripts in Human Primary Monocytes
Source: PLoS One. 2014 Jul 15;9(7):e102612. doi: 10.1371/journal.pone.0102612 (PMC4099216; doi:10.1371/journal.pone.0102612)

## Allele specific expression (ASE)

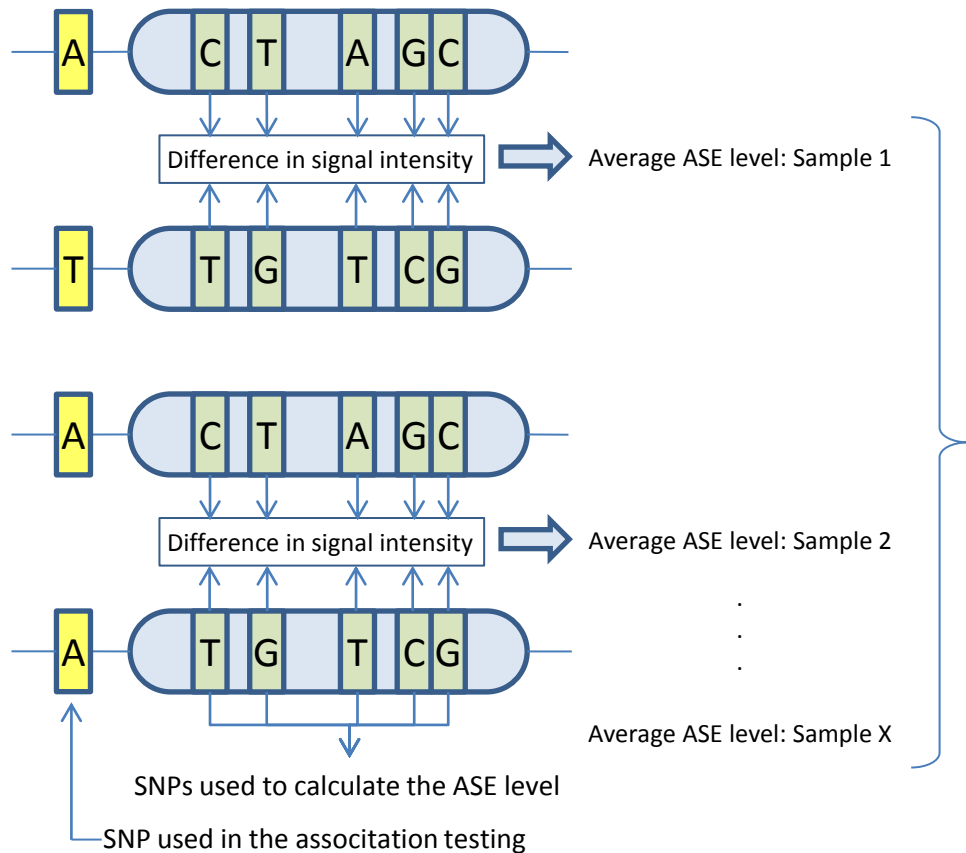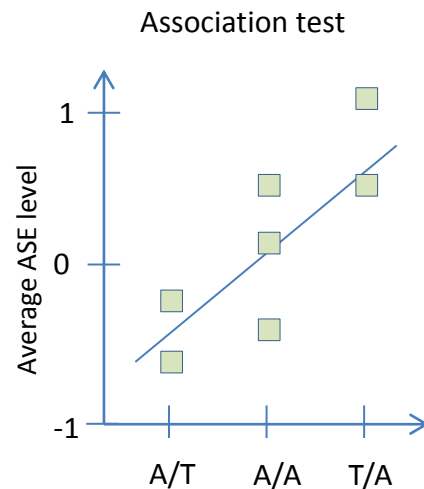

Supplement: Figure S1 — Schematic picture of the principles ASE analysis. In ASE the allele-specific expression level is measured by the difference in fluorophore signal intensity between the two alleles in the same sample. The average ASE-level is calculated for all heterozygous SNPs in the region. This ASE-value is then used in an association test against the genotypes of cis-rSNPs (shown in yellow). Figure adapted from Almlöf et al [4]. (PDF) [file pone.0102612.s001.pdf]

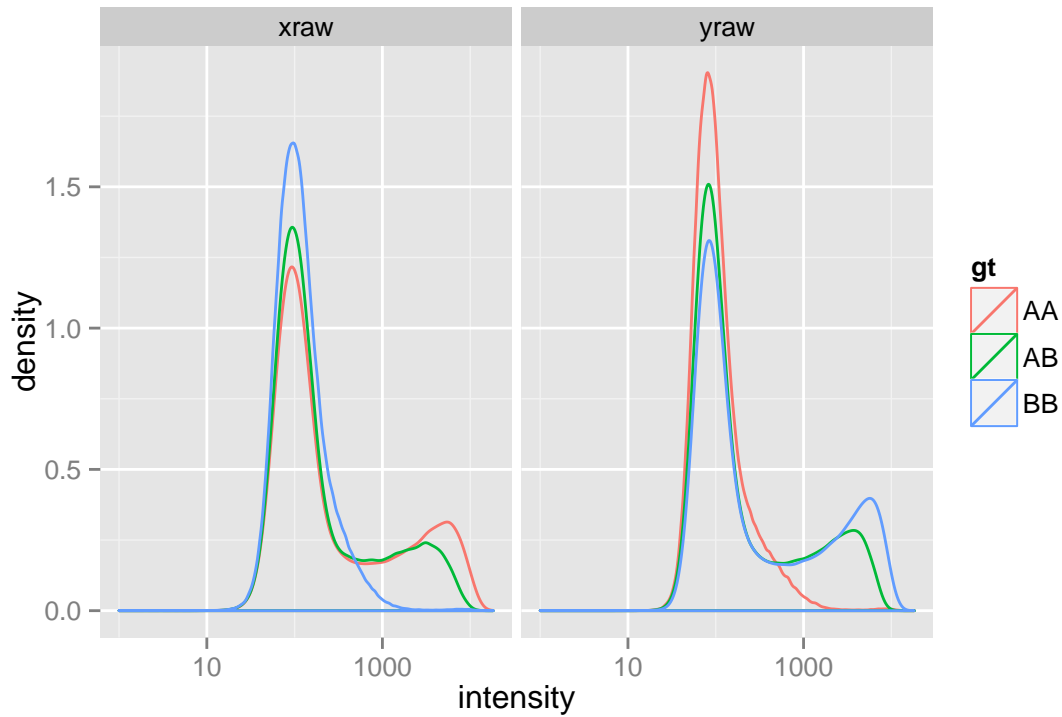

Supplement: Figure S2 — Signal intensity threshold. The figure shows the distribution of signal intensities for the two alleles. The x-channel represents the A-allele and the y-channel represents the B-allele. In the xraw panel the fraction of SNPs with the BB genotype having intensity levels above the cutoff level of 1000 is very low and similarly for the yraw panel. (PDF) [file pone.0102612.s002.pdf]

1 rs2501276

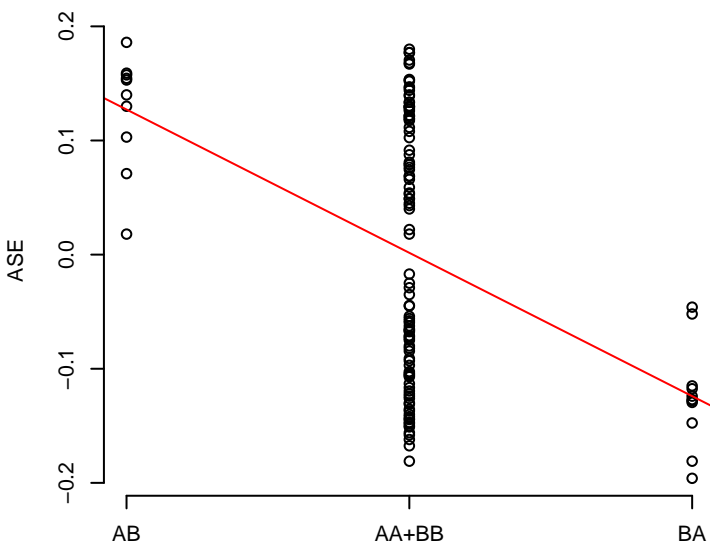

2 rs16826658

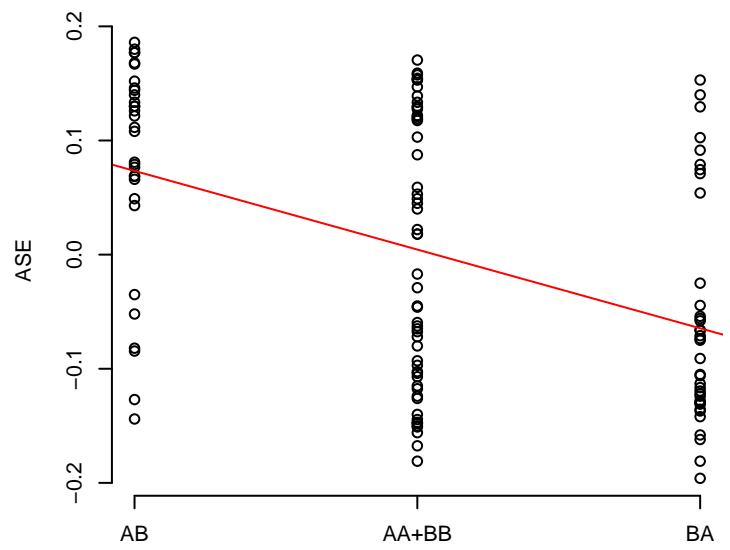

3 rs6663565

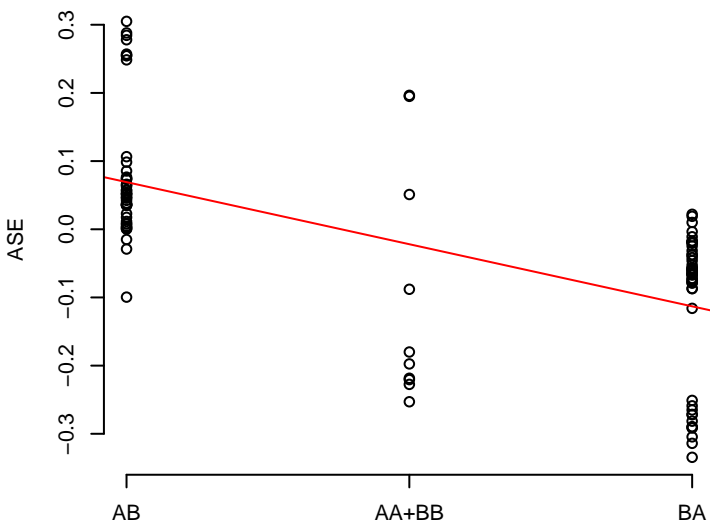

4 rs6687758

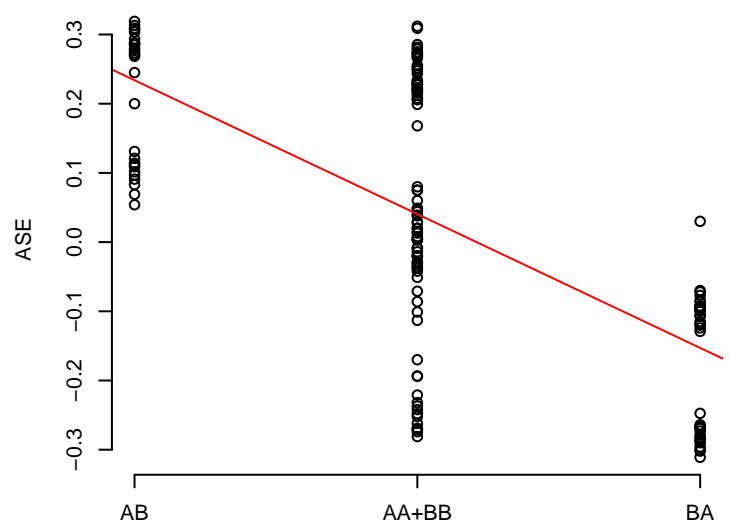

5 rs1876040

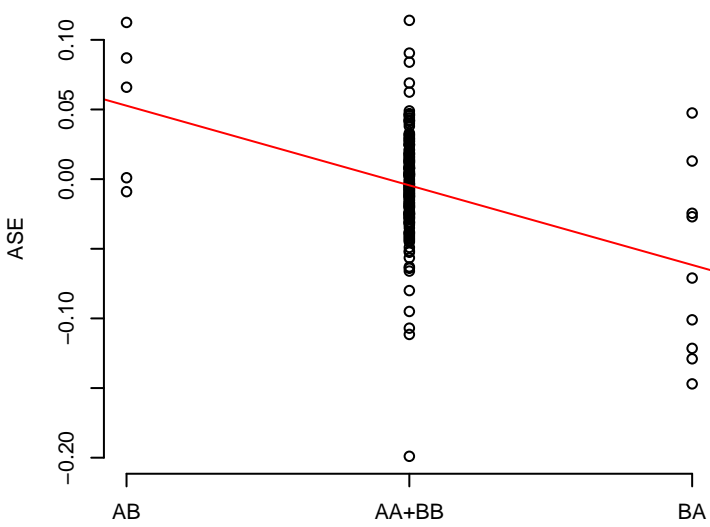

6 rs2310173

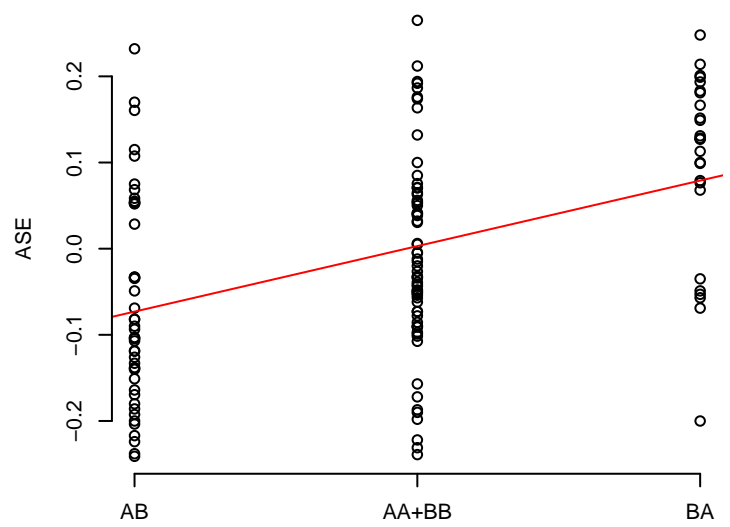

7 rs6710518

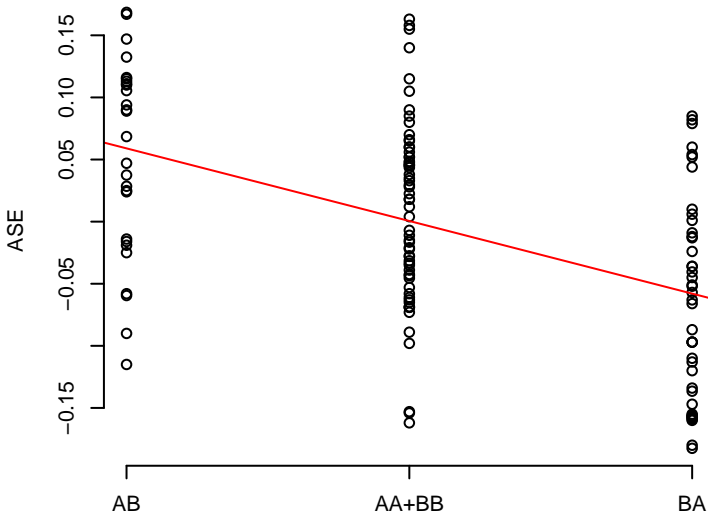

8 rs2303393

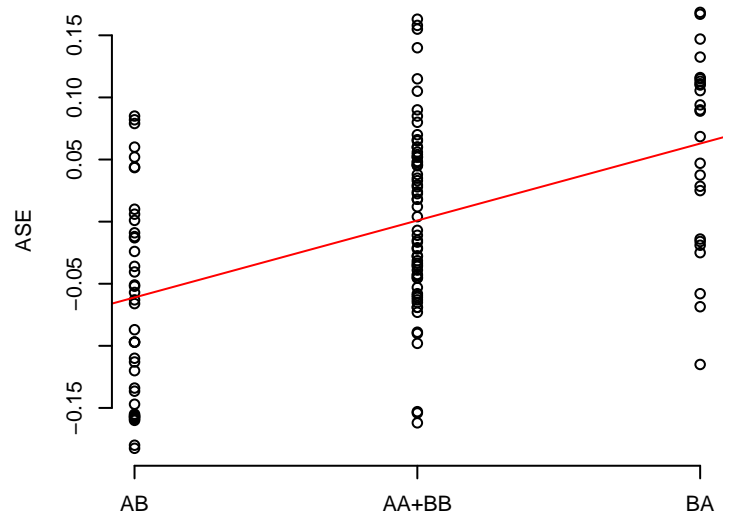

9 rs12638253

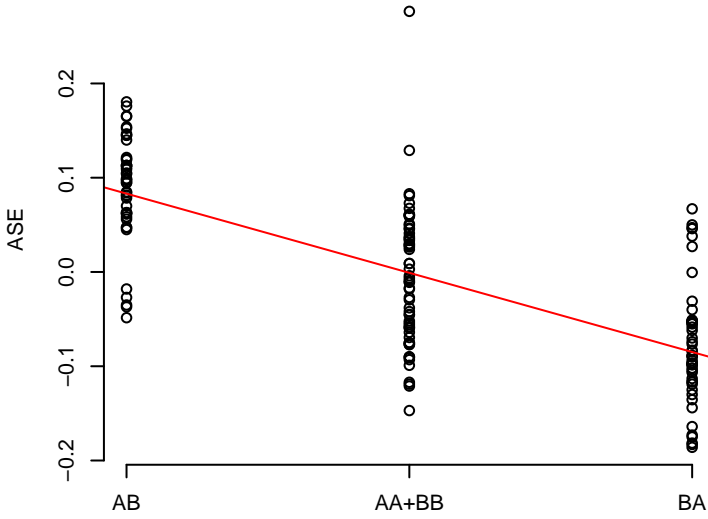

10 rs7686660

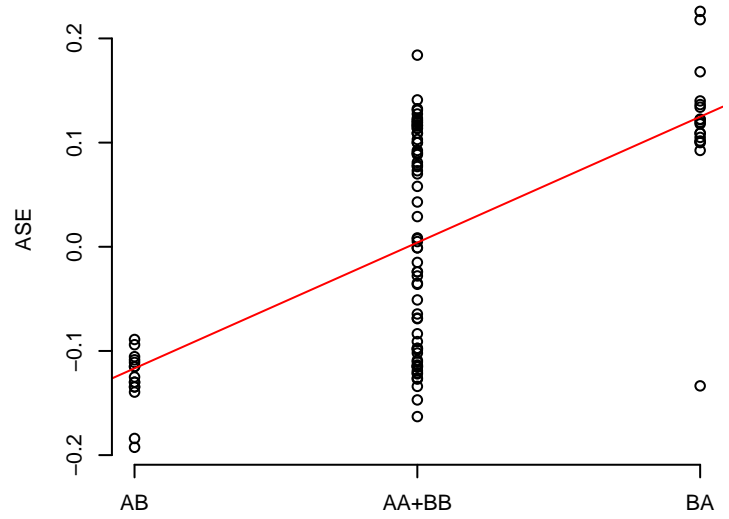

11 rs6922111

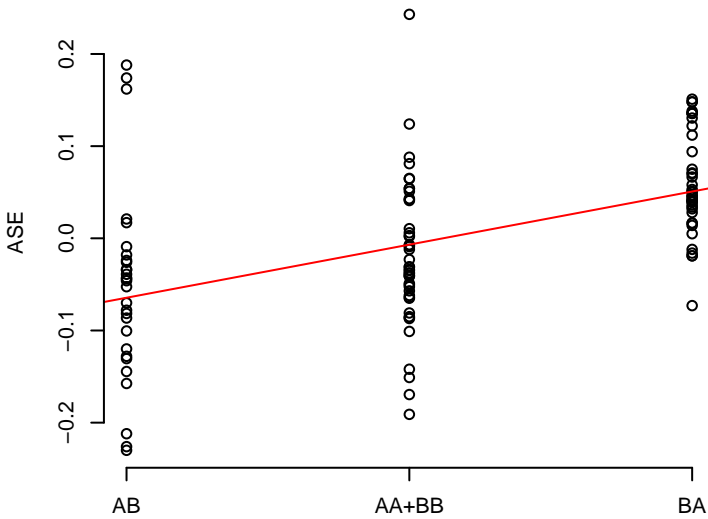

12 rs3893464

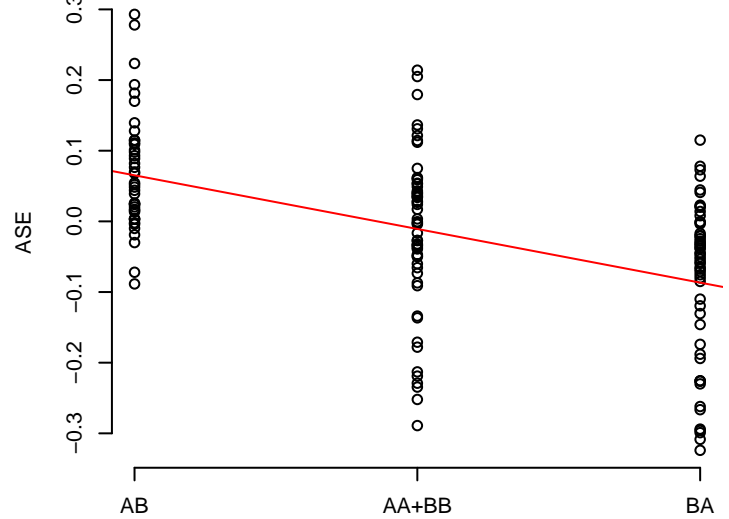

13 rs7739434

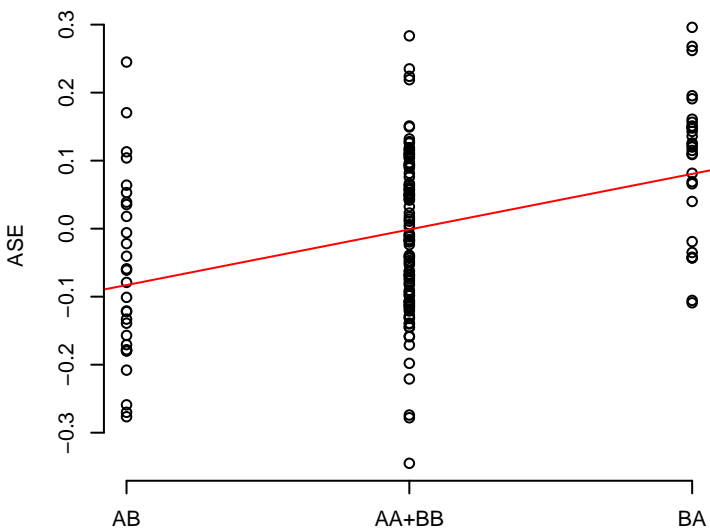

14 rs13214831

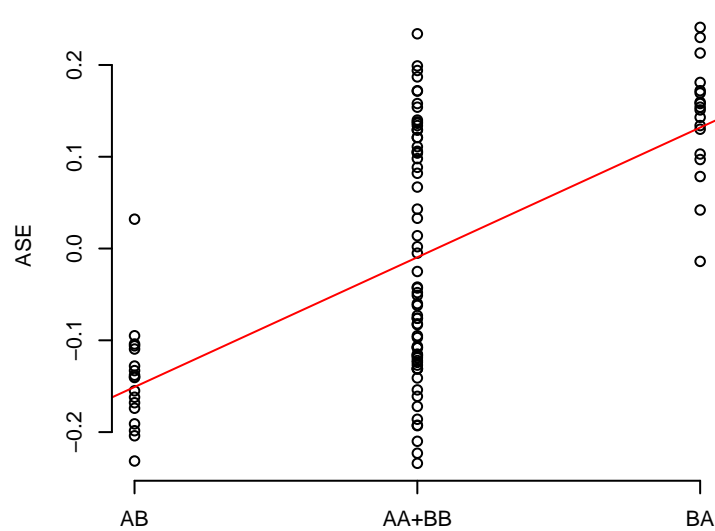

15 rs4248154

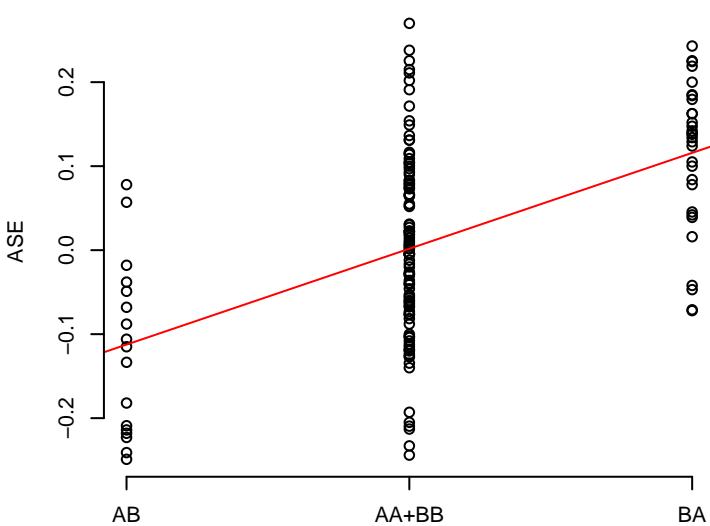

16 rs4928431

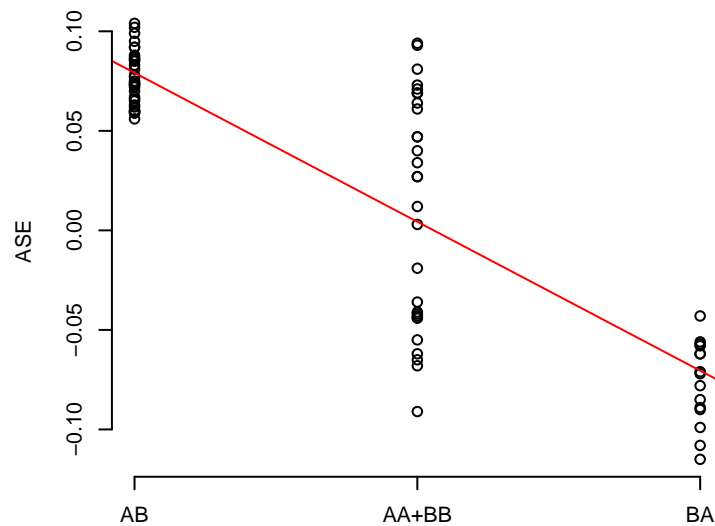

17 rs10256972

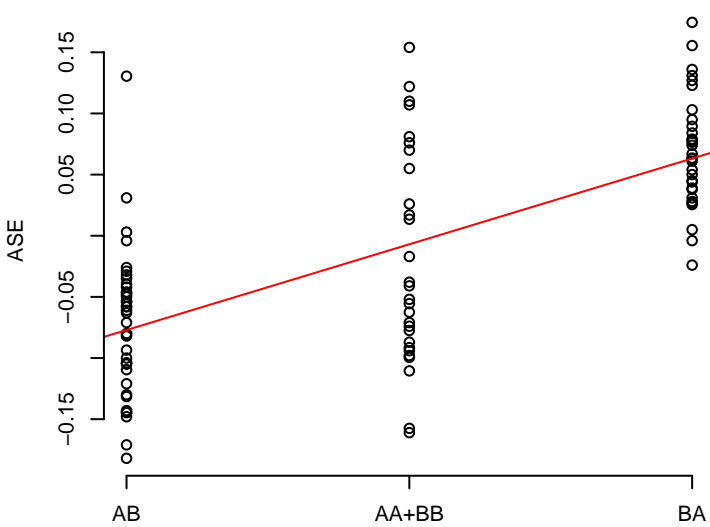

18 rs10259085

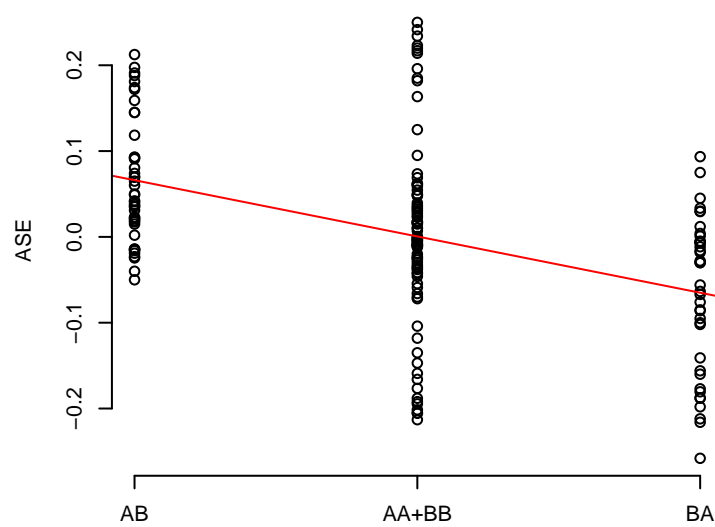

19 rs1299548

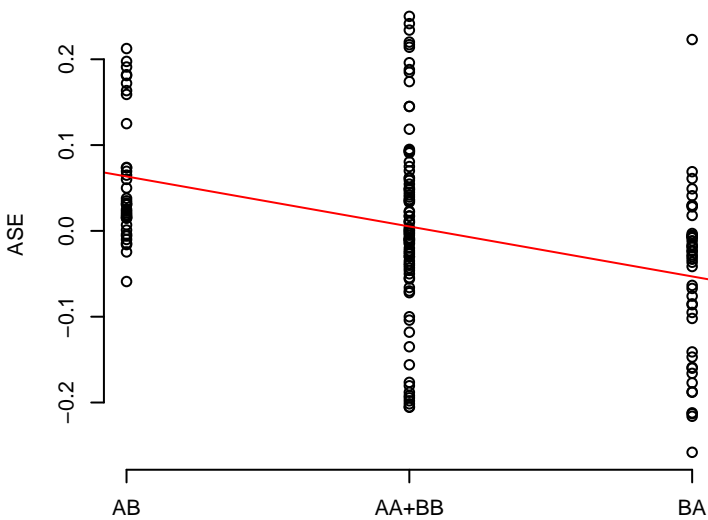

20 rs13278062

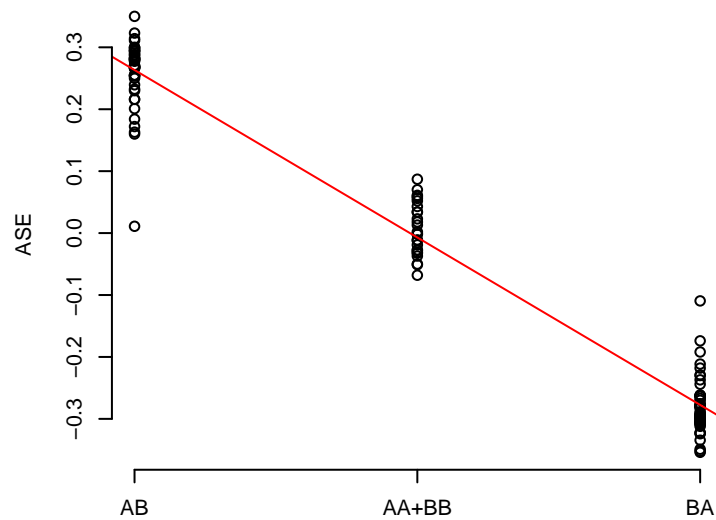

21 rs1953126

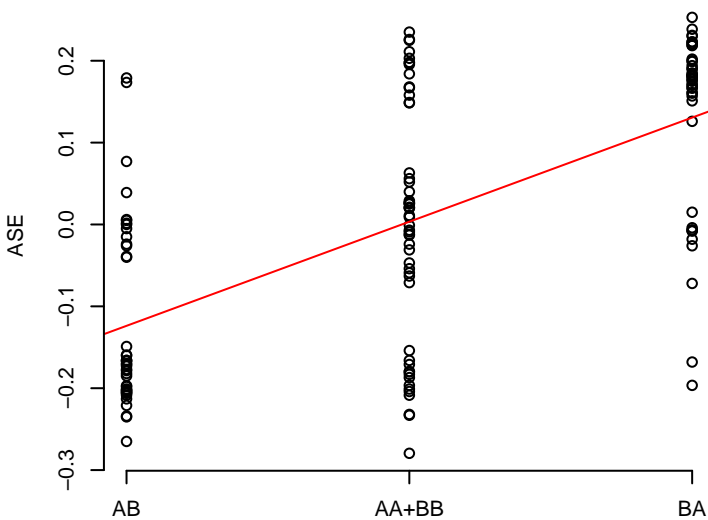

22 rs881375

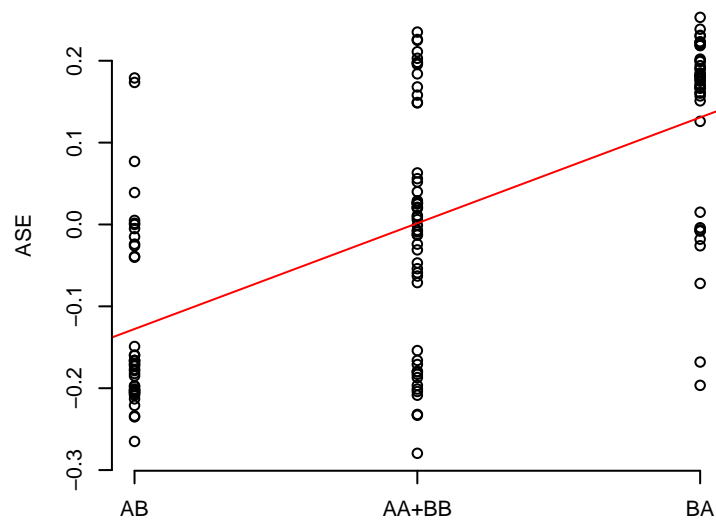

23 rs3761847

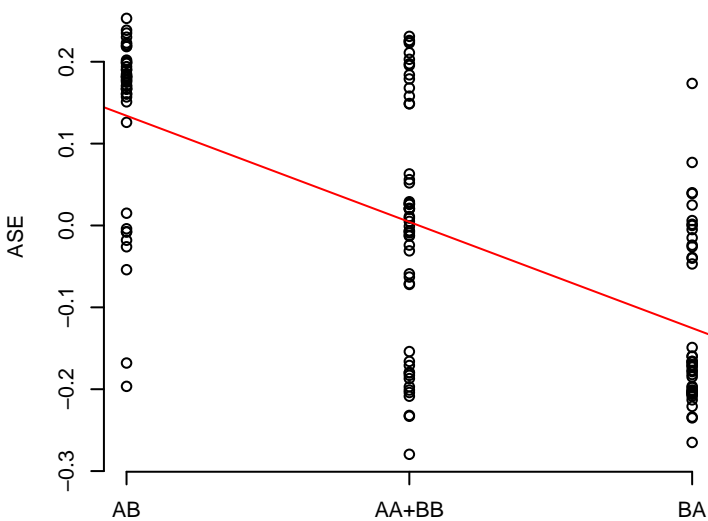

24 rs687621

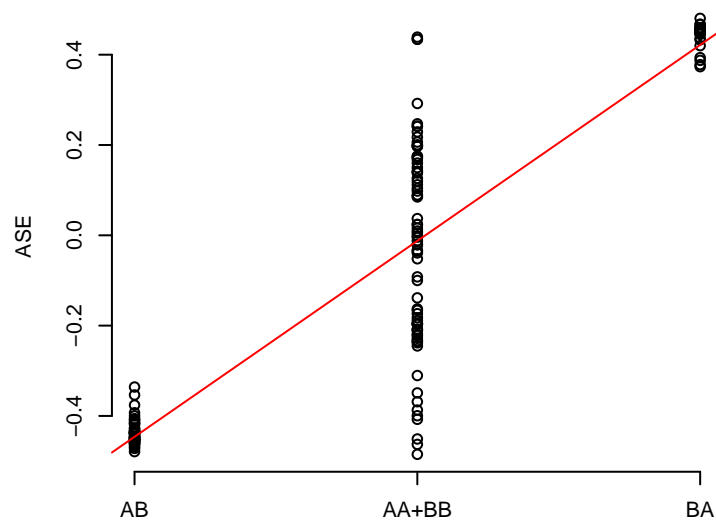

25 rs657152

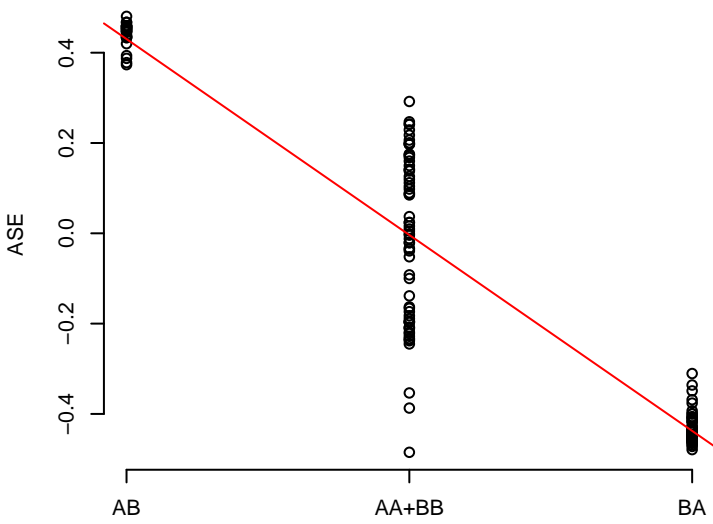

26 rs643434

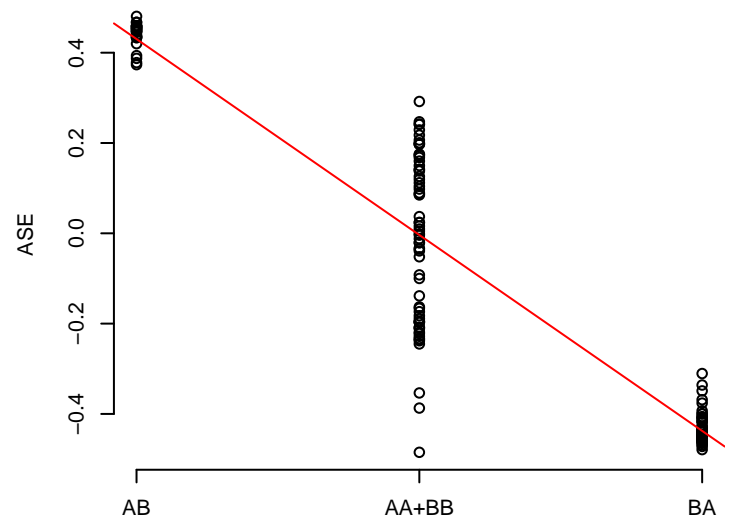

27 rs612169

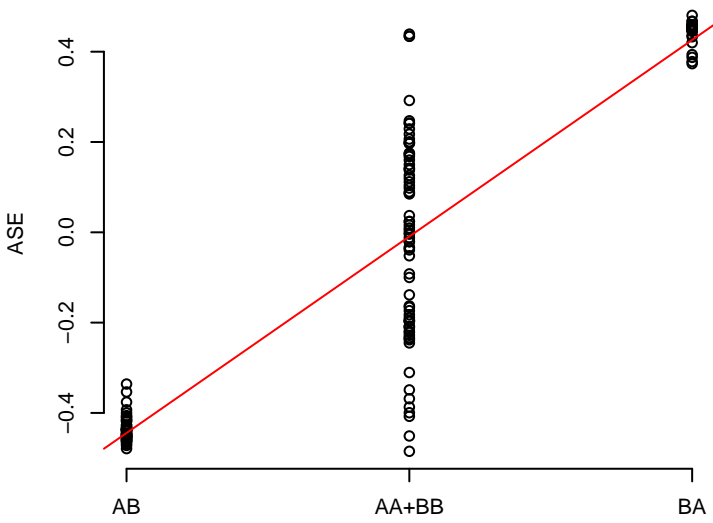

28 rs505922

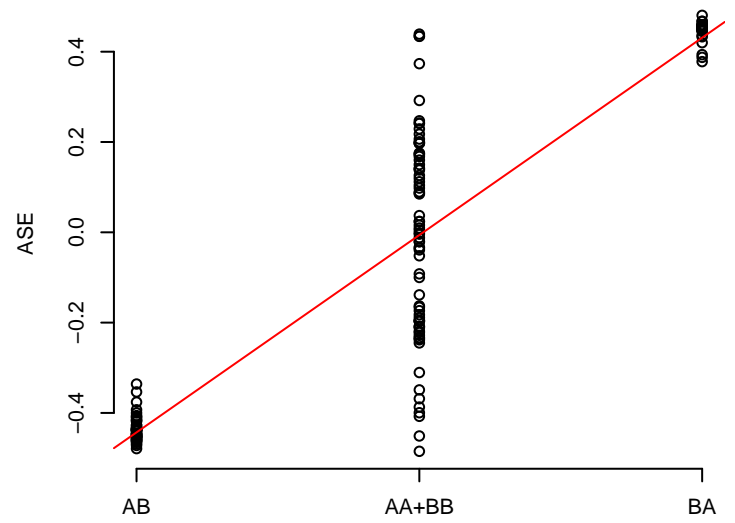

29 rs507666

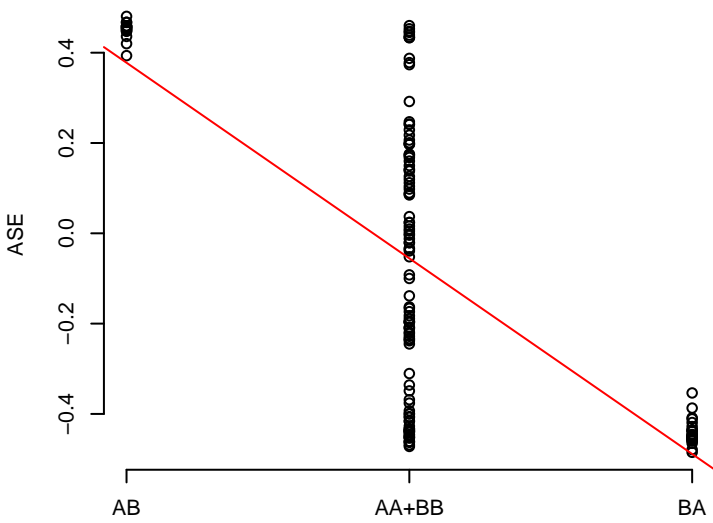

30 rs579459

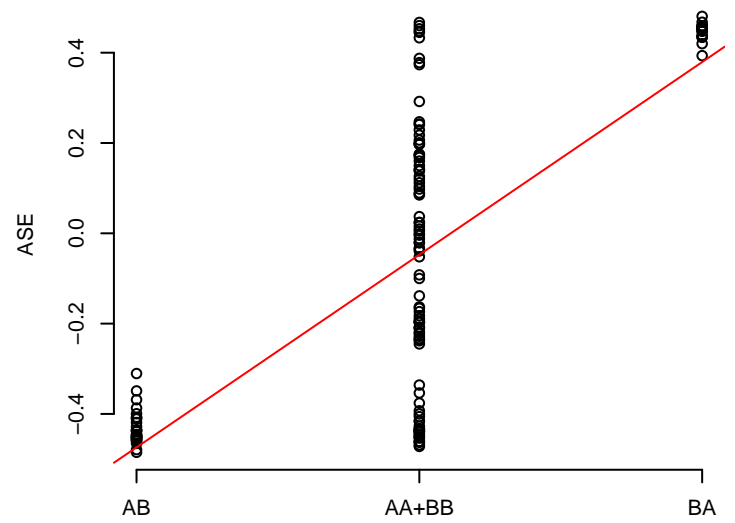

31 rs495828

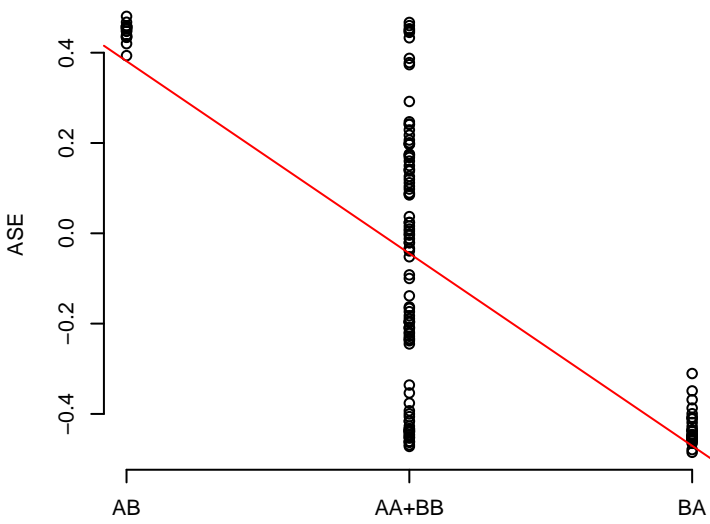

32 rs9423406

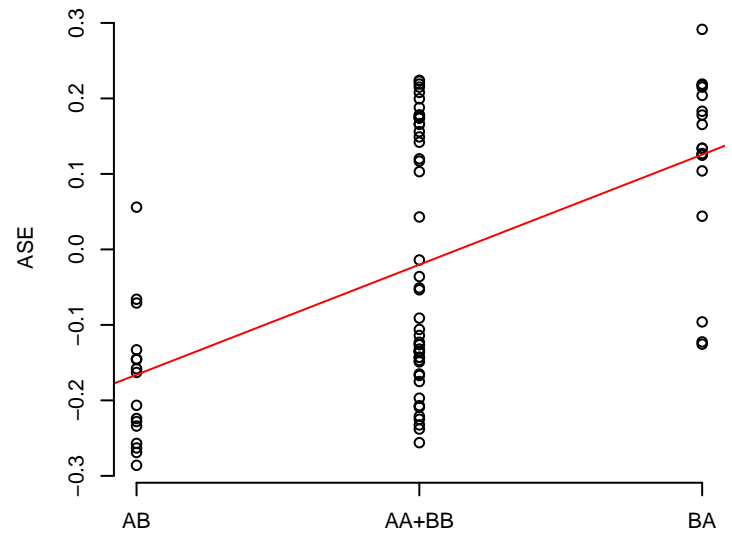

33 rs7896729

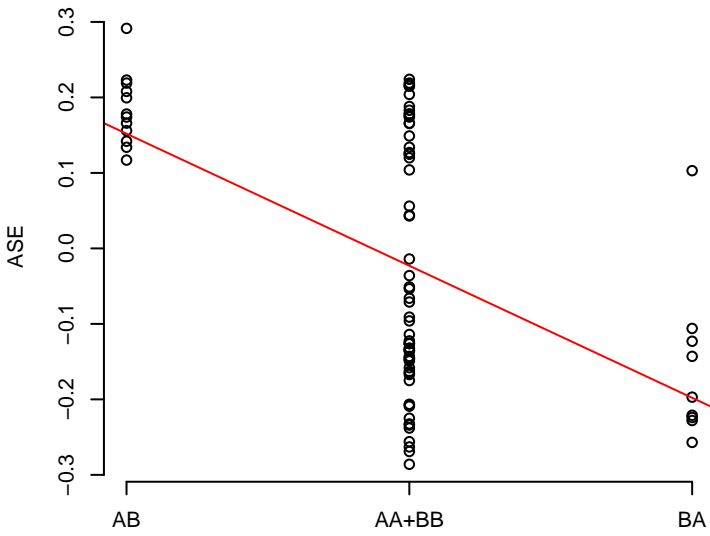

34 rs1153862

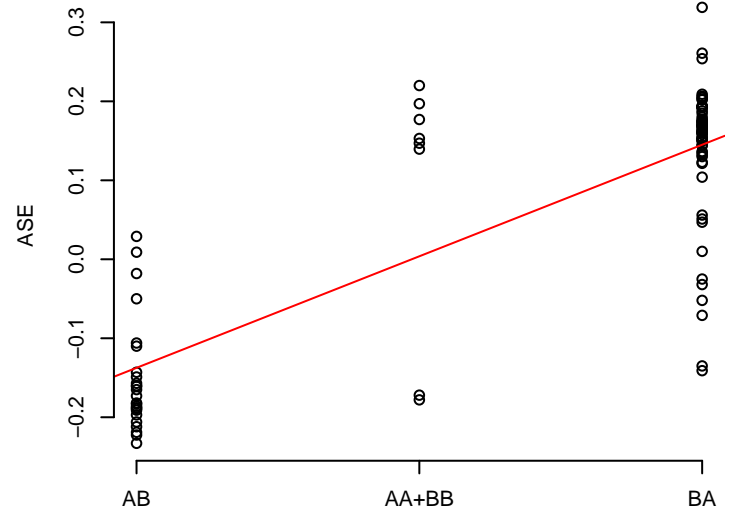

35 rs12442557

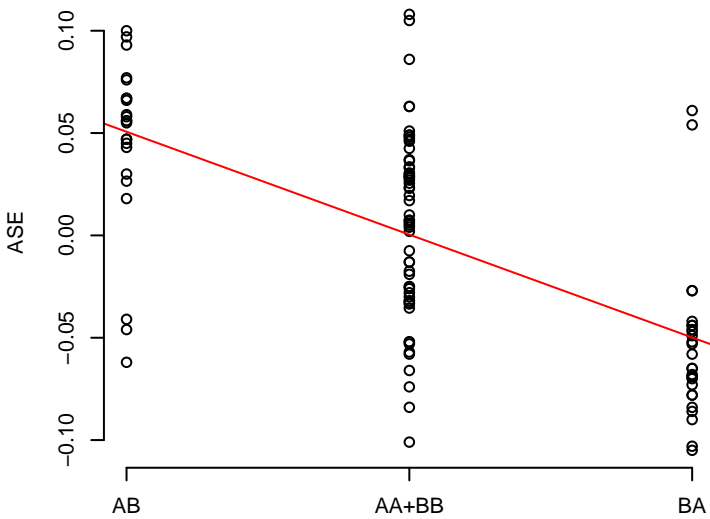

36 rs3760318

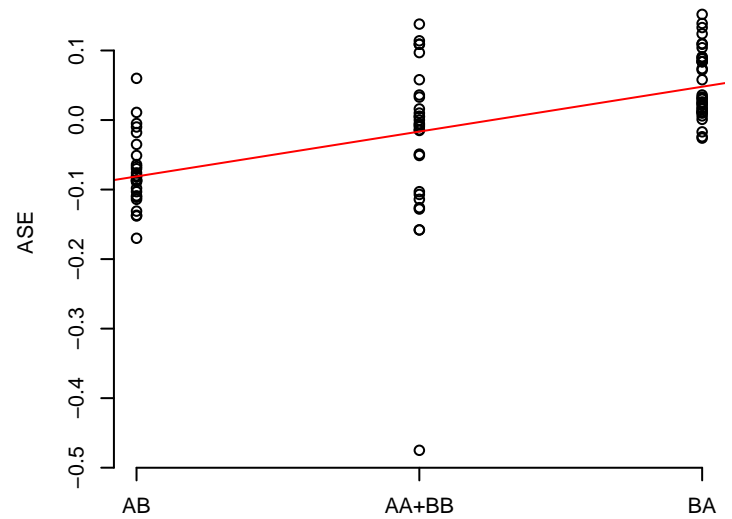

Supplement: Figure S3 — Regression lines. Regression lines for the 32 risk SNPs from GWAS that are significantly associated with a lncRNA region. (PDF) [file pone.0102612.s003.pdf]
