# Supplementary material for: Single Nucleotide Polymorphisms with Cis-Regulatory Effects on Long Non-Coding Transcripts in Human Primary Monocytes
Source: PLoS One. 2014 Jul 15;9(7):e102612. doi: 10.1371/journal.pone.0102612 (PMC4099216; doi:10.1371/journal.pone.0102612)
Supplement: Figure S4 — lncRNA in the GWAS catalog. Illustration of all lncRNA regions that have a significant association to a SNP that is also significantly associated in the GWAS catalog. The tracks are from top to bottom in each panel: Horizontal red bars represent transcript windows (with genomic coordinates) used for determination of ASE levels; grey lines show p-values for the association of GWAS SNPs with ASE levels in the transcript window; a grey line overlayed with a red dotted line indicates that this is the SNP that overlaps with the reported SNP in the GWAS catalog; red vertical lines are median ASE-levels for each SNP; annotated transcripts are shown in black below the tracks. (PDF) [file pone.0102612.s004.pdf]

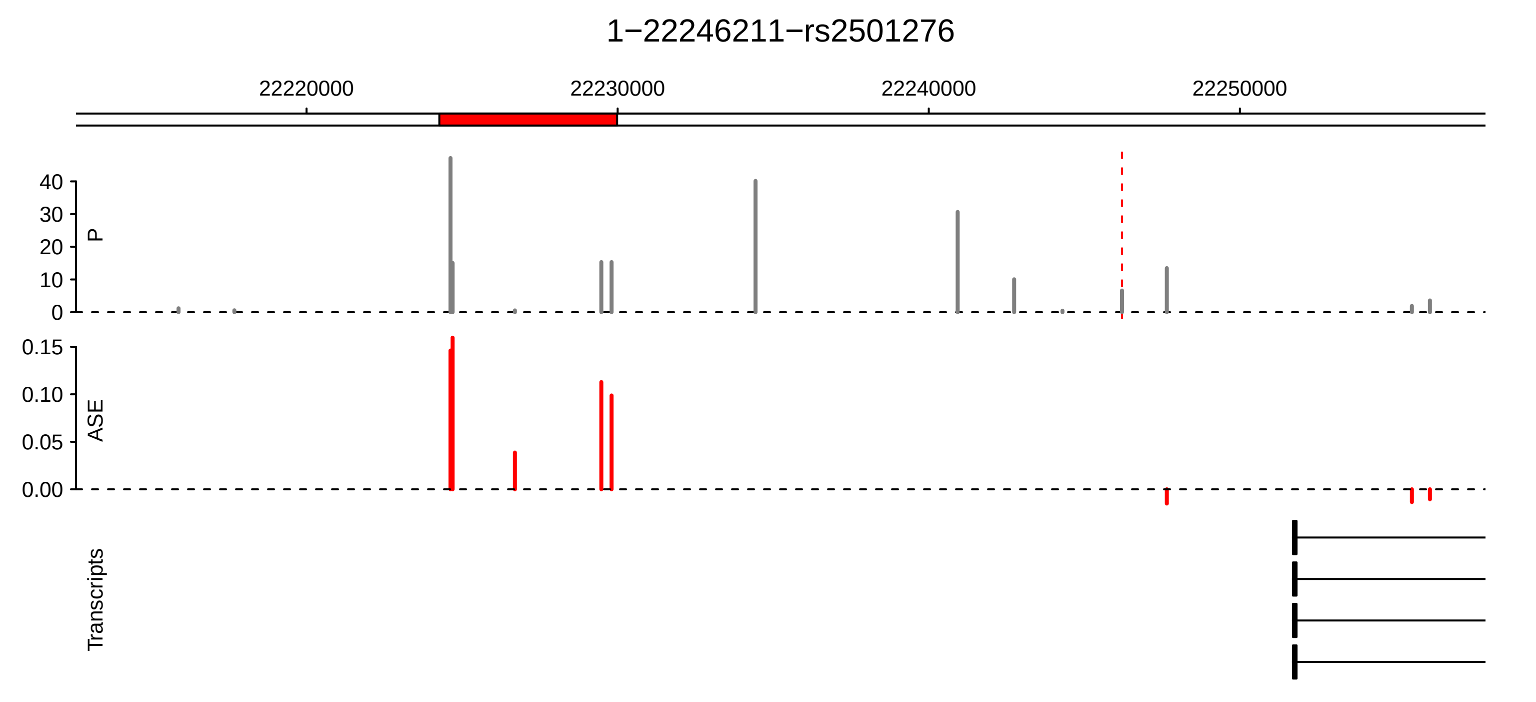

1−22246211−rs2501276
22220000
22230000
22240000
22250000
P
40
30
20
10
0
ASE
0.15
0.10
0.05
0.00
Transcripts

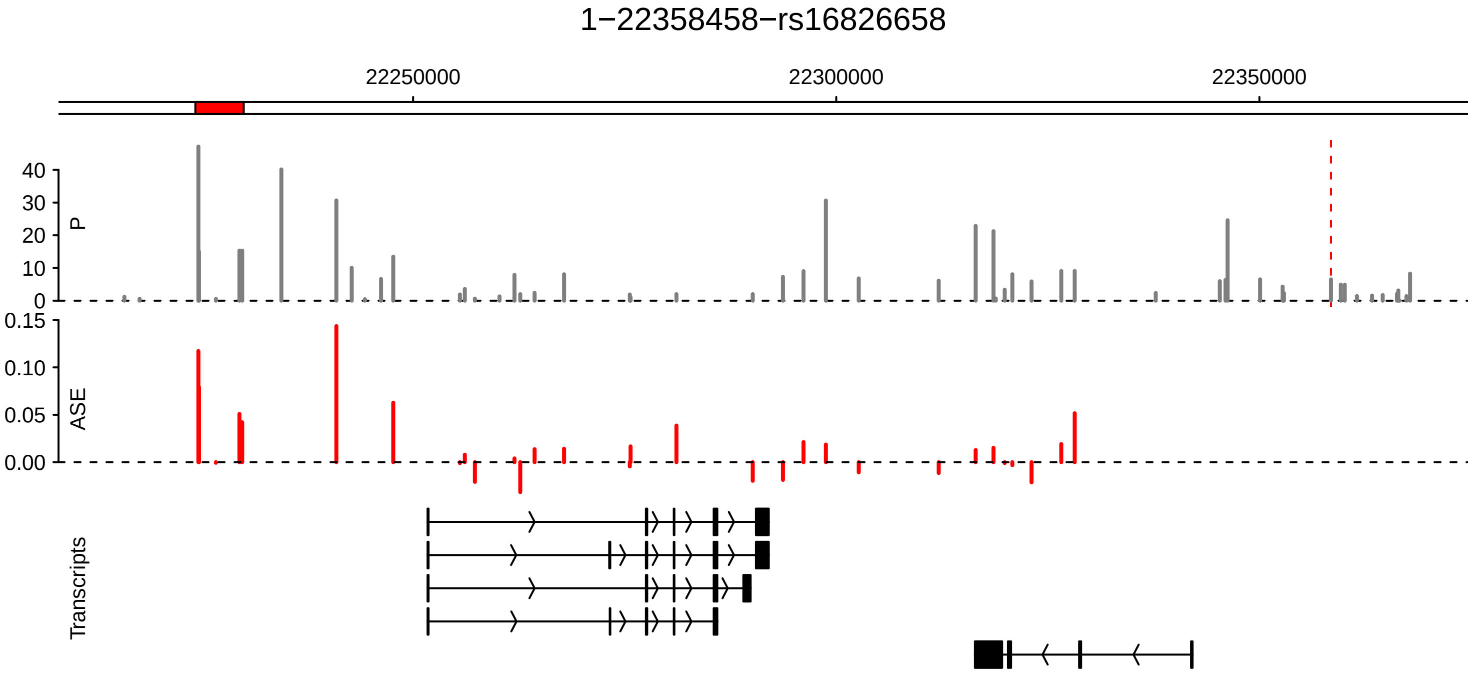
1−22358458−rs16826658
22250000
22300000
22350000
P
40
30
20
10
0
ASE
0.15
0.10
0.05
0.00
Transcripts

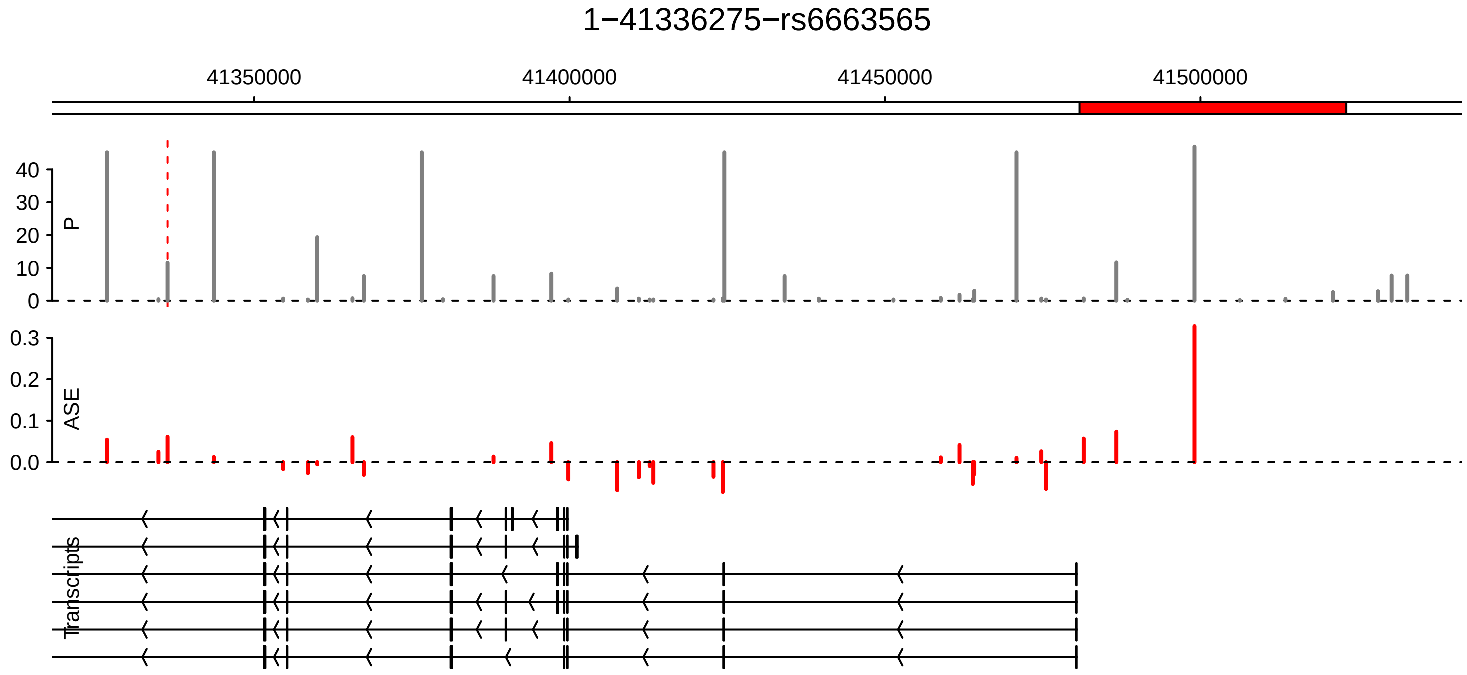
1−41336275−rs6663565
41350000
41400000
41450000
41500000
P
40
30
20
10
0
ASE
0.3
0.2
0.1
0.0
Transcripts

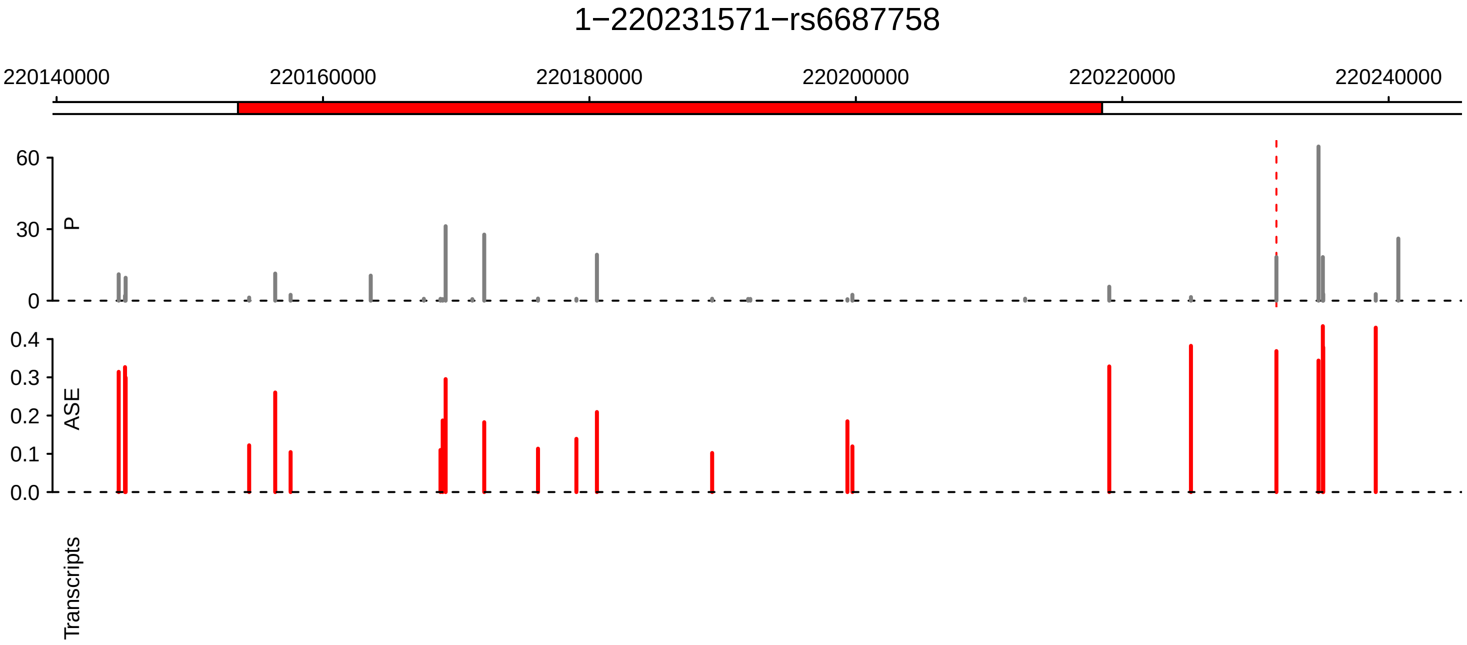
1−220231571−rs6687758
220140000
220160000
220180000
220200000
220220000
220240000
60
30
0
P
0.4
0.3
0.2
0.1
0.0
ASE
Transcripts

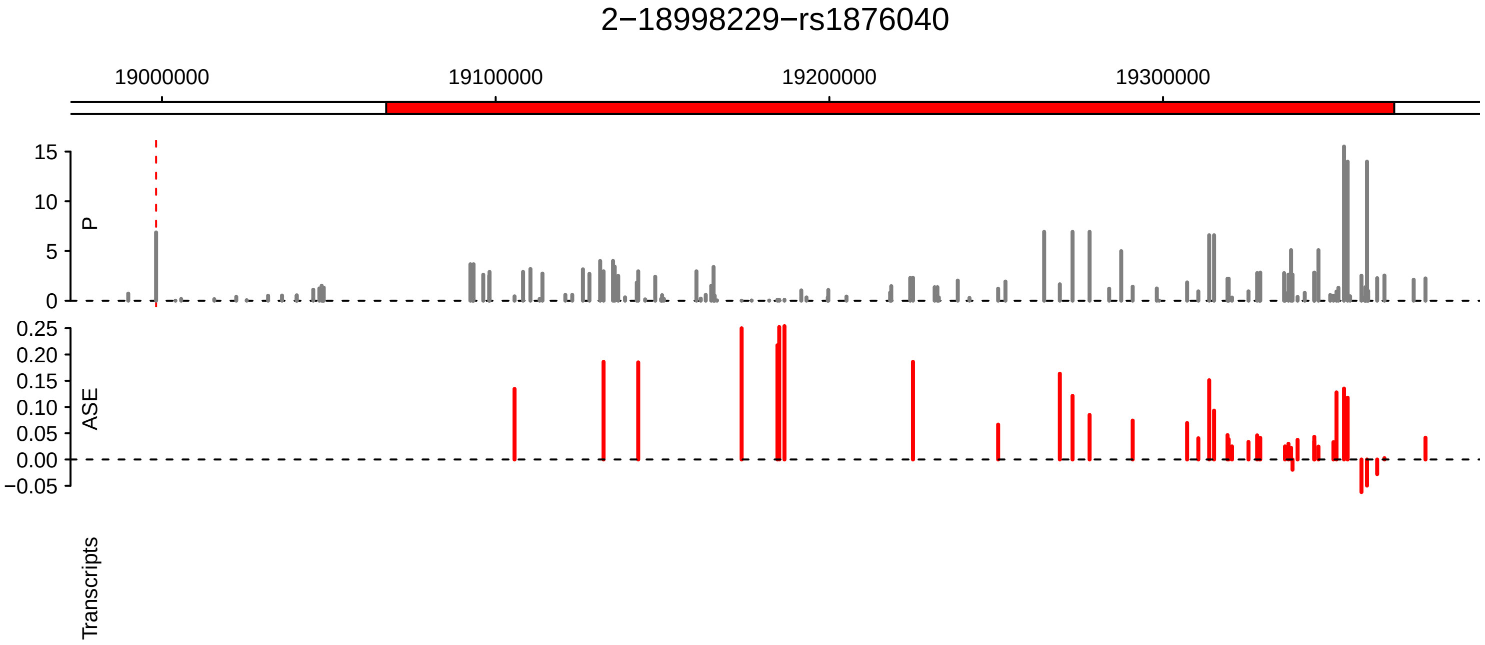

2−18998229−rs1876040
19000000
19100000
19200000
19300000
P
15
10
5
0
ASE
0.25
0.20
0.15
0.10
0.05
0.00
−0.05
Transcripts

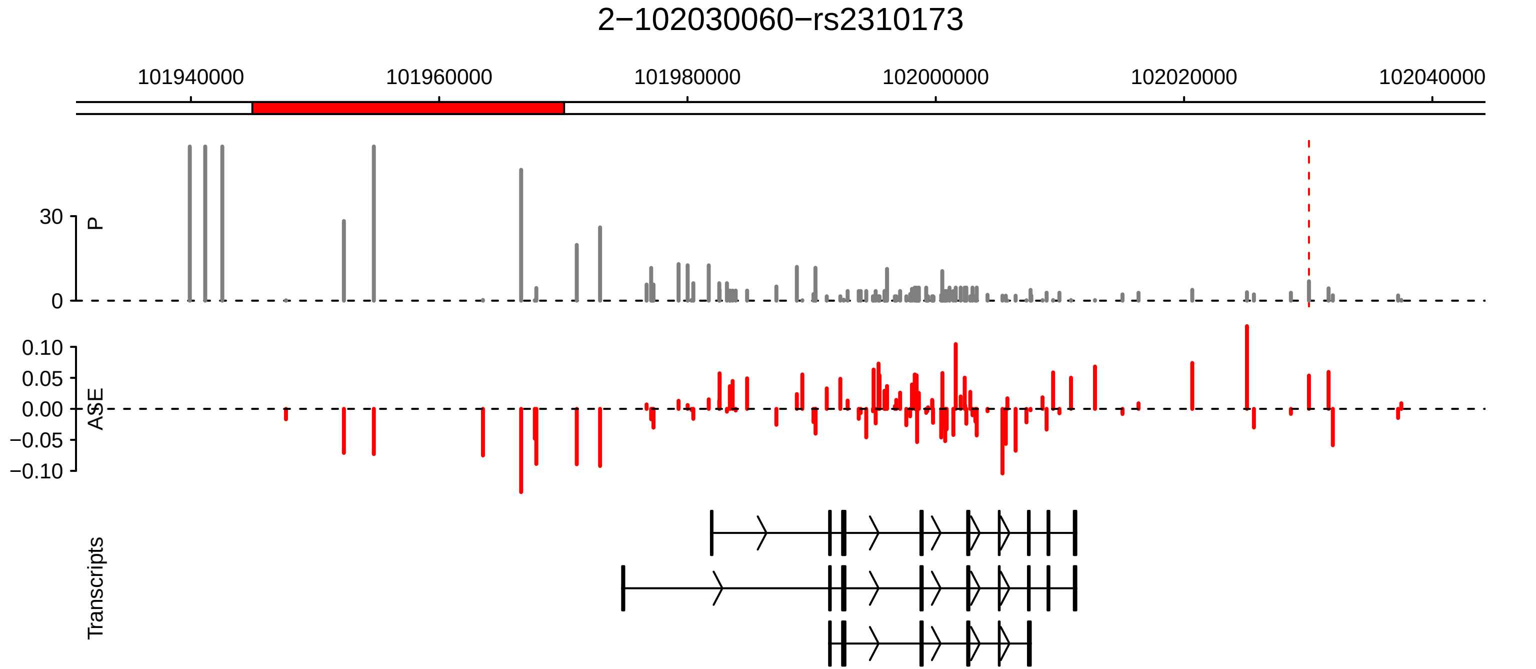
2−102030060−rs2310173
101940000
101960000
101980000
102000000
102020000
102040000
30
0
P
0.10
0.05
0.00
−0.05
−0.10
ASE
Transcripts

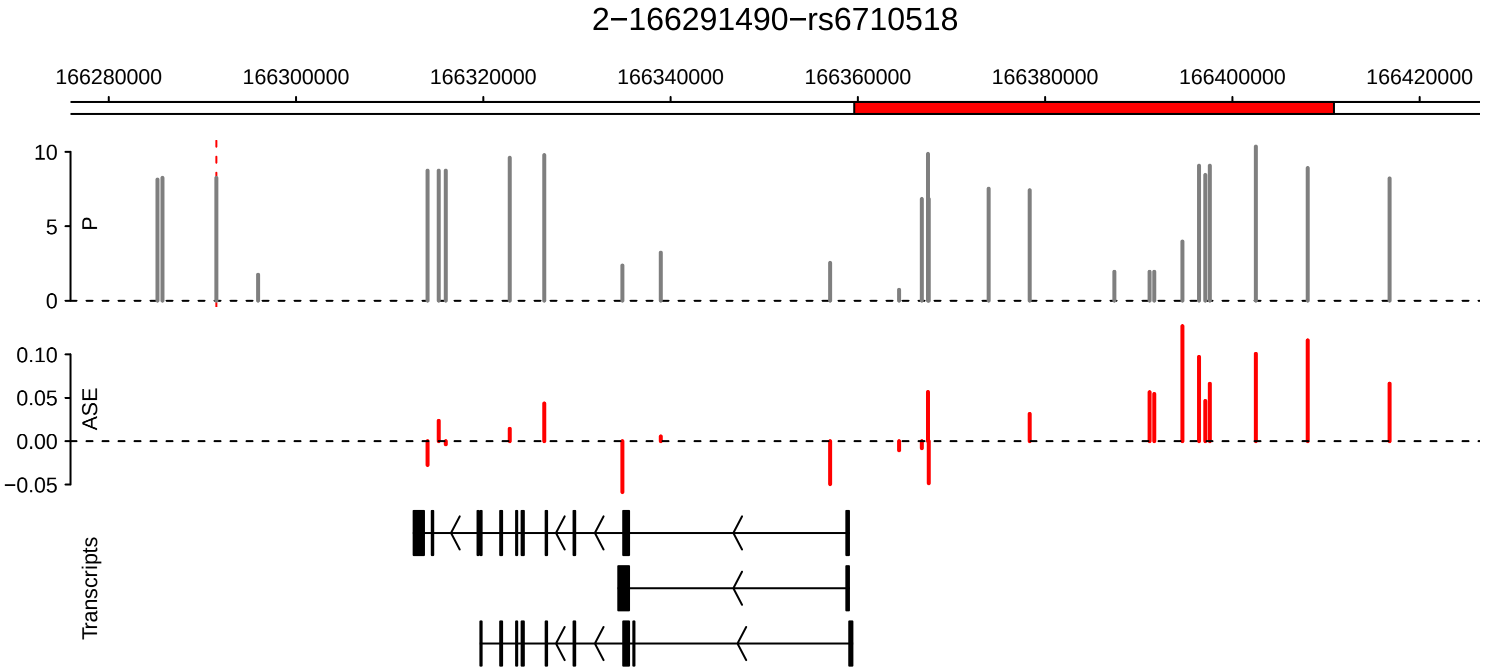
2−166291490−rs6710518
166280000
166300000
166320000
166340000
166360000
166380000
166400000
166420000
10
5
0
P
0.10
0.05
0.00
−0.05
ASE
Transcripts

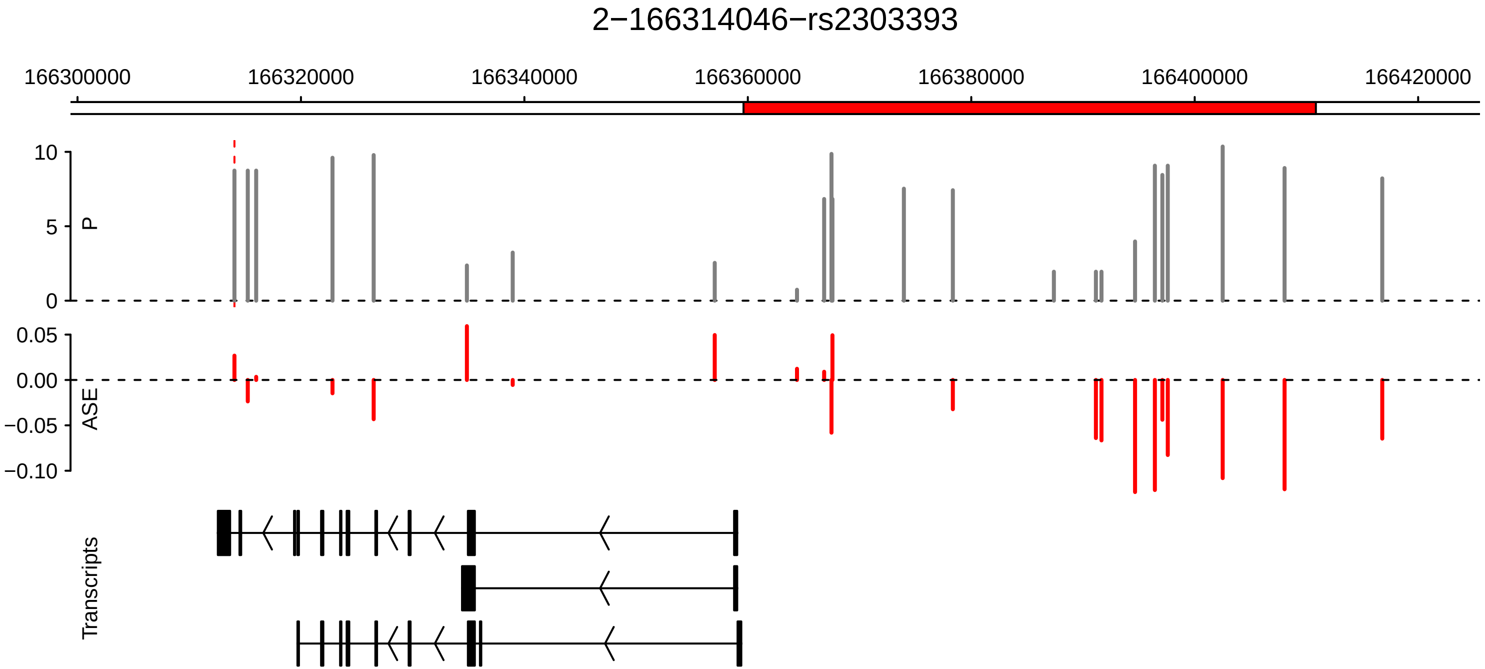
2−166314046−rs2303393
166300000
166320000
166340000
166360000
166380000
166400000
166420000
10
5
0
P
0.05
0.00
−0.05
−0.10
ASE
Transcripts

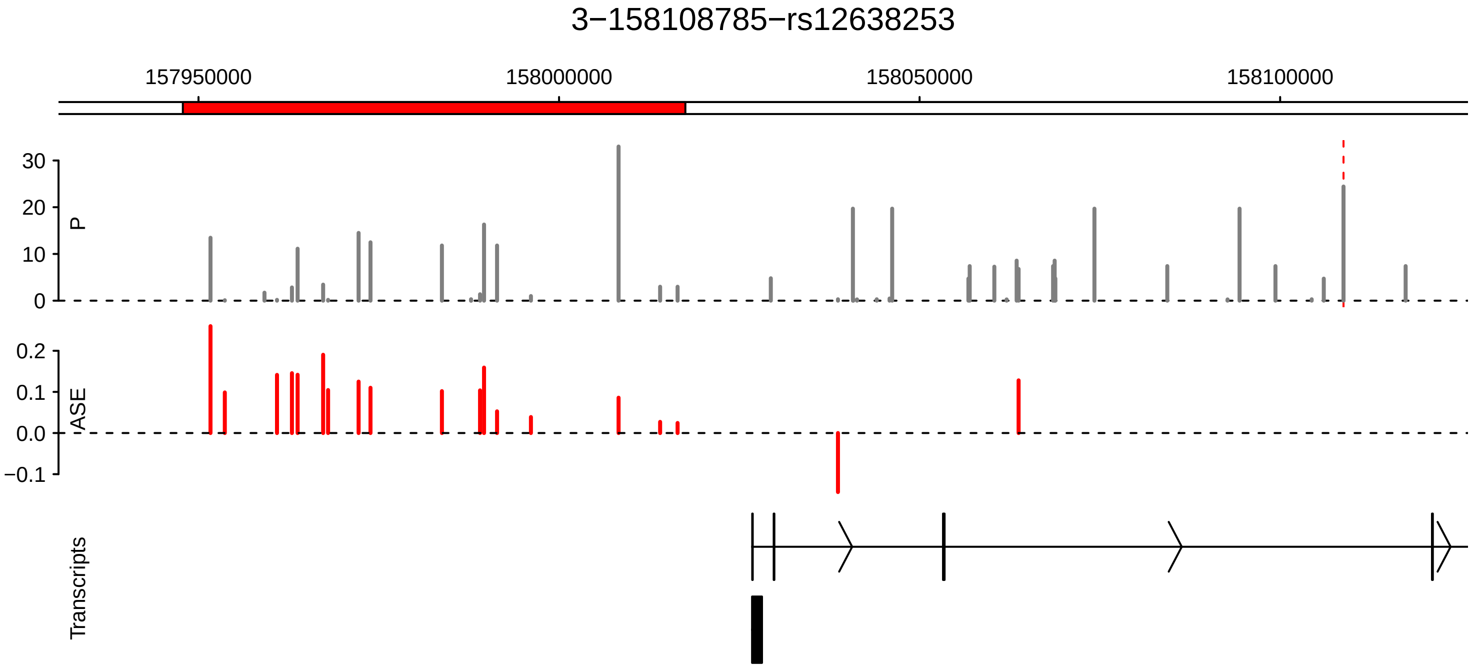

3−158108785−rs12638253
157950000
158000000
158050000
158100000
P
30
20
10
0
ASE
0.2
0.1
0.0
−0.1
Transcripts

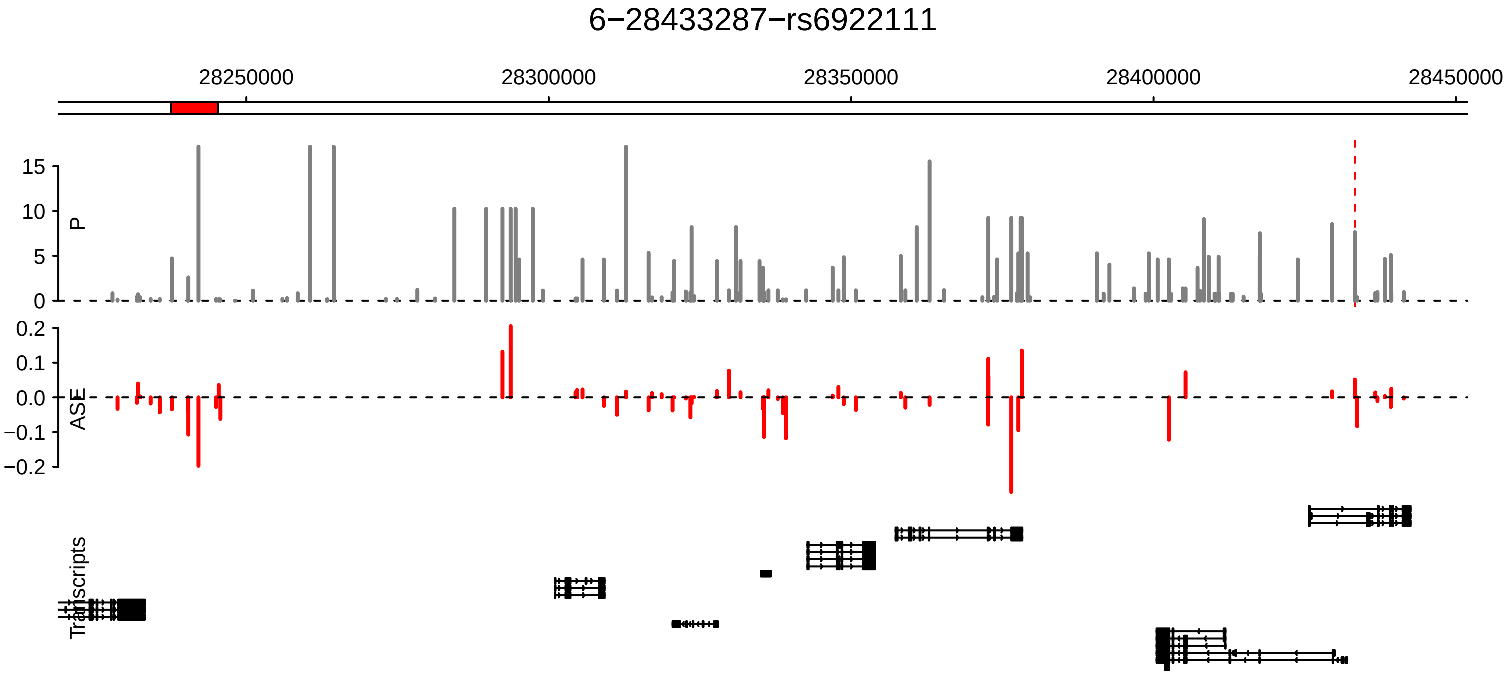

6−28433287−rs6922111
28250000
28300000
28350000
28400000
28450000
P
15
10
5
0
ASE
0.2
0.1
0.0
−0.1
−0.2
Transcripts

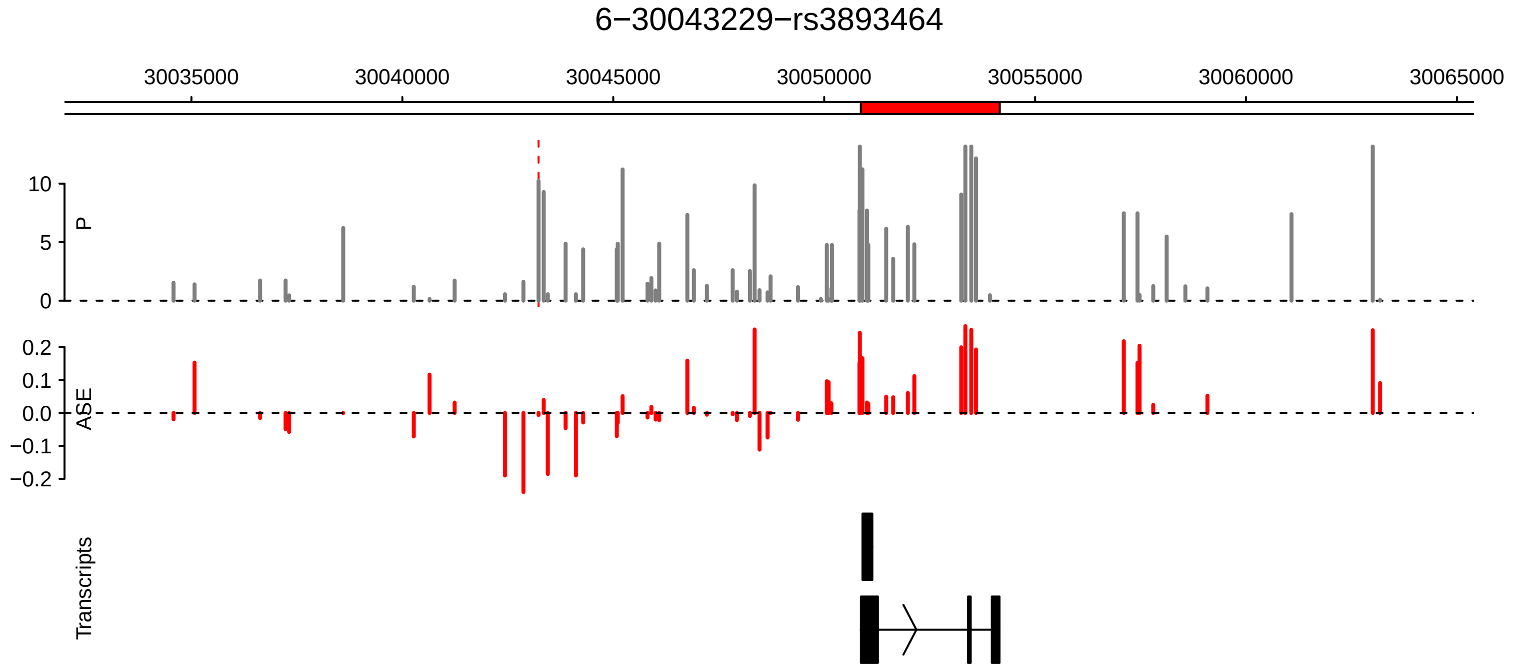
6−30043229−rs3893464
30035000
30040000
30045000
30050000
30055000
30060000
30065000
P
10
5
0
ASE
0.2
0.1
0.0
−0.1
−0.2
Transcripts

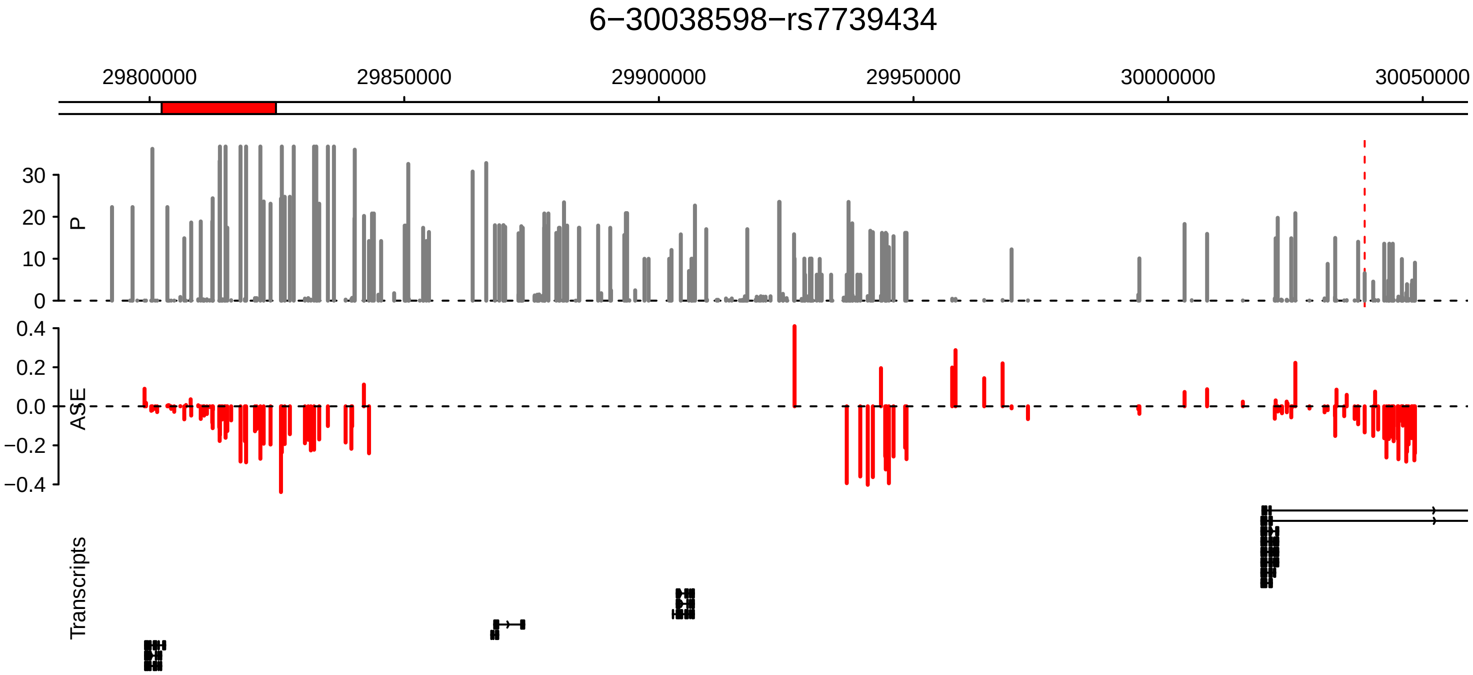

6−30038598−rs7739434
29800000
29850000
29900000
29950000
30000000
30050000
P
30
20
10
0
ASE
0.4
0.2
0.0
−0.2
−0.4
Transcripts

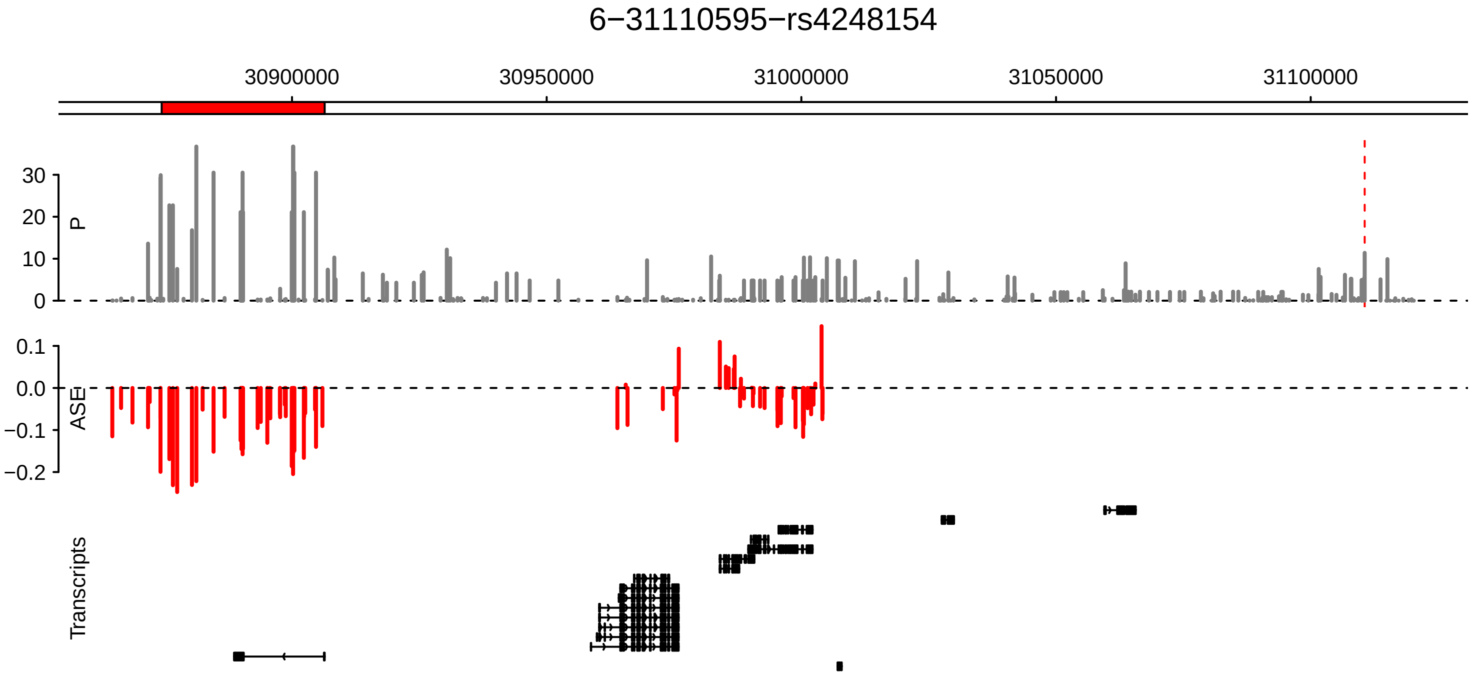
6−31110595−rs4248154
30900000
30950000
31000000
31050000
31100000
P
30
20
10
0
ASE
0.1
0.0
−0.1
−0.2
Transcripts

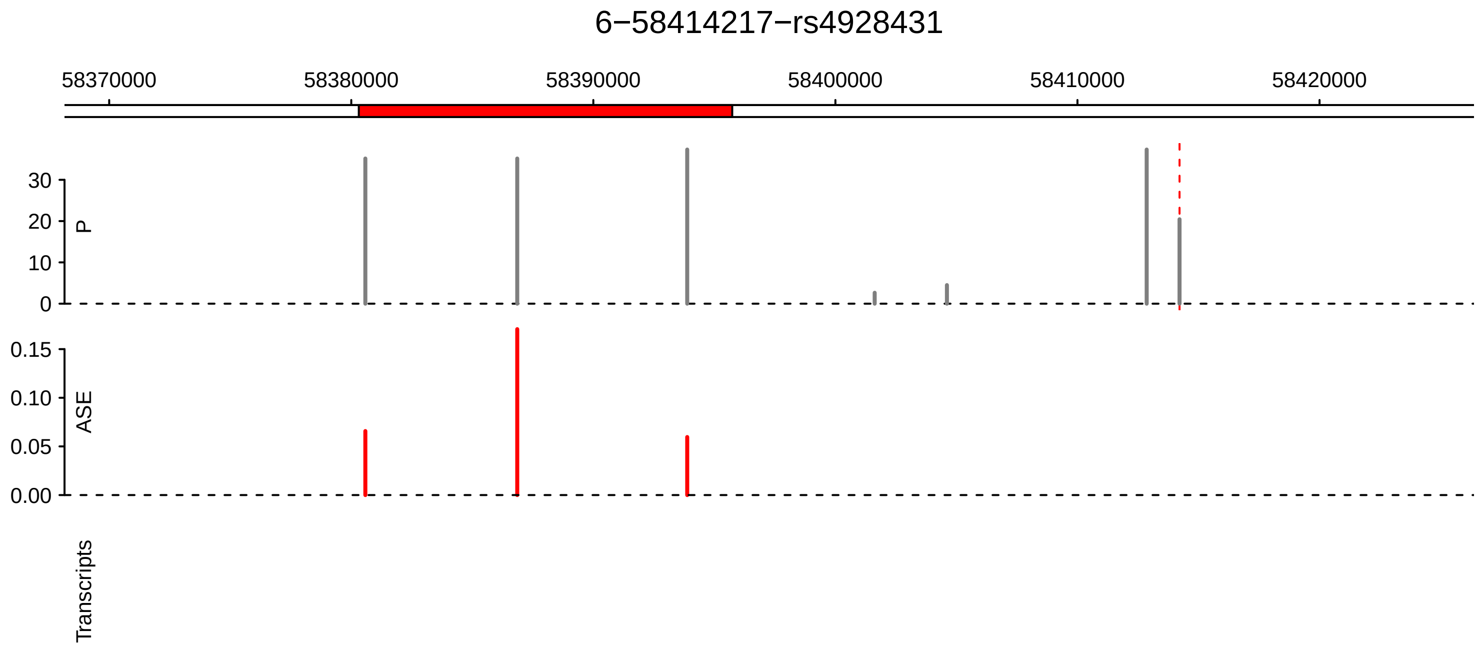

6−58414217−rs4928431
58370000
58380000
58390000
58400000
58410000
58420000
30
20
10
0
P
0.15
0.10
0.05
0.00
ASE
Transcripts

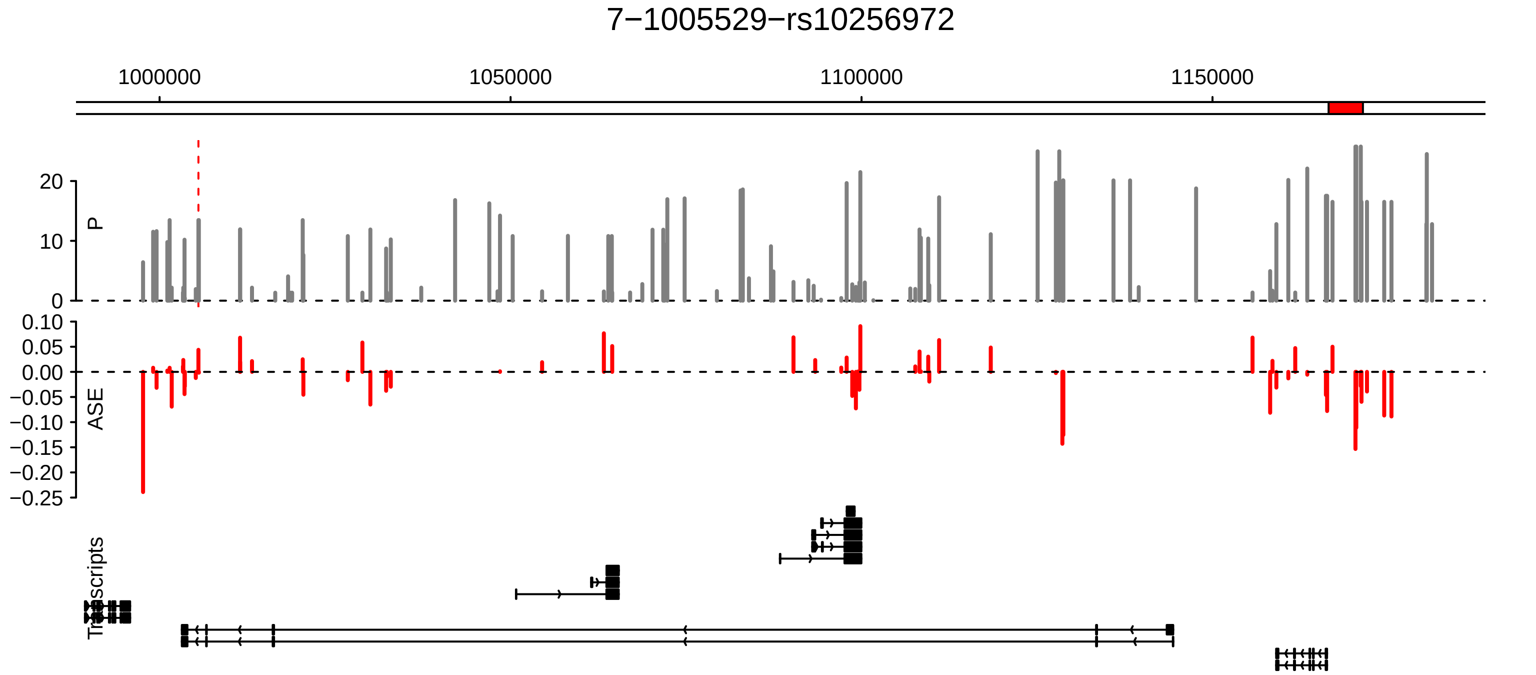
7−1005529−rs10256972
1000000
1050000
1100000
1150000
P
20
10
0
ASE
0.10
0.05
0.00
−0.05
−0.10
−0.15
−0.20
−0.25
Transcripts

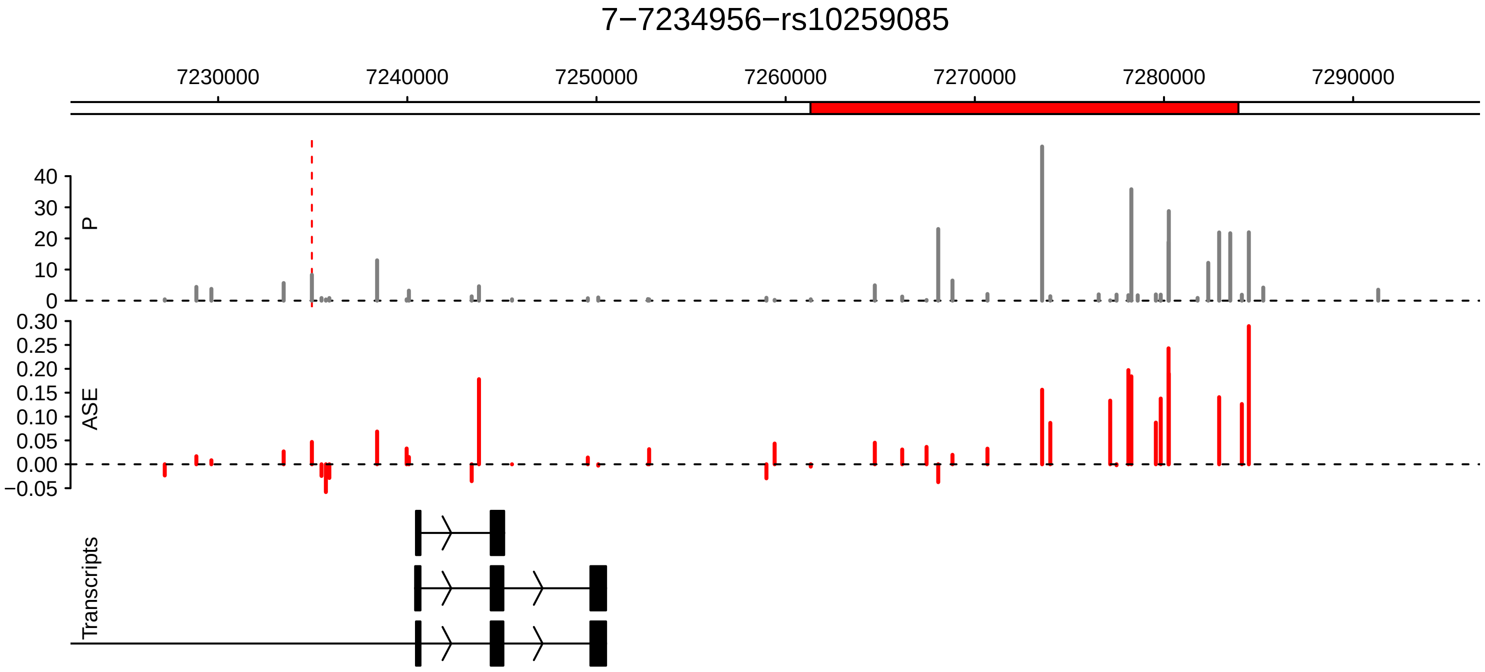
7−7234956−rs10259085
7230000
7240000
7250000
7260000
7270000
7280000
7290000
P
0
10
20
30
40
ASE
−0.05
0.00
0.05
0.10
0.15
0.20
0.25
0.30
Transcripts

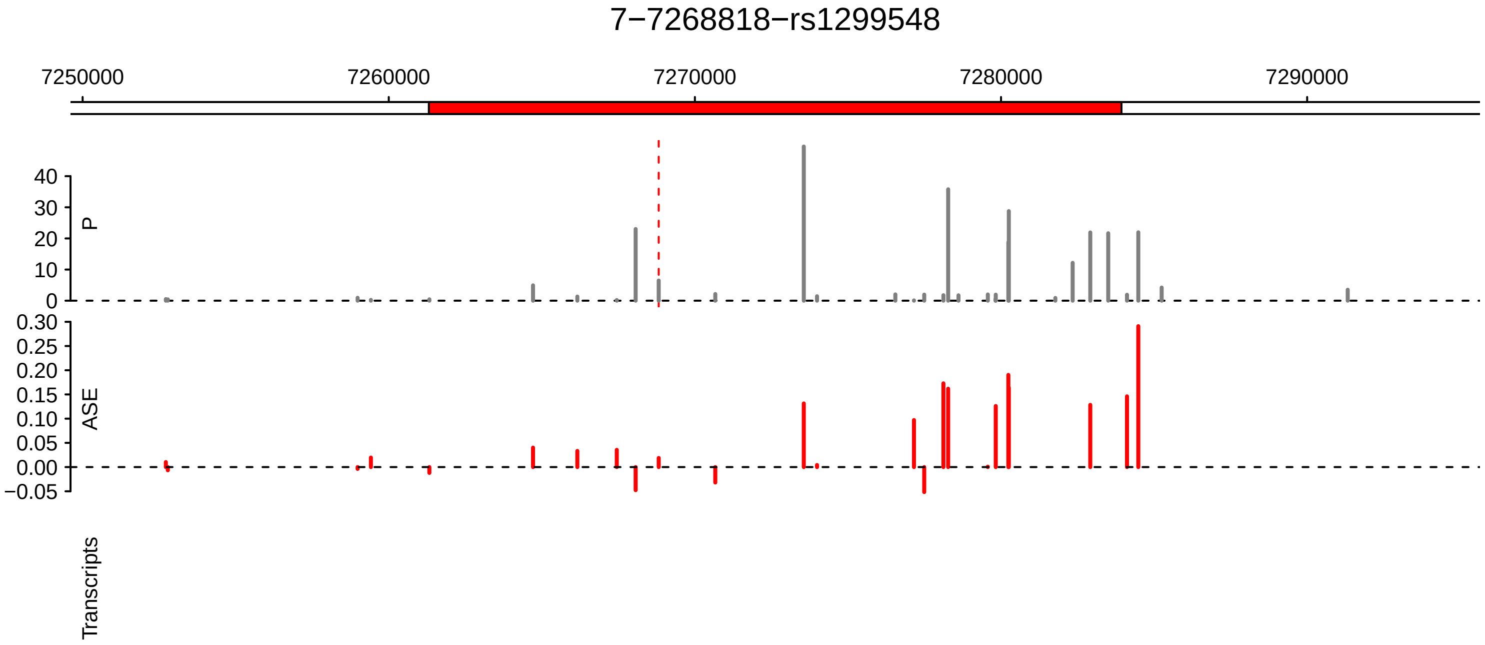

7−7268818−rs1299548
7250000
7260000
7270000
7280000
7290000
P
40
30
20
10
0
ASE
0.30
0.25
0.20
0.15
0.10
0.05
0.00
−0.05
Transcripts

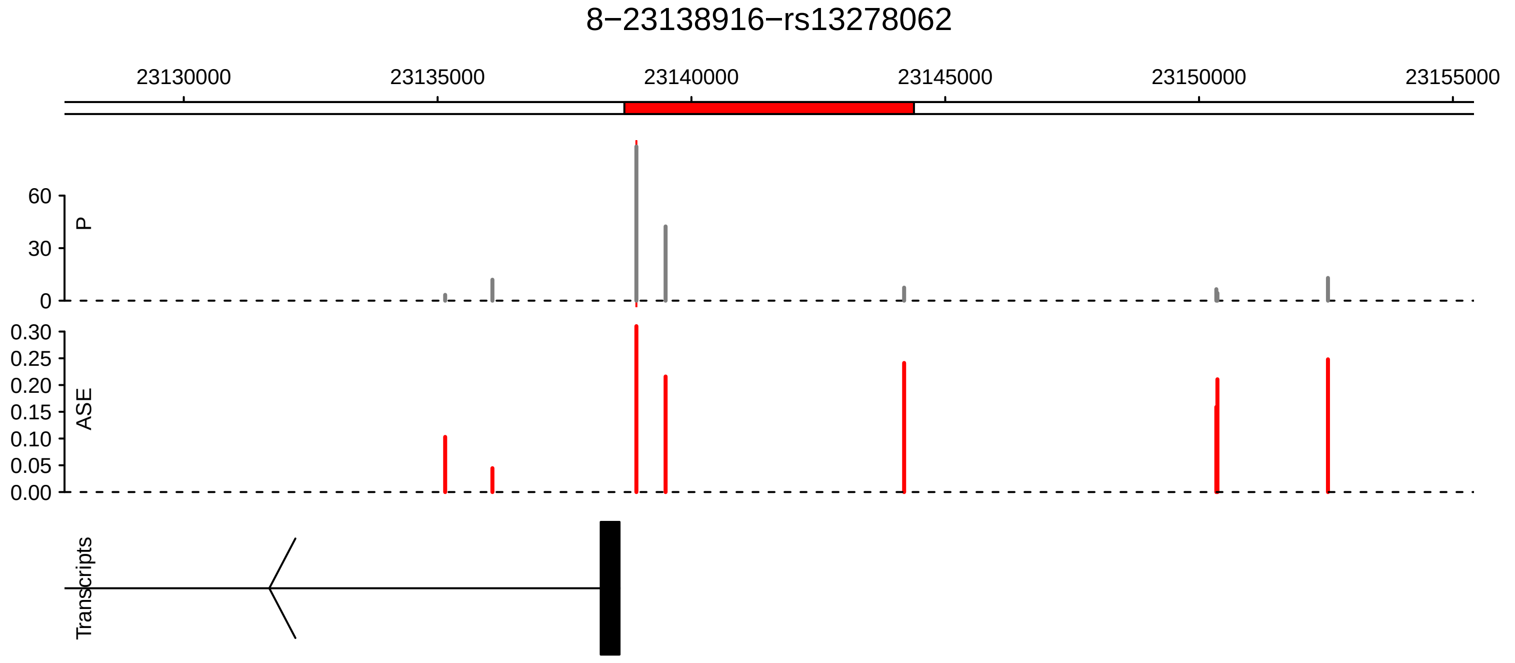
8−23138916−rs13278062
23130000
23135000
23140000
23145000
23150000
23155000
60
30
0
P
0.30
0.25
0.20
0.15
0.10
0.05
0.00
ASE
Transcripts

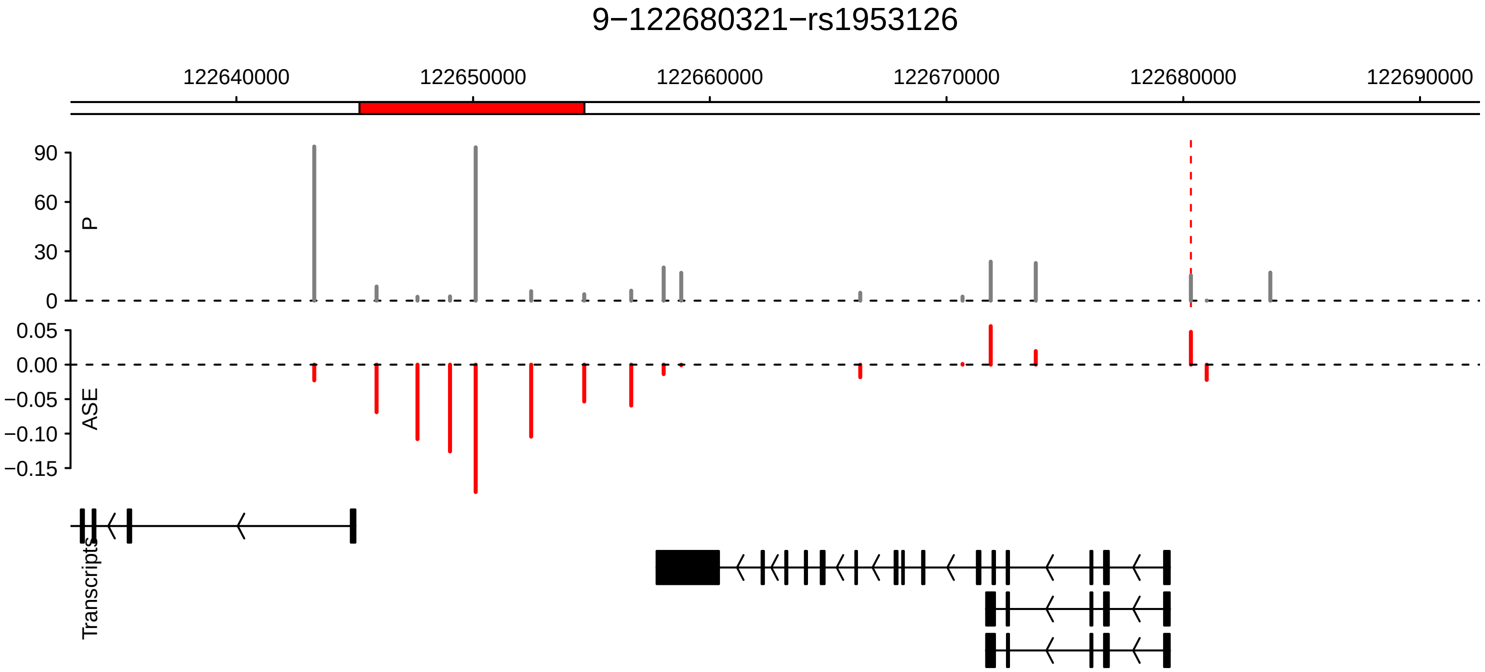

9−122680321−rs1953126
122640000
122650000
122660000
122670000
122680000
122690000
90
60
30
0
P
0.05
0.00
−0.05
−0.10
−0.15
ASE
Transcripts

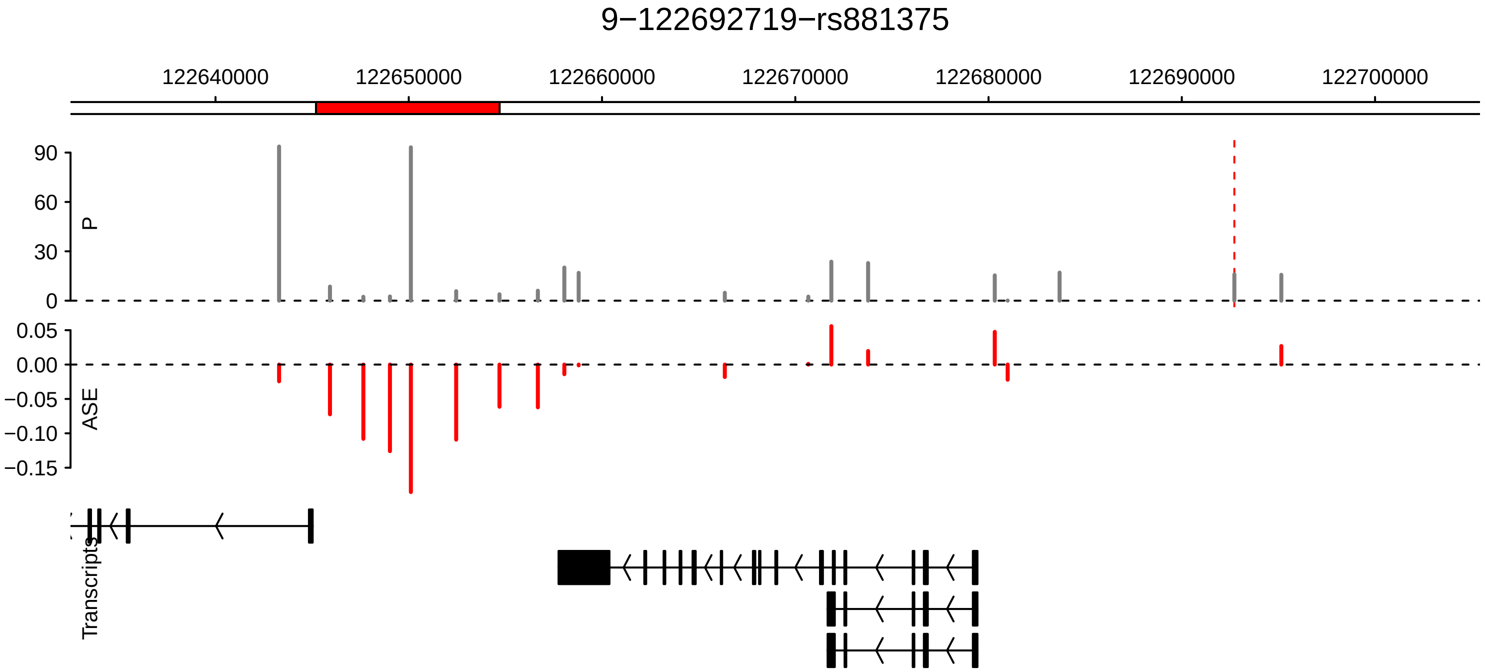

9−122692719−rs881375
122640000
122650000
122660000
122670000
122680000
122690000
122700000
90
60
30
0
P
0.05
0.00
−0.05
−0.10
−0.15
ASE
Transcripts

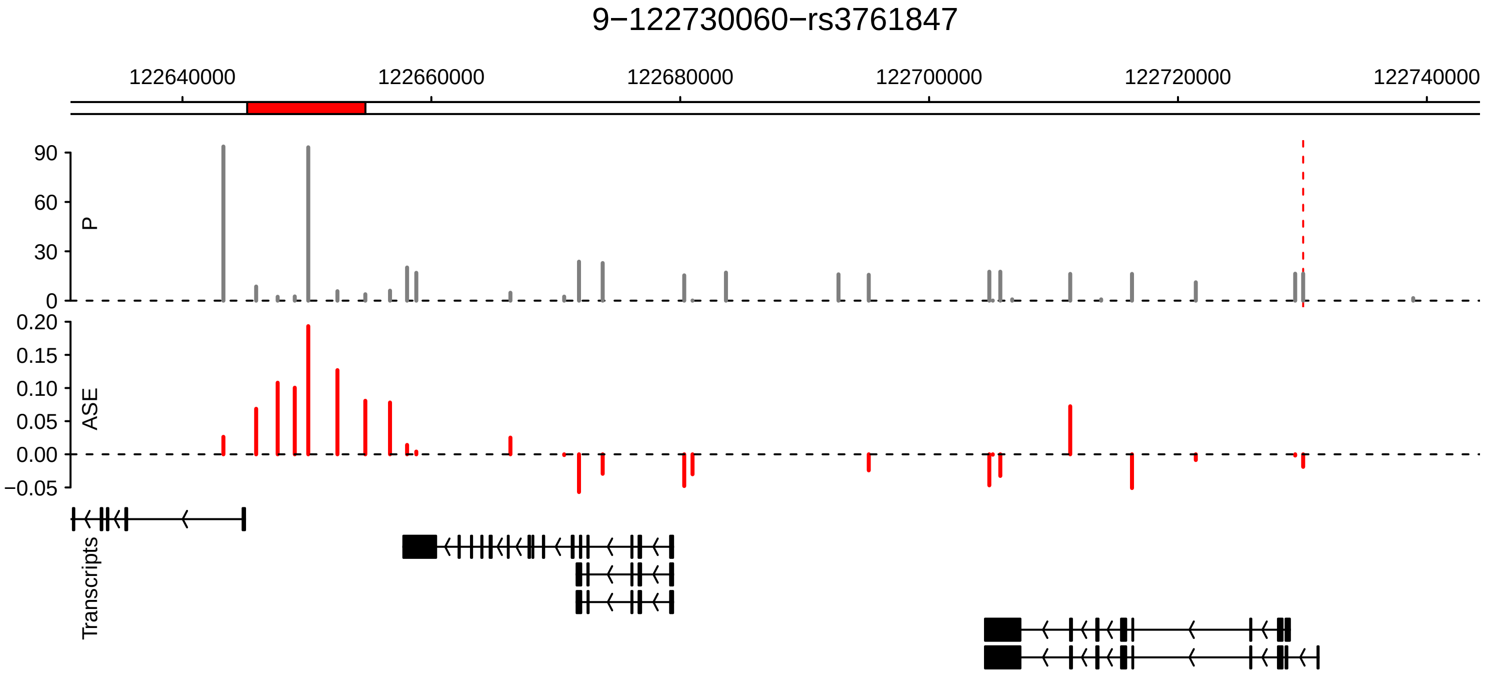
9−122730060−rs3761847
122640000
122660000
122680000
122700000
122720000
122740000
90
60
30
0
P
0.20
0.15
0.10
0.05
0.00
−0.05
ASE
Transcripts

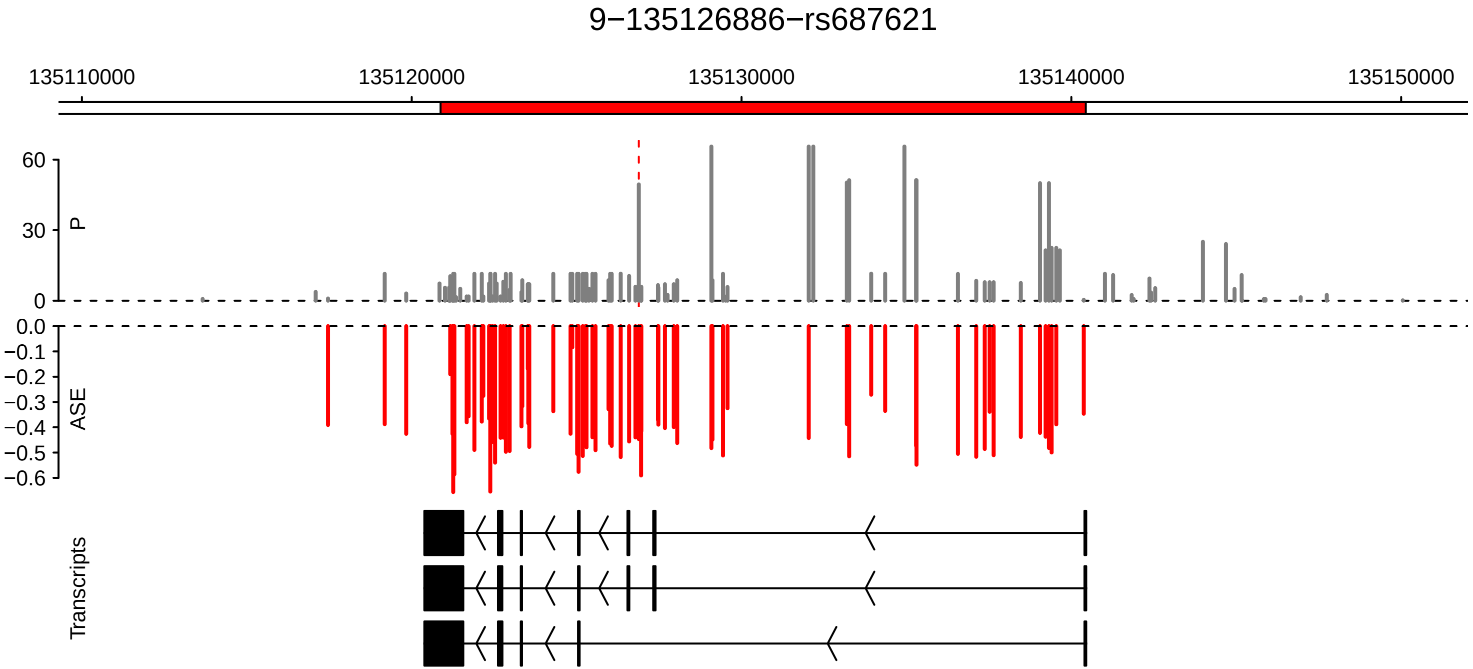
9−135126886−rs687621
135110000
135120000
135130000
135140000
135150000
60
30
0
P
0.0
−0.1
−0.2
−0.3
−0.4
−0.5
−0.6
ASE
Transcripts

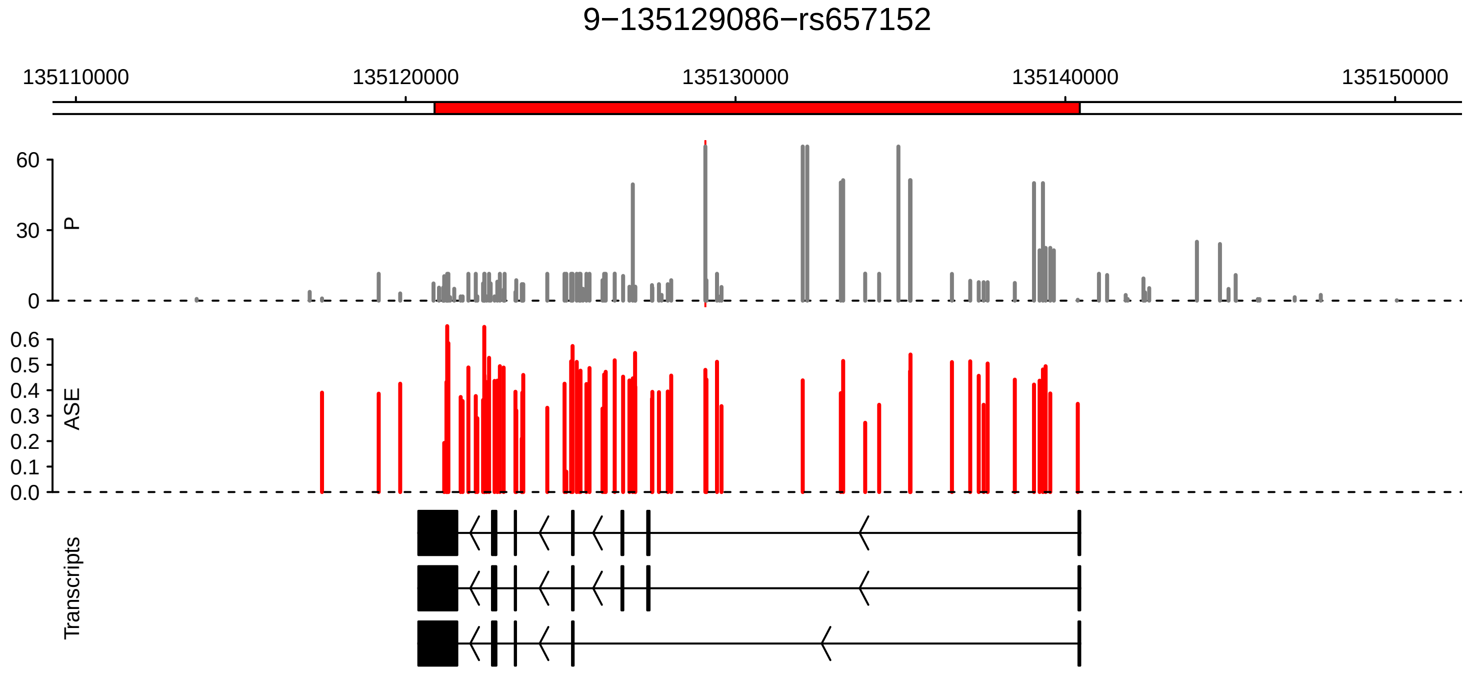
9−135129086−rs657152
135110000
135120000
135130000
135140000
135150000
P
60
30
0
ASE
0.6
0.5
0.4
0.3
0.2
0.1
0.0
Transcripts

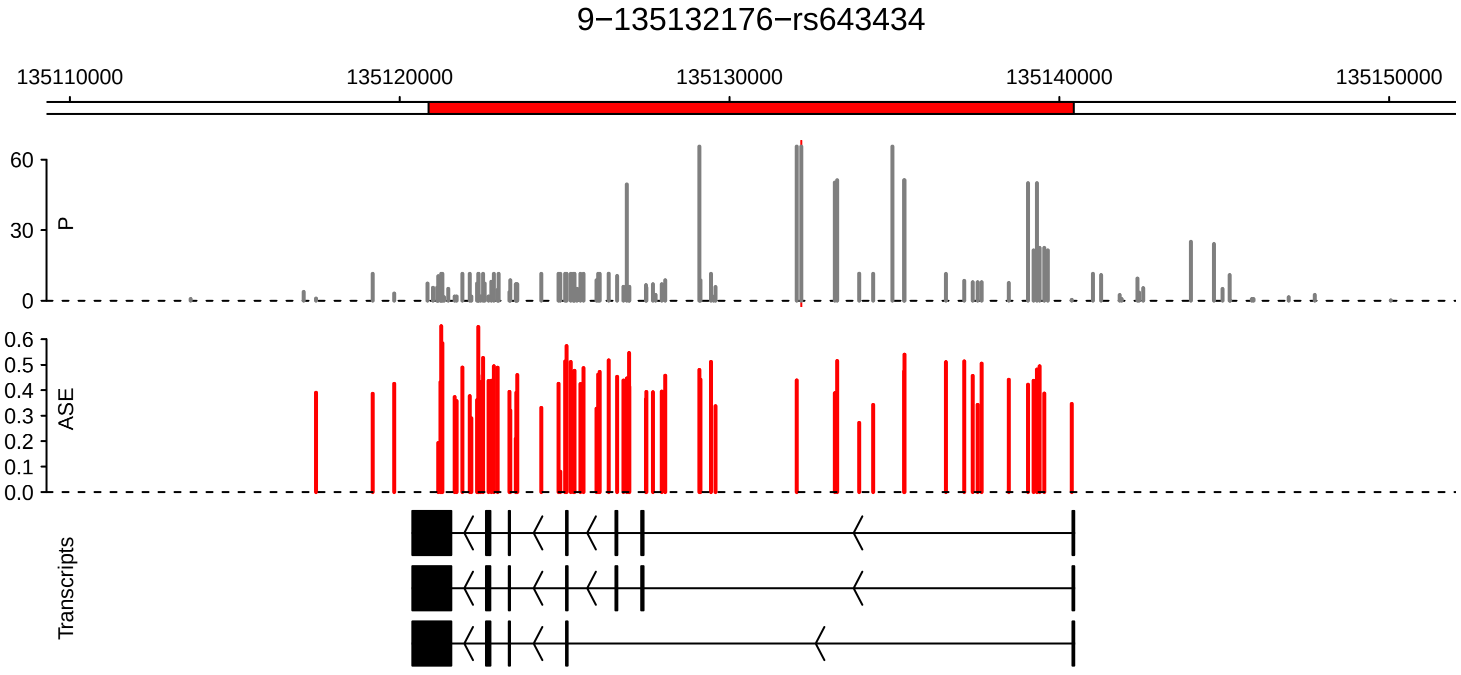
9−135132176−rs643434
135110000
135120000
135130000
135140000
135150000
60
30
0
P
0.6
0.5
0.4
0.3
0.2
0.1
0.0
ASE
Transcripts

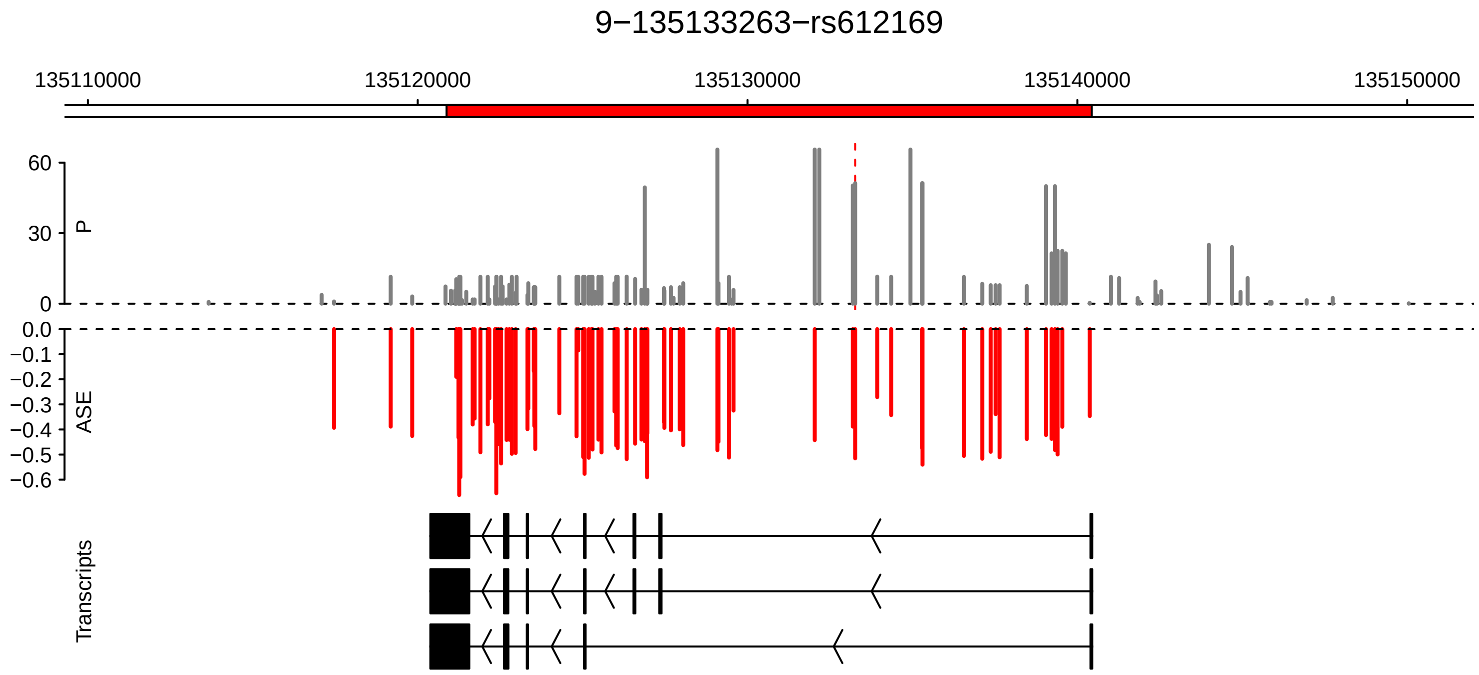
9−135133263−rs612169
135110000
135120000
135130000
135140000
135150000
60
30
0
P
0.0
−0.1
−0.2
−0.3
−0.4
−0.5
−0.6
ASE
Transcripts

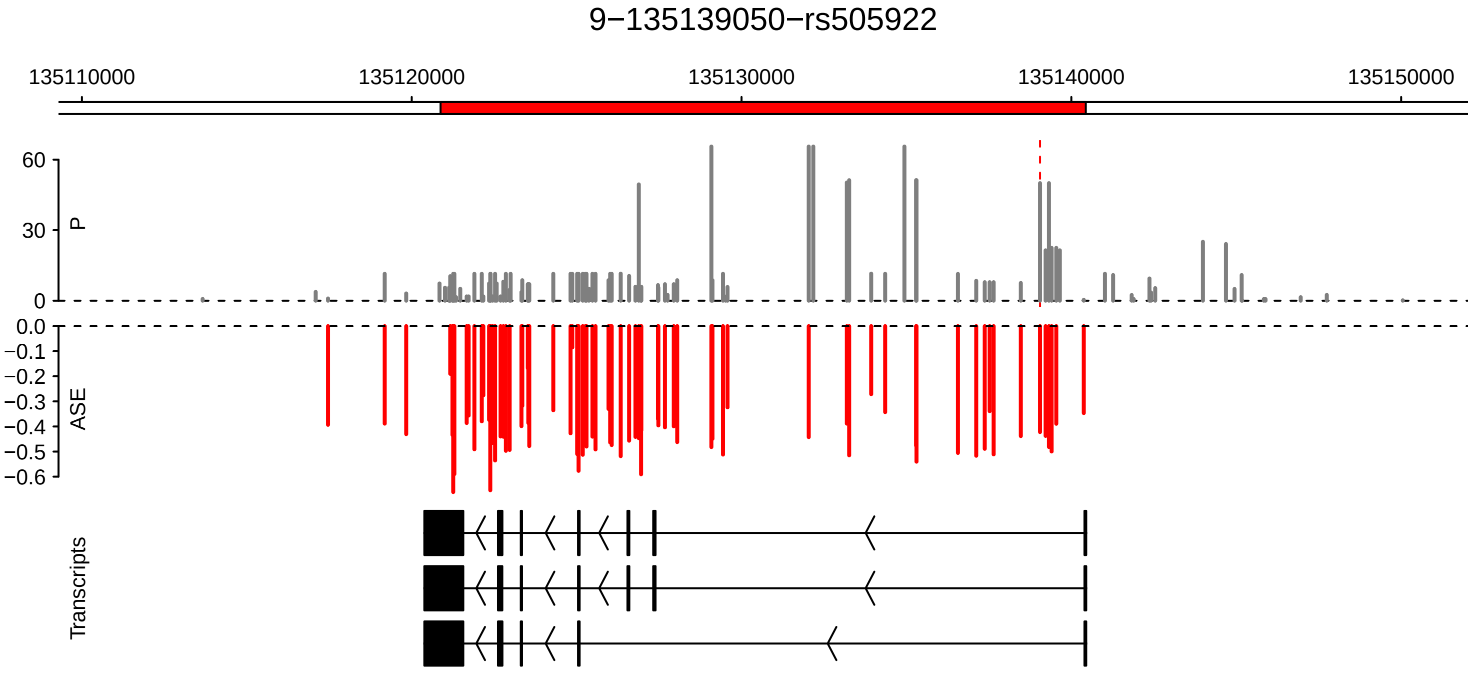

9−135139050−rs505922
135110000
135120000
135130000
135140000
135150000
60
30
0
P
0.0
−0.1
−0.2
−0.3
−0.4
−0.5
−0.6
ASE
Transcripts

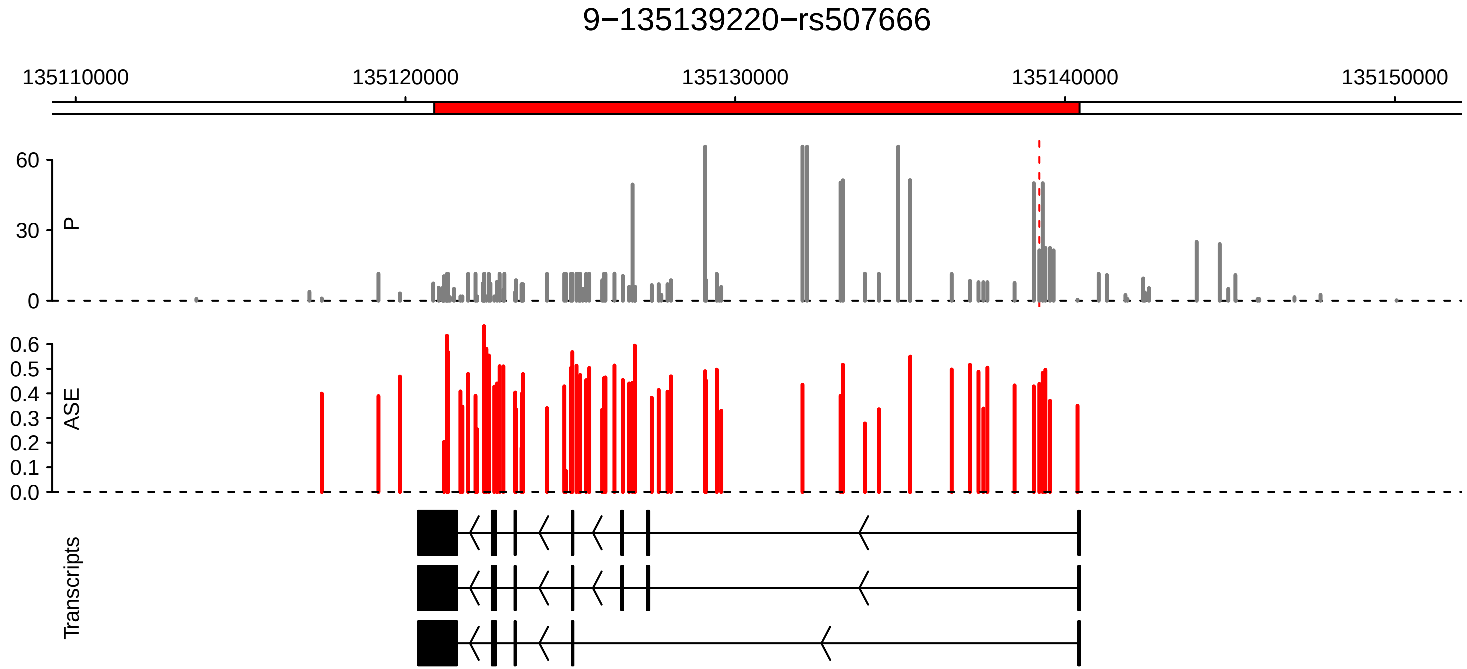

9−135139220−rs507666
135110000
135120000
135130000
135140000
135150000
60
30
0
P
0.6
0.5
0.4
0.3
0.2
0.1
0.0
ASE
Transcripts

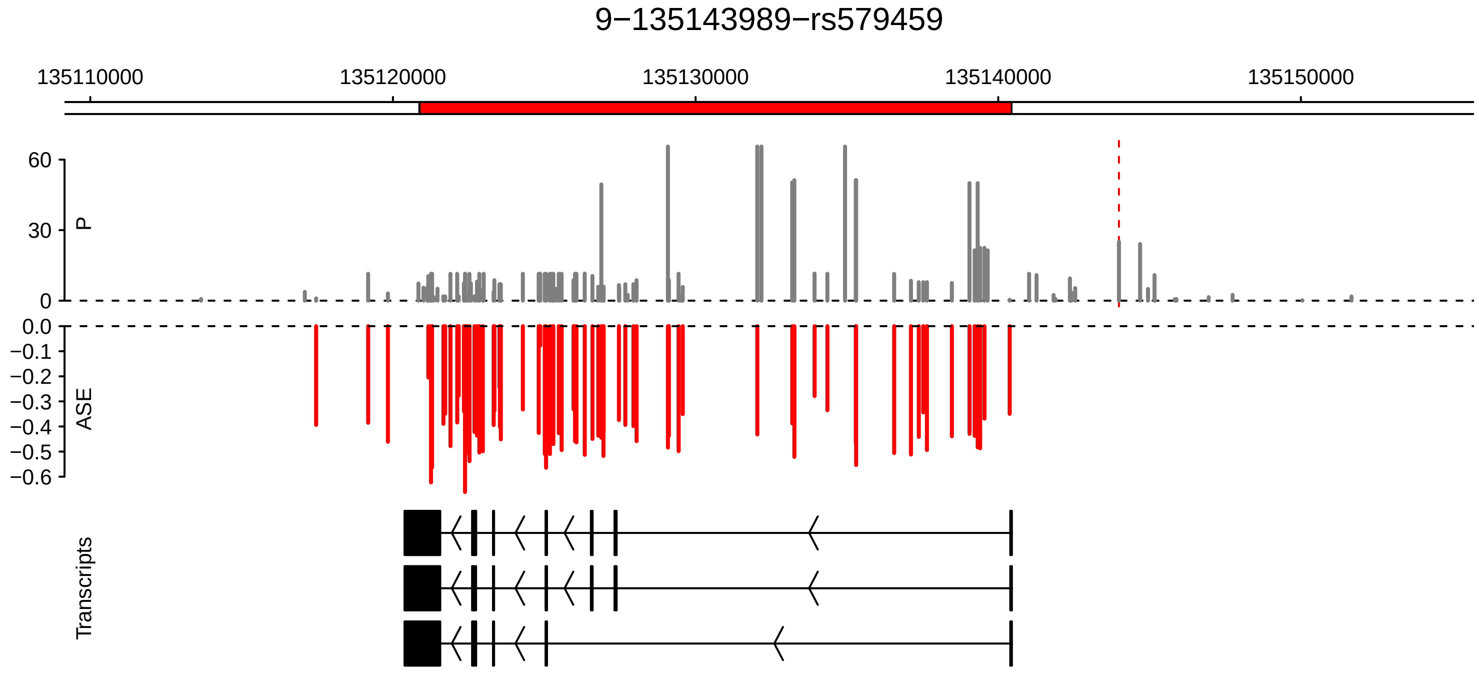
9−135143989−rs579459
135110000
135120000
135130000
135140000
135150000
60
30
0
P
0.0
−0.1
−0.2
−0.3
−0.4
−0.5
−0.6
ASE
Transcripts

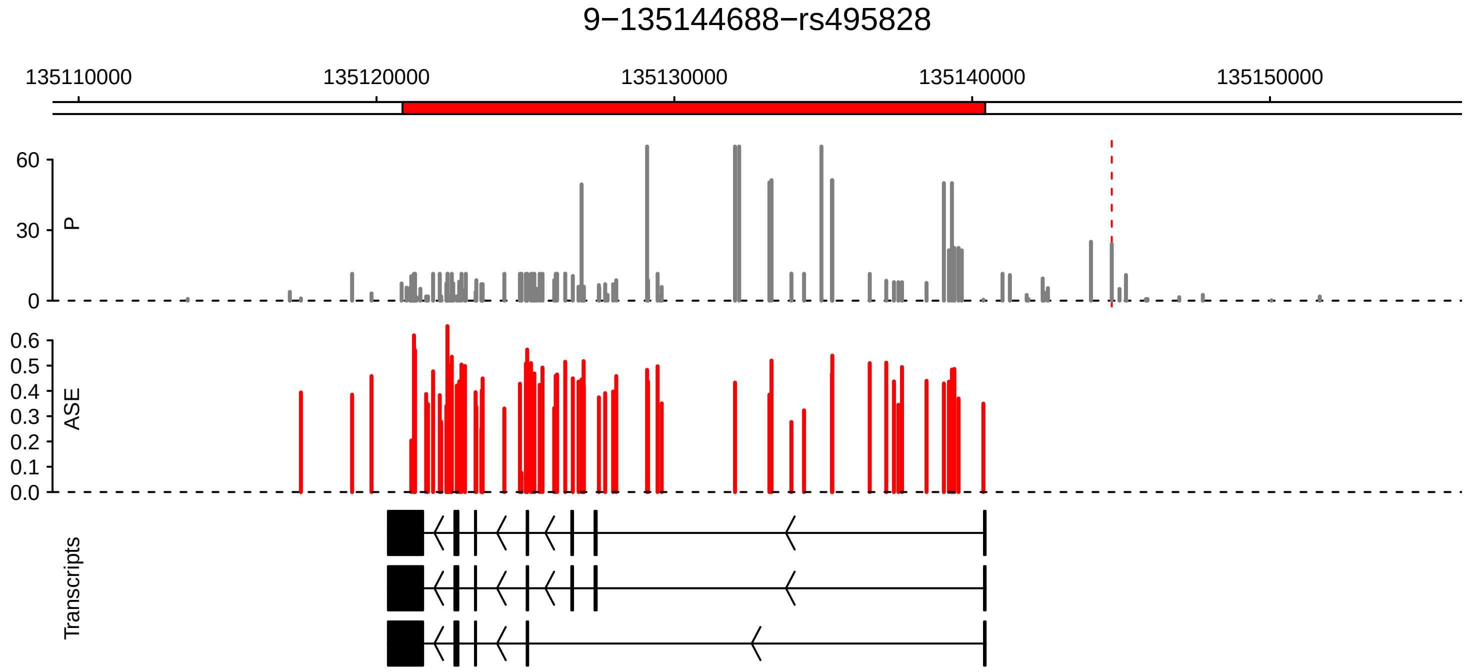
9−135144688−rs495828
135110000
135120000
135130000
135140000
135150000
60
30
0
P
0.6
0.5
0.4
0.3
0.2
0.1
0.0
ASE
Transcripts

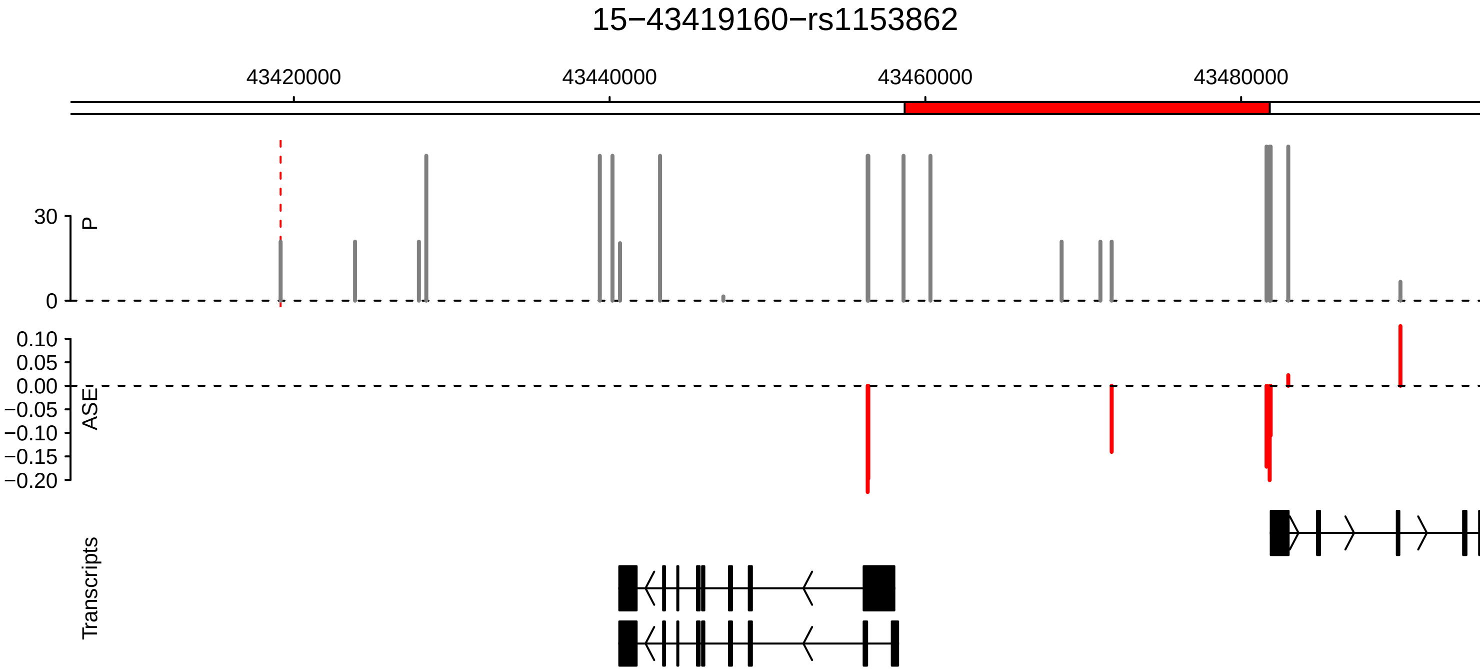

15−43419160−rs1153862
43420000
43440000
43460000
43480000
30
0
P
0.10
0.05
0.00
−0.05
−0.10
−0.15
−0.20
ASE
Transcripts

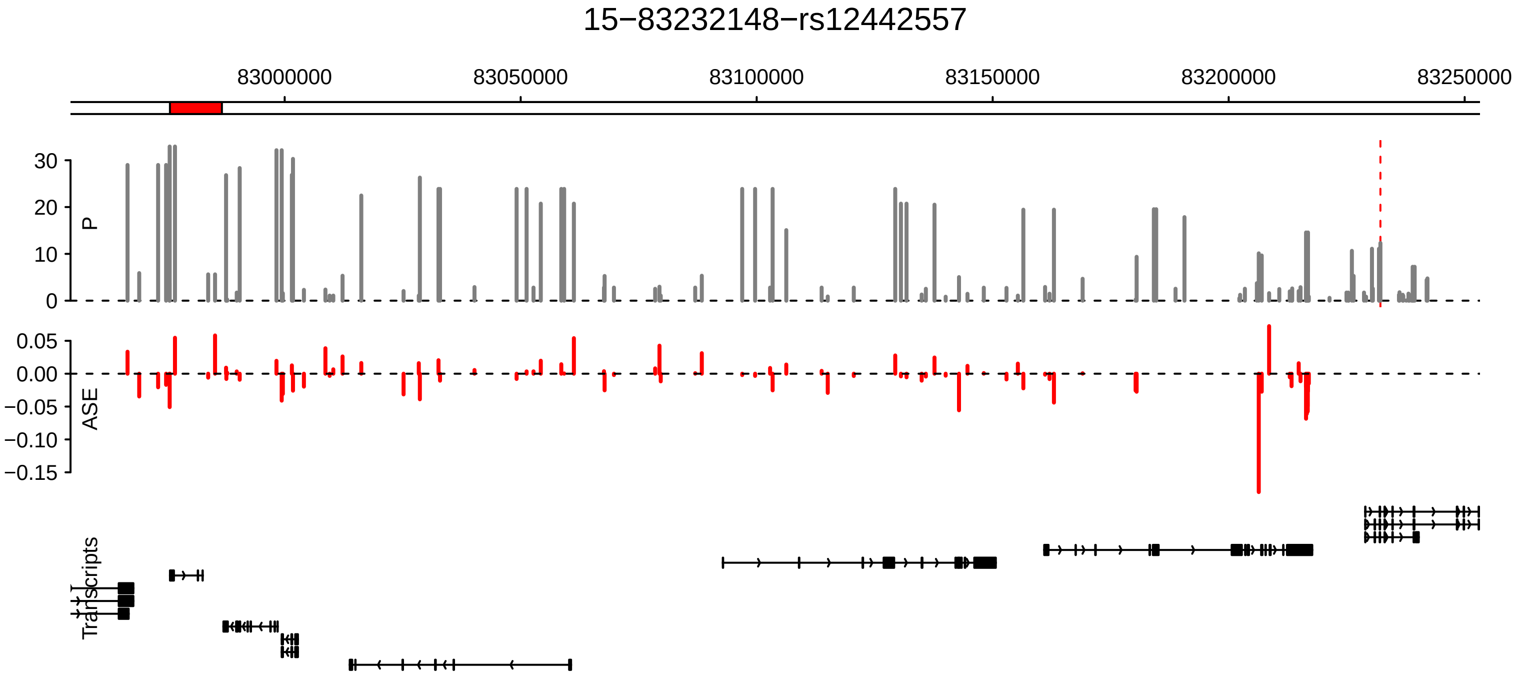

15−83232148−rs12442557
83000000
83050000
83100000
83150000
83200000
83250000
P
30
20
10
0
ASE
0.05
0.00
−0.05
−0.10
−0.15
Transcripts

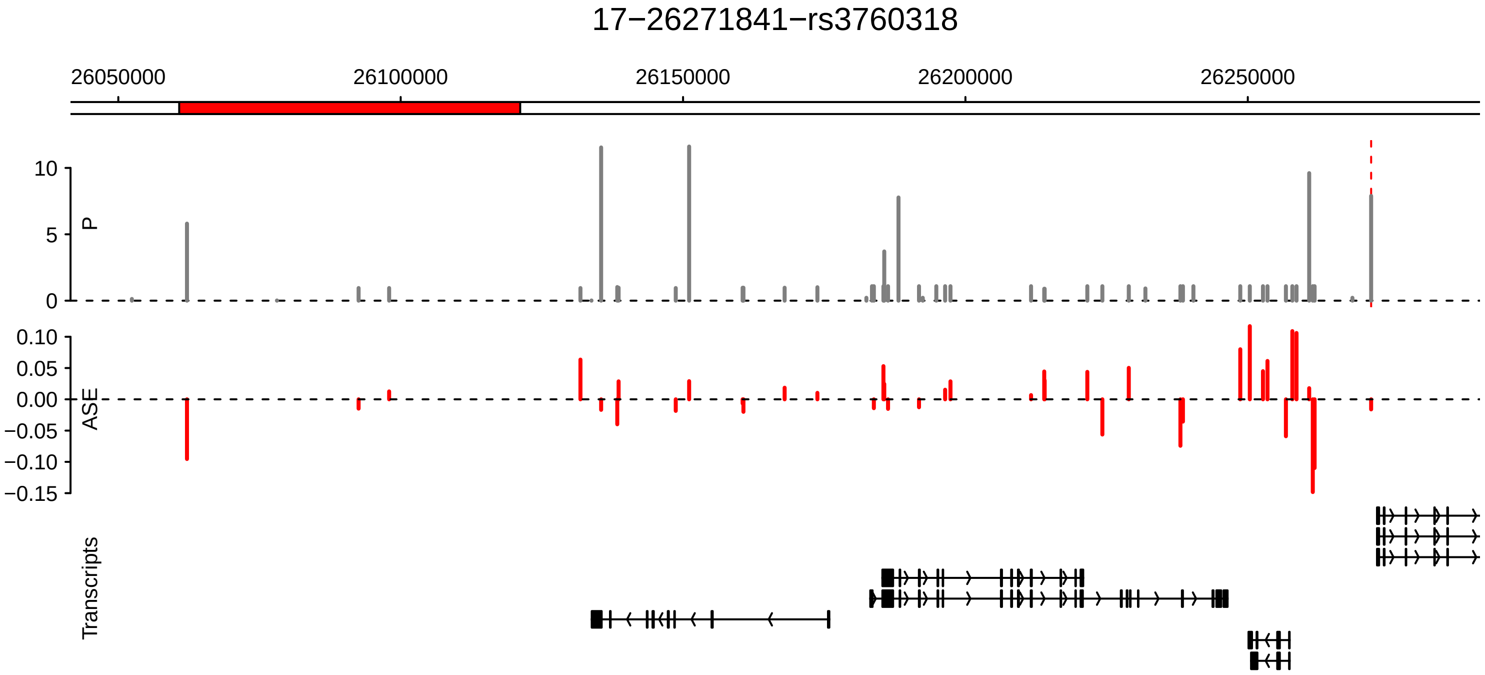
17−26271841−rs3760318
26050000
26100000
26150000
26200000
26250000
P
10
5
0
ASE
0.10
0.05
0.00
−0.05
−0.10
−0.15
Transcripts
