# Supplementary material for: Single Nucleotide Polymorphisms with Cis-Regulatory Effects on Long Non-Coding Transcripts in Human Primary Monocytes
Source: PLoS One. 2014 Jul 15;9(7):e102612. doi: 10.1371/journal.pone.0102612 (PMC4099216; doi:10.1371/journal.pone.0102612)
Supplement: Table S1 — Significantly associated lncRNA regions. (DOCX) [file pone.0102612.s005.docx]

**Table S1.** **Significant lncRNA regions.** lncRNA analysis windows with *cis-*regulatory SNPs associated with allele-specific expression. Only the most strongly associated *cis*-rSNP for each region is shown.

| **lncRNA Region** | **Top-SNP** | **SNP  chromosomal position** | **P-value** | **Slope** | **Average  expression level** |
| --- | --- | --- | --- | --- | --- |
| 1:3806828-3823649 | rs35031681 | 3815571 | 8,44E-11 | 0,059 | 2775 |
| 1:9150914-9185265 | rs12121864 | 9127919 | 7,53E-11 | 0,317 | 1454 |
| 1:22224268-22229983 | GA000431 | 22224627 | 7,24E-48 | -0,124 | 6155 |
| 1:37693067-37712599 | rs6681785 | 37711681 | 4,78E-36 | -0,054 | 3306 |
| 1:41480834-41523120 | rs10524 | 41499060 | 1,17E-47 | -0,259 | 1161 |
| 1:47619187-47646736 | rs2405922 | 47646271 | 1,18E-19 | -0,094 | 2558 |
| 1:59121128-59134925 | rs11207319 | 59119737 | 5,22E-18 | 0,060 | 1172 |
| 1:101263997-101325407 | rs17123757 | 101395548 | 7,96E-13 | 0,054 | 2220 |
| 1:108415627-108418664 | rs3905234 | 108446227 | 1,33E-31 | 0,392 | 10239 |
| 1:113355832-113417250 | rs2798686 | 113402968 | 4,45E-30 | -0,098 | 2007 |
| 1:119484876-119620119 | rs10923755 | 119474867 | 2,87E-34 | -0,102 | 3081 |
| 1:174113102-174116227 | rs10913112 | 174180451 | 6,25E-19 | 0,100 | 3192 |
| 1:174140521-174156272 | rs3748618 | 174384842 | 1,84E-20 | 0,065 | 5400 |
| 1:178065367-178071882 | rs17370290 | 178032795 | 1,52E-10 | 0,068 | 2143 |
| 1:178162399-178177554 | rs3737813 | 178096635 | 3,38E-10 | 0,068 | 986 |
| 1:180685904-180796316 | rs672527 | 180811844 | 1,06E-18 | 0,174 | 427 |
| 1:199754454-199766225 | rs12134525 | 199724837 | 3,62E-07 | 0,051 | 2998 |
| 1:209622768-209672501 | rs1361031 | 209711705 | 2,97E-13 | 0,120 | 1471 |
| 1:220067631-220080631 | rs11118924 | 220234733 | 1,76E-37 | -0,275 | 5965 |
| 1:220153613-220218488 | rs11118924 | 220234733 | 2,15E-65 | -0,254 | 3419 |
| 1:233162036-233172432 | rs2244398 | 233163795 | 8,80E-13 | -0,056 | 4675 |
| 1:242147327-242277242 | rs2047137 | 242263033 | 1,99E-21 | -0,085 | 685 |
| 2:769837-854112 | rs4854402 | 840892 | 1,02E-13 | 0,264 | 447 |
| 2:6886462-6898046 | rs10495545 | 6907471 | 7,38E-25 | 0,186 | 6430 |
| 2:8724217-8735915 | rs1434123 | 8735254 | 4,96E-10 | -0,107 | 2952 |
| 2:19067188-19369296 | rs1876040 | 18998229 | 1,36E-07 | -0,057 | 1201 |
| 2:19932116-19948289 | rs6748924 | 19976702 | 4,60E-10 | 0,091 | 10855 |
| 2:40000337-40185965 | rs6727351 | 40189380 | 1,36E-21 | -0,093 | 1121 |
| 2:47273048-47425632 | rs12612767 | 47302513 | 1,51E-17 | 0,287 | 455 |
| 2:64418705-64422285 | rs17739675 | 64348417 | 5,71E-17 | 0,254 | 2180 |
| 2:64475858-64534436 | rs17739675 | 64348417 | 1,14E-10 | 0,188 | 1120 |
| 2:69780828-69794483 | rs7596535 | 69939465 | 1,21E-09 | -0,138 | 3529 |
| 2:71083169-71145381 | rs10167593 | 71139382 | 5,13E-43 | 0,203 | 6112 |
| 2:74998783-75013429 | rs8179569 | 75001937 | 2,35E-11 | 0,183 | 9093 |
| 2:75020893-75023305 | rs12366 | 75039364 | 5,44E-91 | 0,447 | 5647 |
| 2:85895764-85906025 | rs11127 | 85778240 | 3,59E-08 | -0,151 | 1534 |
| 2:97652641-97685951 | rs1550995 | 97638378 | 1,33E-27 | 0,067 | 3697 |
| 2:101944950-101970061 | rs1859716 | 101939910 | 1,92E-55 | -0,156 | 7933 |
| 2:114453616-114481349 | rs12711793 | 114439272 | 1,50E-25 | -0,108 | 5808 |
| 2:145142022-145550766 | rs17741930 | 145078625 | 2,91E-18 | -0,088 | 706 |
| 2:160235465-160277187 | rs7556888 | 160248507 | 1,94E-31 | -0,053 | 1900 |
| 2:166359607-166374766 | rs1560594 | 166408044 | 4,81E-28 | -0,063 | 3623 |
| 2:166359613-166410847 | rs2304002 | 166422341 | 2,72E-34 | 0,097 | 2556 |
| 2:174453839-174472402 | rs4325816 | 174517145 | 6,99E-67 | 0,199 | 3671 |
| 2:207746603-207795759 | rs6435367 | 207804849 | 2,78E-29 | 0,098 | 2242 |
| 2:207759686-207808852 | rs6435367 | 207804849 | 9,23E-34 | 0,094 | 2330 |
| 2:216119166-216286863 | rs6744457 | 216258500 | 1,85E-10 | -0,171 | 432 |
| 2:216184531-216416504 | rs4674035 | 216402306 | 1,10E-14 | -0,159 | 602 |
| 2:231263880-231273488 | rs6729885 | 231264517 | 3,59E-14 | 0,071 | 3109 |
| 2:242561507-242568100 | rs6737791 | 242566876 | 4,67E-22 | -0,107 | 2018 |
| 3:7971326-8518284 | rs17784233 | 8545390 | 3,12E-19 | 0,223 | 469 |
| 3:10023102-10027779 | rs3732968 | 9988273 | 1,39E-26 | 0,116 | 3905 |
| 3:142886637-142922605 | rs17787766 | 142910105 | 5,05E-09 | -0,189 | 650 |
| 3:144324894-144333425 | rs251483 | 144185774 | 9,28E-12 | -0,059 | 9334 |
| 3:152071522-152094779 | rs628383 | 152092615 | 2,20E-14 | 0,090 | 884 |
| 3:153318652-153352203 | rs650824 | 153299560 | 5,27E-22 | 0,054 | 2128 |
| 3:157947829-158017517 | rs1430410 | 158008256 | 1,02E-33 | -0,096 | 3475 |
| 3:158363935-158369616 | rs7647643 | 158368031 | 2,91E-31 | 0,065 | 2400 |
| 3:173761385-173796091 | rs231988 | 173759673 | 2,50E-22 | -0,110 | 3156 |
| 3:195157855-195204142 | rs6777985 | 195188997 | 6,00E-15 | 0,148 | 2628 |
| 3:195181097-195206581 | rs6777985 | 195188997 | 3,05E-22 | 0,161 | 3697 |
| 4:114420-147779 | rs17802159 | 101487 | 3,48E-59 | 0,290 | 3966 |
| 4:4374590-4386810 | rs1031095 | 4384319 | 1,07E-12 | -0,057 | 1693 |
| 4:6723353-6726458 | rs2285864 | 6721216 | 3,13E-90 | -0,336 | 4210 |
| 4:8534897-8561822 | rs13129077 | 8509294 | 6,33E-17 | 0,079 | 2135 |
| 4:13722690-13750774 | rs3846396 | 13732450 | 1,14E-10 | 0,106 | 1039 |
| 4:13745658-13754314 | rs7670043 | 13747513 | 2,53E-10 | -0,074 | 911 |
| 4:13775177-13853535 | rs3096592 | 13775073 | 1,67E-55 | 0,222 | 5481 |
| 4:15837384-15868908 | rs28516589 | 15843209 | 2,31E-13 | -0,053 | 8806 |
| 4:31642917-31800926 | rs7676427 | 31632356 | 1,09E-32 | -0,343 | 477 |
| 4:35684640-35724051 | rs13139479 | 35742738 | 1,51E-14 | 0,126 | 1285 |
| 4:38044930-38063775 | rs1487630 | 38012218 | 7,35E-20 | -0,219 | 1119 |
| 4:67965618-68016564 | rs1056787 | 68062665 | 9,89E-25 | -0,152 | 6869 |
| 4:74343790-74402534 | rs34740973 | 74352707 | 1,17E-27 | -0,169 | 3327 |
| 4:80111926-80448722 | rs1383429 | 80130514 | 1,86E-08 | 0,162 | 454 |
| 4:82742975-83184421 | rs535707 | 82936570 | 1,38E-21 | -0,274 | 541 |
| 4:84373689-84387390 | rs7658518 | 84379303 | 1,68E-39 | -0,056 | 10670 |
| 4:89425118-89471597 | rs17013978 | 89422221 | 2,67E-08 | -0,078 | 505 |
| 4:95815419-95842744 | rs6532503 | 95827046 | 1,65E-38 | 0,198 | 883 |
| 4:109678795-109761002 | rs11097999 | 109772330 | 5,12E-68 | -0,136 | 1236 |
| 4:144073798-144325464 | rs7666785 | 144279914 | 3,24E-57 | 0,118 | 696 |
| 4:178603127-178835274 | rs2271101 | 178596713 | 4,79E-23 | 0,063 | 1026 |
| 4:185498903-185512124 | rs793903 | 185491125 | 4,51E-16 | 0,106 | 2505 |
| 4:186002733-186013899 | rs7700133 | 186010805 | 5,04E-18 | -0,070 | 3432 |
| 5:6363554-6390405 | rs4592 | 6425248 | 2,68E-13 | 0,161 | 782 |
| 5:6739438-6760824 | rs1651071 | 6729931 | 2,48E-24 | 0,189 | 1290 |
| 5:6848993-6879841 | rs1004758 | 6845985 | 2,67E-17 | 0,095 | 803 |
| 5:17183137-17270156 | rs2731792 | 17264545 | 8,44E-07 | -0,055 | 484 |
| 5:17497119-17537055 | rs2459822 | 17530812 | 4,22E-16 | -0,062 | 1255 |
| 5:33459888-33476482 | rs3756663 | 33476063 | 6,90E-105 | -0,433 | 3344 |
| 5:53652531-53746714 | rs35939 | 53642897 | 1,36E-07 | -0,053 | 733 |
| 5:59819724-59879241 | rs7712091 | 59859548 | 2,14E-39 | -0,108 | 4386 |
| 5:77673945-77691973 | rs6860842 | 77775106 | 7,25E-19 | -0,107 | 3095 |
| 5:87600489-87768258 | rs4916908 | 87714808 | 1,30E-28 | -0,053 | 2083 |
| 5:98292775-98358617 | rs331934 | 98167620 | 1,54E-24 | 0,051 | 3105 |
| 5:108600720-108689969 | rs246105 | 108700845 | 5,12E-24 | 0,161 | 1743 |
| 5:118375397-118434484 | rs299216 | 118424127 | 1,62E-33 | -0,154 | 1043 |
| 5:119609042-119697096 | rs1348478 | 119462092 | 9,64E-14 | -0,217 | 529 |
| 5:127304017-127446691 | rs1112956 | 127461697 | 2,56E-70 | -0,121 | 2763 |
| 5:135493095-135498478 | rs10463951 | 135602010 | 6,44E-14 | 0,053 | 3320 |
| 5:139044414-139104779 | rs165193 | 139068261 | 7,39E-07 | -0,050 | 2887 |
| 5:139517088-139528554 | rs269782 | 139525486 | 2,11E-32 | -0,092 | 2689 |
| 5:159827853-159847011 | rs17057846 | 159834891 | 9,95E-25 | -0,089 | 3773 |
| 5:169551161-169558723 | rs12189473 | 169560982 | 5,49E-07 | 0,138 | 889 |
| 5:172939252-172944677 | rs258868 | 172960799 | 2,28E-10 | 0,072 | 3909 |
| 5:173150418-173168648 | rs10056714 | 173137664 | 8,88E-12 | 0,102 | 868 |
| 5:176970860-176979361 | rs901380 | 176962013 | 5,70E-16 | 0,109 | 10329 |
| 5:177299229-177342661 | rs11743574 | 177331142 | 8,93E-18 | 0,200 | 4141 |
| 6:2799890-2821743 | rs2252076 | 2821580 | 1,71E-20 | -0,182 | 5703 |
| 6:4081305-4102618 | rs13206717 | 4099017 | 3,32E-16 | -0,066 | 1702 |
| 6:6637976-6659207 | rs201036 | 6654117 | 2,59E-16 | -0,066 | 7853 |
| 6:8380925-8657518 | rs1335639 | 8428818 | 1,32E-15 | 0,132 | 523 |
| 6:10851552-10855788 | rs570023 | 10852599 | 6,94E-39 | -0,080 | 4645 |
| 6:14949434-14979938 | rs16875788 | 14972013 | 1,03E-08 | -0,068 | 3607 |
| 6:25353562-25369618 | rs10498723 | 25500324 | 3,71E-07 | -0,065 | 3089 |
| 6:28237538-28245351 | rs10484404 | 28163474 | 1,04E-18 | 0,136 | 3097 |
| 6:30050868-30054162 | rs9258525 | 29914320 | 6,79E-14 | -0,225 | 2579 |
| 6:30842581-30868006 | rs3131043 | 30866445 | 3,64E-33 | -0,136 | 862 |
| 6:30874410-30906415 | rs12192704 | 30900249 | 1,85E-37 | 0,177 | 4979 |
| 6:58380311-58395738 | rs4928431 | 58414217 | 3,60E-21 | -0,075 | 6077 |
| 6:84750166-84773761 | rs9353162 | 84761133 | 3,56E-13 | -0,099 | 4094 |
| 6:88138030-88159238 | rs242268 | 88118603 | 1,60E-14 | -0,076 | 2386 |
| 6:89065388-89187471 | rs1870832 | 89213746 | 1,68E-28 | -0,336 | 1328 |
| 6:96912971-97076266 | rs9372136 | 97057375 | 3,64E-57 | -0,200 | 775 |
| 6:109179552-109197838 | rs9480873 | 109158898 | 2,58E-10 | 0,055 | 1261 |
| 6:129889464-129915425 | rs2571591 | 129904485 | 3,66E-10 | -0,051 | 1276 |
| 6:131190239-131199966 | rs1044303 | 131190430 | 2,26E-30 | -0,104 | 1060 |
| 6:138305909-138308632 | rs10872479 | 138307941 | 3,74E-17 | -0,091 | 4693 |
| 6:158623283-158653378 | rs9355652 | 158701423 | 2,12E-55 | 0,110 | 5169 |
| 7:1166536-1171429 | rs2949192 | 1170367 | 1,70E-26 | -0,078 | 5809 |
| 7:2443924-2454011 | rs11772554 | 2449661 | 8,67E-13 | 0,051 | 4450 |
| 7:7261310-7283935 | rs4724965 | 7273557 | 3,00E-50 | -0,168 | 2109 |
| 7:17285984-17304595 | rs2301677 | 17292583 | 5,49E-14 | -0,068 | 2408 |
| 7:26404876-26505119 | rs1013688 | 26405856 | 1,89E-41 | -0,118 | 1280 |
| 7:29604936-29691396 | rs17603986 | 29657014 | 1,35E-34 | 0,127 | 2047 |
| 7:35761099-35806741 | rs345375 | 35751149 | 4,69E-09 | 0,059 | 2262 |
| 7:38347741-38384763 | rs10240848 | 38373402 | 1,80E-59 | 0,261 | 5759 |
| 7:95063930-95080967 | rs3807892 | 95064669 | 6,19E-59 | -0,221 | 3542 |
| 7:104368889-104390017 | rs7776707 | 104392766 | 1,00E-21 | 0,056 | 5741 |
| 7:135361049-135419956 | rs7808376 | 135355382 | 9,71E-19 | -0,166 | 4538 |
| 7:135428245-135772714 | rs7808376 | 135355382 | 5,52E-08 | -0,106 | 1124 |
| 7:150518893-150533514 | rs122815 | 150571590 | 3,73E-09 | 0,130 | 834 |
| 8:2572265-2667411 | rs6558685 | 2584738 | 8,39E-12 | 0,245 | 1522 |
| 8:6248480-6251477 | rs2440452 | 6252287 | 7,72E-16 | 0,103 | 2927 |
| 8:23138679-23144384 | rs13278062 | 23138916 | 9,45E-89 | -0,270 | 5900 |
| 8:23138691-23144373 | rs13278062 | 23138916 | 9,45E-89 | -0,270 | 5900 |
| 8:58568012-58666672 | rs1868841 | 58526557 | 3,06E-19 | 0,175 | 1254 |
| 8:61459701-61591893 | rs948421 | 61566519 | 6,08E-30 | 0,078 | 636 |
| 8:61985166-62041569 | rs3748648 | 62014020 | 3,70E-40 | 0,290 | 822 |
| 8:90690745-90839090 | rs9297722 | 90864652 | 1,02E-23 | 0,136 | 1860 |
| 8:92141313-92151527 | rs6999873 | 92129841 | 4,14E-26 | -0,208 | 6770 |
| 8:96288411-96297778 | rs7824517 | 96295427 | 1,07E-17 | 0,135 | 1503 |
| 8:126548363-126622797 | rs16900667 | 126596016 | 5,24E-09 | 0,117 | 415 |
| 8:128420701-128474058 | rs10956365 | 128473069 | 1,23E-07 | 0,325 | 1929 |
| 8:130433119-130761667 | rs7827366 | 130500589 | 9,77E-27 | 0,072 | 1019 |
| 8:130497377-130527280 | rs2719194 | 130486553 | 8,09E-24 | -0,096 | 1296 |
| 8:134967921-134983757 | rs16905012 | 134974920 | 1,84E-15 | -0,217 | 1019 |
| 9:3516723-3661624 | rs7864107 | 3555211 | 1,97E-12 | -0,139 | 1875 |
| 9:27235682-27272791 | rs1853186 | 27251030 | 2,61E-11 | 0,128 | 557 |
| 9:70345772-70437996 | rs7046236 | 70374589 | 6,12E-24 | -0,088 | 3214 |
| 9:85024066-85038977 | rs1323771 | 85022947 | 7,67E-10 | 0,076 | 1386 |
| 9:92921165-92984865 | rs10732402 | 92944592 | 2,90E-08 | -0,085 | 510 |
| 9:92921241-92965190 | rs10732402 | 92944592 | 5,24E-07 | -0,089 | 550 |
| 9:95978705-96006639 | rs10993070 | 95950779 | 3,85E-31 | -0,052 | 6193 |
| 9:97560713-97677583 | rs1369156 | 97596715 | 2,43E-53 | 0,139 | 1307 |
| 9:101387889-101621992 | rs7022059 | 101632509 | 3,56E-17 | 0,059 | 414 |
| 9:109222386-109232904 | rs10978863 | 109256242 | 1,62E-10 | -0,189 | 1502 |
| 9:112401576-112407320 | rs4978941 | 112402922 | 1,61E-14 | -0,248 | 675 |
| 9:112941347-113086937 | rs12339649 | 112984867 | 8,54E-14 | -0,137 | 969 |
| 9:114905439-114913574 | rs10981630 | 114906094 | 3,98E-11 | 0,159 | 1348 |
| 9:116458405-116464475 | rs1887784 | 116461032 | 2,50E-22 | -0,057 | 9445 |
| 9:116468537-116473850 | rs10982360 | 116457763 | 3,25E-24 | 0,094 | 2011 |
| 9:116921639-116940509 | rs2989519 | 116884060 | 4,17E-10 | 0,051 | 967 |
| 9:119585547-119670170 | rs7030459 | 119577167 | 3,42E-40 | 0,104 | 4419 |
| 9:119642421-119699355 | rs1927936 | 119579791 | 1,63E-42 | -0,120 | 3608 |
| 9:122645199-122654702 | rs12343516 | 122643290 | 2,11E-94 | 0,192 | 10550 |
| 9:123177340-123254276 | rs2572854 | 123196869 | 9,49E-67 | -0,167 | 2088 |
| 9:131291207-131315768 | rs1220795 | 131293740 | 5,38E-62 | -0,076 | 10114 |
| 9:131304090-131315786 | rs1220795 | 131293740 | 8,16E-22 | -0,065 | 11722 |
| 9:135120874-135140438 | rs644234 | 135132038 | 2,69E-66 | 0,434 | 3316 |
| 9:136968818-136974862 | rs7849296 | 136971025 | 4,64E-31 | -0,130 | 6827 |
| 10:4083918-4121205 | rs17132103 | 4137131 | 1,90E-14 | 0,111 | 1319 |
| 10:5266321-5295165 | rs9423393 | 5276162 | 8,29E-41 | -0,170 | 932 |
| 10:28328723-28378550 | rs7903206 | 28389479 | 5,35E-07 | 0,052 | 1520 |
| 10:28761943-28824748 | rs11007134 | 28840208 | 5,80E-25 | -0,247 | 653 |
| 10:51450948-51497569 | rs4576765 | 51471919 | 4,98E-27 | -0,174 | 1581 |
| 10:91416924-91451157 | rs1326196 | 91561901 | 3,19E-21 | 0,109 | 808 |
| 10:91665226-91707110 | rs1359380 | 91633288 | 3,31E-12 | 0,137 | 965 |
| 10:122512903-122541427 | rs2997312 | 122557692 | 3,13E-26 | 0,133 | 1311 |
| 10:127361802-127388236 | rs7909932 | 127390423 | 7,97E-16 | 0,055 | 914 |
| 11:10843384-10884925 | rs7125450 | 10856012 | 5,48E-12 | 0,050 | 2620 |
| 11:10863008-10877445 | rs7109947 | 10843870 | 3,06E-21 | 0,123 | 1331 |
| 11:15530977-15569220 | rs7113468 | 15497447 | 1,96E-12 | -0,190 | 918 |
| 11:15550145-15600513 | rs729418 | 15572255 | 5,29E-24 | 0,136 | 1192 |
| 11:28330230-28505164 | rs11030292 | 28338175 | 2,76E-07 | -0,060 | 832 |
| 11:60579785-60591583 | rs3862666 | 60628649 | 4,08E-08 | 0,071 | 2433 |
| 11:62376037-62379933 | rs10897294 | 62330454 | 4,49E-31 | 0,079 | 10820 |
| 11:82180352-82205836 | rs4759 | 82213611 | 1,16E-09 | 0,109 | 712 |
| 11:94010852-94013647 | rs4550183 | 94012539 | 3,78E-21 | 0,136 | 8532 |
| 11:94014721-94024442 | rs623404 | 94021777 | 6,08E-20 | -0,157 | 3274 |
| 11:119105503-119115543 | rs4489755 | 119110690 | 1,37E-28 | 0,063 | 3044 |
| 12:8339849-8440666 | rs11044920 | 8411186 | 9,36E-11 | 0,080 | 7327 |
| 12:8364244-8413418 | rs10841346 | 8401135 | 7,14E-20 | -0,110 | 7307 |
| 12:8592224-8611476 | rs11046221 | 8611627 | 4,63E-20 | -0,089 | 7982 |
| 12:9980445-9987361 | rs7313235 | 10023550 | 2,23E-37 | 0,299 | 5208 |
| 12:11256393-11295570 | rs10772397 | 11029950 | 1,04E-51 | -0,267 | 4115 |
| 12:11443582-11530879 | rs7132674 | 11235571 | 2,82E-11 | 0,169 | 1560 |
| 12:13044652-13084880 | rs850942 | 13039758 | 9,33E-20 | -0,119 | 3728 |
| 12:27149351-27205593 | rs841603 | 27202749 | 8,20E-28 | -0,142 | 900 |
| 12:31921280-31931404 | rs1624791 | 31935031 | 1,11E-40 | 0,119 | 6709 |
| 12:38836308-38847681 | rs2638260 | 38835230 | 1,41E-52 | 0,135 | 1245 |
| 12:63187213-63213685 | rs12315614 | 63207224 | 9,22E-07 | -0,053 | 1505 |
| 12:65571923-65749209 | rs7132460 | 65818072 | 2,64E-07 | -0,364 | 387 |
| 12:67013300-67122263 | rs401460 | 67069987 | 1,90E-07 | -0,087 | 1598 |
| 12:91064088-91103691 | rs790463 | 91101680 | 1,76E-19 | -0,055 | 3742 |
| 13:20775651-20820860 | rs7336525 | 20816844 | 3,63E-25 | 0,132 | 4046 |
| 13:26644396-26655135 | rs7998459 | 26587885 | 1,80E-12 | 0,150 | 685 |
| 13:39816094-39822440 | rs2875384 | 39815820 | 2,00E-11 | 0,069 | 1252 |
| 13:51285484-51317287 | rs4290380 | 51287348 | 7,81E-11 | -0,053 | 1700 |
| 13:73760165-73880940 | rs4885169 | 73798794 | 8,44E-17 | 0,175 | 482 |
| 13:78878445-78895091 | rs9574386 | 78732483 | 9,00E-29 | 0,162 | 2001 |
| 14:52754180-53223726 | rs10483621 | 52701283 | 2,11E-16 | -0,055 | 834 |
| 14:76577160-76605599 | rs8022640 | 76594340 | 2,84E-14 | -0,059 | 2418 |
| 14:95069009-95070962 | rs12431494 | 95063139 | 1,82E-47 | -0,081 | 4444 |
| 15:38118804-38146783 | rs8041057 | 38117921 | 1,60E-28 | 0,069 | 6158 |
| 15:43458687-43481812 | rs3809472 | 43481610 | 2,28E-55 | -0,177 | 1083 |
| 15:48919365-48988088 | rs12916255 | 48976999 | 1,11E-30 | 0,091 | 2148 |
| 15:55379855-55387251 | rs2431016 | 55395984 | 9,93E-08 | 0,050 | 5994 |
| 15:82975686-82986699 | rs2292463 | 82976754 | 1,17E-33 | -0,054 | 9436 |
| 17:602650-605326 | rs3813431 | 604565 | 7,96E-08 | 0,082 | 2530 |
| 17:13873334-13913500 | rs11650509 | 13895076 | 5,21E-23 | -0,059 | 4436 |
| 17:26060783-26121168 | rs6505211 | 26151077 | 2,45E-12 | 0,077 | 8464 |
| 17:42487466-42532626 | rs8080126 | 42439278 | 2,37E-58 | -0,143 | 5744 |
| 17:45713937-45720215 | rs2254177 | 45713418 | 1,99E-16 | -0,076 | 3310 |
| 17:77095668-77108864 | rs2004887 | 77090696 | 3,05E-11 | 0,051 | 2118 |
| 19:21561436-21697351 | rs8112960 | 21567211 | 7,45E-09 | -0,234 | 835 |
| 19:21562075-21568648 | rs8112960 | 21567211 | 4,41E-27 | -0,310 | 1852 |
| 20:19686792-19737926 | rs746804 | 19699330 | 1,67E-35 | -0,076 | 8389 |
| 20:23053705-23061258 | rs844813 | 23026996 | 9,05E-22 | -0,126 | 2231 |
| 20:36482649-36497390 | rs6070221 | 36513572 | 4,98E-16 | 0,054 | 8222 |
| 20:43509456-43510396 | rs13217 | 43486406 | 8,15E-13 | 0,054 | 3540 |
| 20:48317430-48329739 | rs17196752 | 48320675 | 9,65E-21 | -0,076 | 7605 |
| 20:48342664-48364866 | rs2904263 | 48339396 | 1,90E-31 | 0,133 | 4712 |
| 20:48360655-48371286 | rs11905919 | 48362378 | 3,71E-09 | -0,064 | 2497 |
| 21:24722966-24842127 | rs2829087 | 24744845 | 1,25E-16 | 0,070 | 952 |
| 21:29487672-29582397 | rs2257301 | 29287951 | 1,11E-09 | 0,125 | 5502 |
| 21:33352005-33359159 | rs928736 | 33361759 | 1,00E-27 | -0,075 | 8064 |
| 21:34225334-34271862 | rs35184820 | 34242811 | 4,14E-08 | -0,094 | 2216 |
| 21:34243100-34258130 | rs2834307 | 34252354 | 2,15E-07 | -0,085 | 2231 |
| 21:39032815-39062768 | rs2836664 | 39046495 | 1,85E-09 | -0,064 | 598 |
| 22:15462934-15514699 | rs9605146 | 15645194 | 5,12E-08 | 0,123 | 2290 |
| 22:19641380-19648967 | rs178255 | 19652836 | 2,34E-20 | -0,124 | 7347 |
| 22:27526671-27574547 | rs7287806 | 27543011 | 2,98E-17 | -0,050 | 5453 |
| 22:28431245-28445848 | rs1008515 | 28421145 | 5,30E-16 | 0,088 | 1332 |
| 22:28437809-28445703 | rs1008515 | 28421145 | 1,97E-14 | 0,101 | 1288 |
| 22:31102651-31110330 | rs742096 | 31139223 | 5,71E-33 | -0,169 | 3020 |
| 22:43908374-43938326 | rs547698 | 43906509 | 3,26E-54 | -0,100 | 10770 |
| 22:48328333-48336125 | rs2071904 | 48420481 | 3,14E-23 | -0,270 | 2536 |
