# Supplementary material for: Single Nucleotide Polymorphisms with Cis-Regulatory Effects on Long Non-Coding Transcripts in Human Primary Monocytes
Source: PLoS One. 2014 Jul 15;9(7):e102612. doi: 10.1371/journal.pone.0102612 (PMC4099216; doi:10.1371/journal.pone.0102612)
Supplement: Table S2 — SNPs associated with allele-specific expression of Refseq protein coding genes with published trait- or disease-associations from genome-wide association studies. (DOCX) [file pone.0102612.s006.docx]

**Table S2. SNPs associated with allele-specific expression of Refseq protein coding genes with published trait- or disease-associations from genome-wide association studies**

|  | **ASE** | | | | **GWAS** | |  |
| --- | --- | --- | --- | --- | --- | --- | --- |
| **GWAS genes** | **ASE  p-value** | **Slope^1^** | **LD SNP** | **LD** | **GWAS SNP^2^** | **Trait^3^** | |
| CLECL1 | 6.18E-95 | -0.32 |  |  | rs10466829 | Multiple sclerosis | |
| IL10 | 1.56E-71 | -0.26 |  |  | rs1518111 | Behcet's disease | |
| ABO | 2.69E-66 | -0.43 |  |  | rs643434 | Inflammatory biomarkers | |
| ABO | 2.69E-66 | -0.43 |  |  | rs657152 | Liver enzyme levels | |
| TNFSF15 | 6.53E-64 | 0.17 |  |  | rs4263839 | Crohn's disease | |
| TNFSF15 | 6.53E-64 | -0.17 |  |  | rs6478108 | Leprosy | |
| IL10 | 6.34E-62 | -0.25 |  |  | rs1800871 | Behcet's disease | |
| RTF1 | 2.71E-61 | 0.11 |  |  | rs2336725 | Height | |
| RNPEP | 6.11E-55 | 0.06 | rs4950752 | 1.0 | rs4950806 | Butyrylcholinesterase levels | |
| TP53INP1 | 3.92E-53 | 0.07 |  |  | rs896854 | Type 2 diabetes | |
| TTC39B | 9.24E-53 | -0.21 |  |  | rs471364 | HDL cholesterol | |
| ABO | 5.66E-52 | 0.44 |  |  | rs612169 | Metabolic traits | |
| SHBG | 5.72E-52 | 0.20 | rs1641535 | 1.0 | rs1641537 | Sex hormone-binding globulin levels | |
| ABO | 1.00E-50 | 0.44 |  |  | rs505922 | Protein quantitative trait loci | |
| ANGPTL3 | 1.04E-50 | 0.09 |  |  | rs10889353 | Triglycerides | |
| SIN3A, PTPN9 | 1.37E-50 | 0.20 |  |  | rs4886707 | Height | |
| ABO | 3.39E-50 | 0.43 |  |  | rs687621 | D-dimer levels | |
| DOCK7 | 8.78E-49 | 0.09 |  |  | rs1167998 | Triglycerides | |
| Intergenic | 1.89E-48 | -0.11 |  |  | rs2188962 | Crohn's disease | |
| NEGR1 | 1.94E-48 | 0.27 |  |  | rs2568958 | Body mass index | |
| PLEKHH1 | 7.92E-48 | -0.13 |  |  | rs1077989 | Phospholipid levels (plasma) | |
| TNFRSF10A, LOC389641 | 4.87E-47 | -0.07 |  |  | rs13278062 | Age-related macular degeneration | |
| LIPA | 2.76E-46 | 0.12 |  |  | rs1412444 | Coronary heart disease | |
| PLCL1 | 1.68E-44 | 0.14 |  |  | rs6738825 | Crohn's disease | |
| NR | 2.44E-44 | -0.30 |  |  | rs3764021 | Type 1 diabetes | |
| TBKBP1 | 4.18E-44 | 0.25 |  |  | rs8070463 | Ankylosing spondylitis | |
| SLC22A4, SLC22A5, IRF1, IL3 | 1.43E-43 | -0.10 |  |  | rs12521868 | Crohn's disease | |
| AHI1 | 8.94E-43 | -0.22 |  |  | rs11154801 | Multiple sclerosis | |
| ACADS | 3.52E-41 | -0.07 |  |  | rs2066938 | Metabolic traits | |
| PAX8 | 3.63E-41 | -0.36 | rs4849179 | 1.0 | rs11123170 | Renal function-related traits (BUN) | |
| DNAH11 | 6.68E-41 | -0.18 |  |  | rs4487645 | Multiple myeloma | |
| SHB | 9.86E-41 | -0.06 |  |  | rs7873102 | Brain structure | |
| ORMDL3,ZPBP2M,GSDML | 3.60E-40 | 0.07 |  |  | rs2305480 | Ulcerative colitis | |
| ATF7, ATP5G2 | 1.19E-38 | -0.11 |  |  | rs11170631 | Height | |
| PPM1E | 1.86E-38 | 0.18 |  |  | rs9303401 | Cognitive test performance | |
| VKORC1 | 2.40E-38 | 0.14 |  |  | rs10871454 | Warfarin maintenance dose | |
| FARP2 | 3.77E-38 | 0.05 |  |  | rs757978 | Chronic lymphocytic leukemia | |
| ADCY3, DNAJC27 | 3.99E-38 | -0.10 | rs6721750 | 1.0 | rs6545814 | Body mass index | |
| ORMDL3 | 6.98E-38 | 0.07 |  |  | rs2872507 | Crohn's disease | |
| KCTD7, RABGEF1 | 1.08E-37 | 0.22 | rs801191 | 0.889 | rs10263935 | Aortic root size | |
| HRK, FBXW8 | 6.88E-37 | -0.15 |  |  | rs7294919 | Brain structure | |
| VKORC1 | 1.38E-36 | 0.14 |  |  | rs9923231 | Warfarin maintenance dose | |
| ZBTB38 | 2.06E-36 | 0.06 |  |  | rs6763931 | Height | |
| ZBTB38 | 2.06E-36 | -0.06 |  |  | rs724016 | Height | |
| Intergenic | 2.34E-36 | 0.06 |  |  | rs7586970 | Coronary heart disease | |
| CRCP | 3.99E-36 | -0.20 |  |  | rs875971 | Aortic root size | |
| Intergenic | 4.05E-36 | 0.20 |  |  | rs6600671 | Hip geometry | |
| MERTK | 9.37E-36 | -0.12 |  |  | rs17174870 | Multiple sclerosis | |
| ZBTB38 | 1.23E-35 | 0.06 |  |  | rs6440003 | Height | |
| IKZF3 | 1.71E-35 | 0.07 |  |  | rs907092 | Primary biliary cirrhosis | |
| MAP2K5, LBXCOR1 | 2.03E-34 | -0.05 |  |  | rs2241423 | Body mass index | |
| PI16 | 3.18E-34 | -0.07 |  |  | rs1405069 | Chemerin levels | |
| TOB2 | 3.77E-34 | 0.06 | rs4820438 | 1.0 | rs4822024 | Vitiligo | |
| SIN3A | 6.63E-34 | 0.20 |  |  | rs8028182 | Sudden cardiac arrest | |
| KIF11 | 8.26E-34 | 0.11 |  |  | rs6583826 | Type 2 diabetes | |
| CHRNB4 | 3.55E-33 | -0.30 |  |  | rs950776 | Sudden cardiac arrest | |
| ATXN2,PTPN11,SH2B3 | 1.54E-32 | 0.05 |  |  | rs10774625 | Retinal vascular caliber | |
| SH2B3, ATXN2 | 1.54E-32 | -0.05 |  |  | rs653178 | Celiac disease | |
| SH2B3 | 2.69E-32 | 0.05 |  |  | rs3184504 | Eosinophil counts | |
| PLCE1,NOC3L | 2.13E-31 | 0.05 |  |  | rs3765524 | Esophageal cancer and gastric cancer | |
| MLF1IP | 2.51E-31 | 0.06 |  |  | rs2130392 | Kawasaki disease | |
| C11orf10 | 1.11E-30 | 0.18 |  |  | rs174538 | Phospholipid levels (plasma) | |
| TNFSF15, TNFSF8 | 1.86E-30 | 0.15 |  |  | rs3810936 | Crohn's disease | |
| GTF2E2 | 8.65E-30 | -0.05 |  |  | rs2978263 | Cognitive test performance | |
| ULK4 | 1.30E-29 | -0.05 |  |  | rs1052501 | Multiple myeloma | |
| ULK4 | 1.30E-29 | -0.05 |  |  | rs3774372 | Blood pressure | |
| NR | 1.80E-29 | -0.07 | rs4928431 | 1.0 | rs9500256 | Eosinophilic esophagitis (pediatric) | |
| NR | 2.01E-29 | -0.08 |  |  | rs1167796 | Systemic lupus erythematosus | |
| HHEX | 2.15E-29 | 0.11 |  |  | rs2497306 | Dehydroepiandrosterone sulphate levels | |
| Intergenic | 2.43E-29 | 0.08 |  |  | rs4699052 | Testicular germ cell tumor | |
| OXTR | 3.67E-29 | -0.26 |  |  | rs237899 | Depression and alcohol dependence | |
| MS4A | 4.25E-29 | 0.06 |  |  | rs1562990 | Alzheimer's disease | |
| TNFSF8, TNFSF15 | 1.57E-28 | 0.15 |  |  | rs4246905 | Ulcerative colitis | |
| CTF1 | 2.16E-28 | 0.13 |  |  | rs11649653 | Triglycerides | |
| MS4A4A | 2.24E-28 | 0.06 |  |  | rs4938933 | Alzheimer's disease (late onset) | |
| FADS1 | 2.98E-28 | 0.18 |  |  | rs174548 | Metabolite levels | |
| NR | 3.83E-28 | 0.08 | rs10476050 | 0.848 | rs11740562 | Bipolar disorder and schizophrenia | |
| MAPT | 4.11E-28 | 0.06 |  |  | rs8070723 | Parkinson's disease | |
| MICA | 4.23E-28 | -0.07 | rs2256175 | 1.0 | rs1063635 | Rheumatoid arthritis | |
| IL1RL1,IL18R1,IL18RAP, SLC9A4 | 7.32E-28 | 0.34 |  |  | rs13015714 | Celiac disease | |
| MICAL3 | 1.37E-27 | -0.14 |  |  | rs1076540 | Liver enzyme levels (gamma-glutamyl transferase) | |
| IL18RAP,IL12RL2,IL18R1,IL1RL1 | 2.22E-27 | 0.34 |  |  | rs2058660 | Crohn's disease | |
| SLC12A7 | 2.39E-27 | -0.09 |  |  | rs4580814 | Hematological and biochemical traits | |
| SLC22A5 | 2.73E-27 | 0.09 |  |  | rs2073643 | Asthma | |
| RBJ, ADCY3, POMC | 2.80E-27 | -0.09 | rs10182181 | 0.924 | rs713586 | Body mass index | |
| IL18RAP, IL18R1, IL1RL1, IL1RL2 | 5.31E-27 | -0.33 |  |  | rs917997 | Celiac disease | |
| MTCH2 | 6.35E-27 | -0.08 | rs1317149 | 1.0 | rs10838738 | Body mass index | |
| MAN2A2 | 9.76E-27 | 0.05 | rs871078 | 1.0 | rs2677744 | Attention deficit hyperactivity disorder | |
| LOC401365, LOC493754 | 9.86E-27 | -0.20 |  |  | rs801193 | Aortic root size | |
| PLEKHM1, MAPT, IMP5 | 2.15E-26 | 0.27 |  |  | rs11012 | Parkinson's disease | |
| CPT1B | 2.66E-26 | 0.17 |  |  | rs5770917 | Narcolepsy | |
| ORMDL3 | 3.09E-26 | -0.06 |  |  | rs7216389 | Asthma | |
| BARD1 | 3.27E-26 | 0.07 |  |  | rs6435862 | Neuroblastoma (high-risk) | |
| CHI3L1 | 3.49E-26 | -0.24 | rs946262 | 1.0 | rs4950928 | YKL-40 levels | |
| ZFP90 | 4.93E-26 | 0.06 |  |  | rs6499188 | Ulcerative colitis | |
| PAFAH1B2 | 5.19E-26 | -0.12 |  |  | rs7112513 | Protein quantitative trait loci | |
| ITIH3, ITIH4 | 7.34E-26 | -0.06 |  |  | rs2239547 | Schizophrenia | |
| ABO | 8.83E-26 | 0.43 |  |  | rs579459 | Soluble E-selectin levels | |
| HLA-A | 1.07E-25 | -0.07 | rs2517722 | 1.0 | rs2517713 | Nasopharyngeal carcinoma | |
| TNFSF14 | 1.10E-25 | 0.05 |  |  | rs1077667 | Multiple sclerosis | |
| HLA-C | 1.35E-25 | 0.12 |  |  | rs2395029 | Psoriasis | |
| LYZ, YEATS4, FRS2 | 4.69E-25 | -0.08 |  |  | rs317689 | Response to diuretic therapy | |
| PDE2A, ARAP1 | 5.12E-25 | -0.08 |  |  | rs3781913 | Rheumatoid arthritis | |
| Intergenic | 7.15E-25 | -0.05 |  |  | rs840616 | Coronary heart disease | |
| BGLAP,PAQR6,SMG5,TMEM79,C1orf85,VHLL,CCT3,C1orf182 | 7.70E-25 | -0.10 |  |  | rs6684514 | Hematological and biochemical traits | |
| ABO | 7.93E-25 | -0.43 |  |  | rs495828 | Angiotensin-converting enzyme activity | |
| TYW3, CRYZ | 1.44E-24 | 0.20 |  |  | rs3931020 | Resistin levels | |
| HHEX | 1.60E-24 | 0.10 |  |  | rs1111875 | Type 2 diabetes | |
| HHEX | 1.60E-24 | 0.10 |  |  | rs5015480 | Type 2 diabetes | |
| ZBTB38, RASA2 | 1.76E-24 | -0.05 | rs6767899 | 0.921 | rs9857275 | Prion diseases | |
| CNNM2 | 2.83E-24 | -0.17 |  |  | rs7914558 | Schizophrenia | |
| HLA-A | 3.64E-24 | -0.07 |  |  | rs2860580 | Nasopharyngeal carcinoma | |
| BAT1 | 4.85E-24 | -0.07 | rs3219190 | 0.889 | rs3853601 | Atopic dermatitis | |
| ORMDL3 | 9.38E-24 | -0.06 |  |  | rs2290400 | Type 1 diabetes | |
| BARD1 | 1.35E-23 | 0.07 |  |  | rs3768716 | Neuroblastoma | |
| CHD1L | 2.03E-23 | -0.08 |  |  | rs4950322 | Protein quantitative trait loci | |
| C11orf10 | 2.16E-23 | -0.17 |  |  | rs102275 | Metabolic syndrome | |
| FADS2 | 2.16E-23 | 0.17 |  |  | rs174574 | Phospholipid levels (plasma) | |
| GSDMB | 2.23E-23 | -0.06 |  |  | rs8067378 | Ulcerative colitis | |
| MS4A6A, MS4A4E | 2.60E-23 | -0.06 |  |  | rs610932 | Alzheimer's disease | |
| ALPL, NBPF3 | 3.31E-23 | 0.28 | rs1827293 | 0.855 | rs1976403 | Liver enzyme levels (alkaline phosphatase) | |
| Intergenic | 4.33E-23 | 0.06 |  |  | rs2766692 | Electroencephalographic traits in alcoholism | |
| BIN1 | 4.69E-23 | -0.19 |  |  | rs12989701 | Alzheimer's disease (late onset) | |
| IKZF3,ZPBP2,GSDMB,ORMDL3 | 5.21E-23 | 0.06 |  |  | rs9303277 | Primary biliary cirrhosis | |
| MHC | 6.16E-23 | 0.07 |  |  | rs4313034 | Graves' disease | |
| LMNB1 | 8.26E-23 | 0.09 |  |  | rs959573 | Parkinson's disease (motor and cognition) | |
| CRHR1 | 9.02E-23 | 0.28 |  |  | rs9303521 | Bone mineral density (hip) | |
| AL591893.2 | 1.49E-22 | 0.15 |  |  | rs3124314 | Hair morphology | |
| HLA-DRB9, HLA-DRB5, HLA-DRB1, HLA-DQA1, HLA-DQB1, HLA-DQA2 | 1.63E-22 | 0.12 | rs3135006 | 1.0 | rs3129720 | Hypothyroidism | |
| B3GALT4 | 1.85E-22 | -0.05 | rs1799908 | 1.0 | rs2254287 | LDL cholesterol | |
| GNL3, GLT8D1 | 2.24E-22 | -0.08 |  |  | rs11177 | Osteoarthritis | |
| NISCH, STAB1 | 3.92E-22 | 0.10 | rs4687617 | 1.0 | rs6784615 | Waist-hip ratio | |
| MTCH2, NDUFS3, CUGBP1 | 4.39E-22 | -0.07 | rs7124681 | 1.0 | rs3817334 | Body mass index | |
| FADS2, FADS1, FEN | 4.82E-22 | -0.16 |  |  | rs1535 | Response to statin therapy | |
| FADS1, FADS2 | 4.82E-22 | 0.16 |  |  | rs174546 | LDL cholesterol | |
| FADS1, FADS2, FADS3 | 4.82E-22 | -0.16 |  |  | rs174547 | HDL cholesterol | |
| TRIM66 | 4.83E-22 | -0.08 |  |  | rs4929923 | Menarche (age at onset) | |
| FEN1 | 7.69E-22 | 0.16 |  |  | rs4246215 | Phospholipid levels (plasma) | |
| CCND3 | 8.57E-22 | 0.05 |  |  | rs11968166 | Neutrophil count | |
| HLA-B | 1.34E-21 | -0.08 |  |  | rs13437082 | Height | |
| LOC400680, ZNF429 | 1.43E-21 | -0.09 |  |  | rs2562456 | Pain | |
| PVR | 1.77E-21 | 0.09 |  |  | rs7255066 | Multiple sclerosis | |
| DPYSL5 | 2.57E-21 | 0.14 |  |  | rs1371614 | Fasting glucose-related traits (interaction with BMI) | |
| SH2B3, ATXN2 | 2.58E-21 | -0.05 |  |  | rs11065987 | Hematocrit | |
| TNFRSF14, MMEL1 | 2.84E-21 | 0.08 |  |  | rs3748816 | Celiac disease | |
| FUT2 | 2.97E-21 | -0.06 |  |  | rs492602 | Vitamin B12 levels | |
| FUT2 | 2.97E-21 | 0.06 |  |  | rs516246 | Liver enzyme levels (gamma-glutamyl transferase) | |
| BOK, THAP4 | 3.41E-21 | 0.09 |  |  | rs12479254 | Brain structure | |
| ATM | 3.59E-21 | 0.05 |  |  | rs1801516 | Melanoma | |
| GP1BA | 4.40E-21 | 0.07 |  |  | rs6065 | Hematological and biochemical traits | |
| HHEX | 4.60E-21 | 0.10 |  |  | rs7923837 | Multiple sclerosis | |
| CDK4 | 5.17E-21 | -0.10 |  |  | rs10876993 | Celiac disease and Rheumatoid arthritis | |
| SNCA | 7.11E-21 | 0.07 |  |  | rs356220 | Parkinson's disease | |
| NUP205 | 8.86E-21 | 0.10 |  |  | rs4294134 | Paget's disease | |
| TPM1 | 1.05E-20 | -0.06 |  |  | rs11071720 | Mean platelet volume | |
| PALB2,NDUFAB1,DCTN5 | 1.63E-20 | -0.08 |  |  | rs420259 | Bipolar disorder | |
| TRAF3IP2 | 2.03E-20 | -0.05 | rs4509157 | 1.0 | rs458017 | Psoriasis | |
| HS6ST3 | 2.23E-20 | -0.13 |  |  | rs2038823 | Diabetic retinopathy | |
| PBRM1 | 3.34E-20 | 0.08 |  |  | rs2251219 | Major mood disorders | |
| APOA1,KIAA0999,LOC645044 | 3.49E-20 | 0.11 | rs2000614 | 1.0 | rs2075292 | Triglycerides | |
| ICOSLG | 4.83E-20 | -0.08 |  |  | rs762421 | Crohn's disease | |
| MAPT | 7.02E-20 | 0.05 |  |  | rs12185268 | Parkinson's disease | |
| ICOSLG | 8.25E-20 | -0.08 | rs7282490 | 0.962 | rs2838519 | Ulcerative colitis | |
| FUT2 | 1.49E-19 | -0.06 |  |  | rs503279 | Metabolic traits | |
| FUT2 | 1.49E-19 | 0.06 |  |  | rs504963 | Crohn's disease | |
| FUT2 | 1.49E-19 | 0.06 |  |  | rs602662 | Folate pathway vitamin levels | |
| NAPRT1 | 1.84E-19 | -0.12 |  |  | rs2290416 | Attention deficit hyperactivity disorder | |
| LOC343052 | 2.19E-19 | 0.05 |  |  | rs4434872 | Conduct disorder (symptom count) | |
| TNFRSF14, MMEL1, PLCH2, C1orf93 | 2.45E-19 | -0.08 |  |  | rs734999 | Ulcerative colitis | |
| KIAA1598, VAX1 | 2.91E-19 | 0.10 |  |  | rs7078160 | Orofacial clefts | |
| CFB | 3.69E-19 | 0.06 |  |  | rs541862 | Age-related macular degeneration | |
| C11orf9 | 3.96E-19 | -0.16 |  |  | rs174535 | Phospholipid levels (plasma) | |
| TNFSF13 | 4.97E-19 | 0.19 |  |  | rs3803800 | IgA nephropathy | |
| GP6 | 5.21E-19 | -0.26 |  |  | rs1671152 | Platelet aggregation | |
| FUT2, RASIP1 | 6.21E-19 | -0.06 | rs676388 | 1.0 | rs281379 | Crohn's disease | |
| ICOSLG | 8.84E-19 | -0.08 |  |  | rs4819388 | Celiac disease | |
| SLC22A4 | 9.67E-19 | 0.08 |  |  | rs272889 | Metabolic traits | |
| SLC22A5 | 9.67E-19 | 0.08 |  |  | rs274546 | Height | |
| KLHDC8B | 1.03E-18 | 0.14 |  |  | rs7617480 | Menarche (age at onset) | |
| ACTA2, FAS | 1.22E-18 | -0.09 |  |  | rs2234978 | Immunoglobulin A | |
| HLA-DPA1, HLA-DPB1, HLA-DPB2 | 1.38E-18 | -0.05 |  |  | rs1883414 | Nephropathy | |
| HLA | 1.43E-18 | -0.08 |  |  | rs2247056 | Triglycerides | |
| FCRL3 | 1.63E-18 | 0.09 |  |  | rs3761959 | Graves' disease | |
| FCRL3 | 1.63E-18 | -0.09 |  |  | rs7528684 | Type 1 diabetes autoantibodies | |
| MMEL1,TNFRSF14 | 1.88E-18 | 0.08 |  |  | rs3890745 | Rheumatoid arthritis | |
| BCO2 | 2.10E-18 | 0.07 | rs5744256 | 1.0 | rs1834481 | Interleukin-18 levels | |
| CHRNA3, CHRNA5, PSMA4, LOC123688 | 3.39E-18 | 0.28 |  |  | rs8034191 | Lung cancer | |
| CLN8 | 3.88E-18 | -0.05 |  |  | rs11986414 | Gaucher disease severity | |
| Intergenic | 4.49E-18 | -0.20 |  |  | rs11249433 | Breast cancer | |
| HLA-DPB1 | 4.85E-18 | 0.06 |  |  | rs9277535 | Hepatitis B | |
| EREG | 1.43E-17 | -0.12 |  |  | rs1350666 | Attention deficit hyperactivity disorder | |
| ALPL | 1.99E-17 | -0.25 | rs10799702 | 0.821 | rs10799701 | Metabolic traits | |
| BET1L | 2.15E-17 | 0.05 |  |  | rs11602954 | Mean platelet volume | |
| GPLD1 | 2.48E-17 | -0.11 |  |  | rs9467160 | Liver enzyme levels | |
| HNRNPC | 3.08E-17 | 0.07 |  |  | rs17197037 | Bipolar disorder | |
| HLA-DQB2, HLA-DOB | 3.09E-17 | 0.17 |  |  | rs2857151 | Kawasaki disease | |
| USP49,MED20,BYSL,CCND3 | 4.09E-17 | -0.07 |  |  | rs3218097 | Hematological and biochemical traits | |
| LCAT | 4.93E-17 | -0.07 |  |  | rs255049 | HDL cholesterol | |
| CHRNA5 | 5.51E-17 | -0.27 |  |  | rs2036527 | Pulmonary function | |
| CARD9, INPP5E, SDCCAG3, SEC16A, SNAPC4 | 5.77E-17 | 0.05 |  |  | rs10781499 | Ulcerative colitis | |
| CARD9 | 5.77E-17 | 0.05 |  |  | rs10781500 | Ulcerative colitis | |
| CARD9 | 5.77E-17 | 0.05 |  |  | rs4077515 | Ulcerative colitis | |
| GFOD2,LCAT | 6.13E-17 | -0.07 |  |  | rs12449157 | HDL cholesterol | |
| CHRNA3,CHRNA5,CHRNB4 | 6.56E-17 | -0.26 |  |  | rs1051730 | Nicotine dependence | |
| RASIP1,IZUMO1,FUT1,FUT2,CA11,FGF21,FLJ36070 | 7.53E-17 | -0.06 | rs973579 | 0.851 | rs2287921 | Retinal vascular caliber | |
| CD69 | 7.89E-17 | 0.25 |  |  | rs4763879 | Type 1 diabetes | |
| C11orf73 | 9.54E-17 | -0.08 |  |  | rs6592284 | Cognitive performance | |
| GAB2 | 1.03E-16 | -0.07 | rs7112234 | 1.0 | rs2373115 | Alzheimer's disease (late onset) | |
| HLA-DP, COL11A2 | 1.57E-16 | -0.05 | rs3117213 | 1.0 | rs3117242 | Antineutrophil cytoplasmic antibody-associated vasculitis | |
| MICA | 1.68E-16 | -0.07 |  |  | rs2596542 | Hepatocellular carcinoma | |
| GDF5, UQCC | 2.30E-16 | -0.05 | rs224415 | 0.874 | rs2236164 | Height | |
| NBPF3, ALPL, RAP1GAP | 2.33E-16 | 0.25 |  |  | rs1780324 | Liver enzyme levels | |
| HBS1L,MYB | 3.02E-16 | -0.14 |  |  | rs7775698 | Hematology traits | |
| FCER1A | 3.74E-16 | -0.28 |  |  | rs2251746 | IgE levels | |
| CFB,C2 | 5.35E-16 | -0.06 |  |  | rs641153 | Age-related macular degeneration | |
| CATSPER4 | 5.77E-16 | 0.14 |  |  | rs11809207 | Height | |
| CRHR1, MAPT | 5.97E-16 | -0.28 |  |  | rs11655470 | Head circumference (infant) | |
| C8orf13, BLK | 6.33E-16 | -0.12 |  |  | rs13277113 | Systemic lupus erythematosus | |
| BLK | 6.33E-16 | -0.12 |  |  | rs2736340 | Rheumatoid arthritis | |
| NR | 7.62E-16 | -0.09 | rs4938493 | 1.0 | rs17122021 | Pain | |
| CDH1 | 1.10E-15 | -0.05 |  |  | rs9929218 | Colorectal cancer | |
| ALPL | 1.24E-15 | -0.25 |  |  | rs4654748 | Folate pathway vitamin levels | |
| KIAA1271 | 1.78E-15 | 0.06 |  |  | rs4815617 | Asthma | |
| TIMD4,HAVCR1 | 2.17E-15 | -0.15 |  |  | rs6882076 | Cholesterol, total | |
| LOC100289506 | 2.40E-15 | -0.10 |  |  | rs758944 | Multiple sclerosis | |
| LOC285830 | 2.49E-15 | 0.06 |  |  | rs2523395 | Prostate cancer (gene x gene interaction) | |
| DUT | 3.22E-15 | -0.36 |  |  | rs11637235 | Protein quantitative trait loci | |
| SLC22A12 | 3.33E-15 | 0.33 | rs506338 | 1.0 | rs504915 | Renal function-related traits (urea) | |
| FRAP1 | 3.53E-15 | 0.09 |  |  | rs17036350 | Corneal curvature | |
| MFAP2 | 4.57E-15 | -0.08 | rs9435734 | 1.0 | rs2284746 | Height | |
| NFYC | 8.43E-15 | 0.06 |  |  | rs4660456 | Platelet counts | |
| HLA-DQA2 | 9.50E-15 | -0.24 |  |  | rs2301271 | Systemic lupus erythematosus | |
| HAL-DQB2 | 9.50E-15 | -0.24 |  |  | rs7453920 | Hepatitis B | |
| OPTN | 1.75E-14 | -0.15 |  |  | rs1561570 | Paget's disease | |
| Intergenic | 2.62E-14 | -0.06 |  |  | rs2302189 | Dental caries | |
| PBX2 | 2.75E-14 | -0.05 |  |  | rs204993 | Asthma | |
| ERGIC3 | 3.43E-14 | -0.06 |  |  | rs2277862 | Cholesterol, total | |
| C2orf74,REL | 4.17E-14 | 0.10 |  |  | rs10181042 | Crohn's disease | |
| SNCA | 4.60E-14 | -0.06 |  |  | rs2736990 | Parkinson's disease | |
| HBS1L, MYB | 4.61E-14 | -0.13 |  |  | rs9376092 | Beta thalassemia/hemoglobin E disease | |
| MHC | 5.45E-14 | -0.07 |  |  | rs7743761 | Ankylosing spondylitis | |
| IL3, ACSL6, P4HA2, PDLIM4, SLC22A4 | 6.46E-14 | -0.06 | rs3864277 | 0.961 | rs3091338 | Crohn's disease | |
| TRIM47 | 1.10E-13 | -0.10 |  |  | rs1055129 | White matter hyperintensity burden | |
| PKNOX1 | 1.10E-13 | -0.06 |  |  | rs234720 | Cognitive test performance | |
| ACE | 1.15E-13 | 0.06 |  |  | rs4329 | Metabolic traits | |
| IRF1 | 1.50E-13 | 0.07 |  |  | rs2070729 | Platelet counts | |
| ANGPTL3, DOCK7, ATG4C | 1.75E-13 | -0.07 |  |  | rs12130333 | Triglycerides | |
| MLN | 2.03E-13 | 0.08 |  |  | rs2274459 | Obesity (extreme) | |
| RFPL1, AP1B1, THOC5, NF2 | 2.09E-13 | -0.10 |  |  | rs13053817 | Carotid atherosclerosis in HIV infection | |
| SLCO6A1 | 2.28E-13 | 0.05 |  |  | rs1502844 | Schizophrenia | |
| FUT2 | 2.29E-13 | -0.05 |  |  | rs281377 | Liver enzyme levels (alkaline phosphatase) | |
| MAPT | 2.36E-13 | 0.05 |  |  | rs242557 | Progressive supranuclear palsy | |
| GSDMA | 2.70E-13 | -0.05 |  |  | rs3894194 | Asthma | |
| CHRNA5 | 2.73E-13 | 0.32 |  |  | rs667282 | Smoking behavior | |
| FBXO40 | 3.33E-13 | -0.05 |  |  | rs3772130 | Cognitive performance | |
| MHC, C6orf10 | 3.39E-13 | -0.06 |  |  | rs3130340 | Bone mineral density (spine) | |
| BRSK1, TMEM224, SUV420H2 | 3.63E-13 | -0.06 |  |  | rs1172822 | Menopause (age at onset) | |
| TF | 3.72E-13 | 0.13 |  |  | rs3811647 | Iron status biomarkers | |
| HBS1L, MYB | 4.11E-13 | 0.13 |  |  | rs4895441 | Mean corpuscular volume | |
| HLA-DRB1 | 4.48E-13 | -0.12 |  |  | rs9271366 | Multiple sclerosis | |
| EDC4 | 4.59E-13 | -0.06 |  |  | rs8060686 | Coronary heart disease | |
| SLC28A1, ZNF592, ALPK3 | 4.86E-13 | -0.05 | rs12442557 | 1.0 | rs3743162 | Alzheimer's disease (age of onset) | |
| NR | 5.11E-13 | 0.33 |  |  | rs8042374 | Lung cancer | |
| TACC3,TMEM129,SLBP,FGFR3 | 5.54E-13 | 0.05 |  |  | rs798766 | Urinary bladder cancer | |
| ACSM1 | 6.23E-13 | -0.05 |  |  | rs433598 | Schizophrenia | |
| ACE | 6.39E-13 | -0.06 |  |  | rs4343 | Angiotensin-converting enzyme activity | |
| LBH | 7.21E-13 | 0.10 |  |  | rs7579944 | Celiac disease and Rheumatoid arthritis | |
| NTHL1 | 7.75E-13 | 0.06 |  |  | rs2516739 | Longevity | |
| HLA-DRB1 | 8.20E-13 | -0.12 |  |  | rs3129889 | Multiple sclerosis | |
| HLA-DRA | 8.20E-13 | 0.12 |  |  | rs3135388 | Multiple sclerosis | |
| BLK | 8.84E-13 | -0.12 |  |  | rs1600249 | Rheumatoid arthritis | |
| DDR1, VARS2, DPCR1 | 8.84E-13 | -0.15 |  |  | rs7756521 | HIV-1 control | |
| NOTCH4, C6orf10 | 1.08E-12 | 0.08 |  |  | rs3130320 | Systemic lupus erythematosus | |
| HLA-A | 1.12E-12 | 0.06 |  |  | rs2523946 | IgA nephropathy | |
| PSCA | 1.22E-12 | -0.11 |  |  | rs2294008 | Bladder cancer | |
| WDR66 | 1.27E-12 | -0.12 |  |  | rs7961894 | Mean platelet volume | |
| NR | 1.33E-12 | 0.06 |  |  | rs4380451 | Bipolar disorder | |
| Intergenic | 1.40E-12 | 0.05 |  |  | rs499818 | Major CVD | |
| C6orf10 | 1.46E-12 | -0.05 |  |  | rs3129943 | Asthma | |
| SLC22A12 | 1.53E-12 | -0.31 | rs9734313 | 1.0 | rs505802 | Uric acid levels | |
| LPL | 1.60E-12 | 0.19 |  |  | rs17091905 | Cardiovascular disease risk factors | |
| BCO2 | 1.66E-12 | -0.05 | rs2043055 | 0.885 | rs2115763 | Interleukin-18 levels | |
| HLA-DRB1 | 3.01E-12 | 0.08 |  |  | rs3129934 | Multiple sclerosis | |
| C6orf106 | 3.73E-12 | 0.09 |  |  | rs2814944 | HDL cholesterol | |
| CDKN2A,CDKN2B | 3.89E-12 | -0.07 |  |  | rs1011970 | Breast cancer | |
| LPL | 4.05E-12 | 0.18 |  |  | rs10096633 | Metabolic traits | |
| FADS3 | 5.13E-12 | -0.13 |  |  | rs174448 | Phospholipid levels (plasma) | |
| BACH2 | 5.16E-12 | -0.07 |  |  | rs3757247 | Type 1 diabetes | |
| C20orf132 | 5.35E-12 | 0.05 |  |  | rs8115854 | Hippocampal atrophy | |
| DCBLD1 | 5.82E-12 | 0.10 |  |  | rs210648 | Economic and political preferences | |
| RNF19A, ANKRD46 | 5.91E-12 | -0.11 |  |  | rs1371867 | Atrioventricular conduction | |
| LPL | 6.04E-12 | -0.19 |  |  | rs12678919 | HDL cholesterol | |
| PCSK9 | 7.11E-12 | 0.29 |  |  | rs11206510 | LDL cholesterol | |
| C6orf106 | 7.12E-12 | 0.09 |  |  | rs2814993 | Height | |
| ARNT, SETDB1, LASS2, ANXA9, MCL1, CTSK | 7.34E-12 | -0.06 |  |  | rs7412746 | Melanoma | |
| BTNL2 | 7.99E-12 | -0.08 |  |  | rs3117098 | Asthma | |
| SLC15A2 | 8.64E-12 | 0.05 |  |  | rs4285028 | Multiple sclerosis | |
| BAIAP2L1 | 9.04E-12 | -0.06 |  |  | rs9649213 | Prostate cancer (gene x gene interaction) | |
| NKAIN3 | 1.09E-11 | -0.07 |  |  | rs7834588 | Response to Vitamin E supplementation | |
| RP11-24C3.2 | 1.10E-11 | 0.06 | rs6442117 | 0.887 | rs9876781 | Longevity | |
| LPL | 1.10E-11 | 0.18 |  |  | rs7841189 | Metabolic syndrome | |
| PCSK7 | 1.29E-11 | -0.13 |  |  | rs508487 | Cardiovascular disease risk factors | |
| HLA-C | 1.41E-11 | 0.08 | rs2524069 | 1.0 | rs3134792 | Psoriasis | |
| FAM108C1 | 1.49E-11 | 0.08 |  |  | rs12148329 | Immune response to smallpox vaccine (IL-6) | |
| CDKN2A | 1.66E-11 | -0.07 | rs2811708 | 0.899 | rs3731211 | Platelet counts | |
| RPN2 | 1.80E-11 | 0.05 |  |  | rs6031882 | Hippocampal atrophy | |
| MHC | 1.99E-11 | 0.07 | rs1063355 | 1.0 | rs9272346 | Type 1 diabetes | |
| IL18R1 | 2.48E-11 | 0.19 |  |  | rs3771166 | Asthma | |
| FUT8 | 2.95E-11 | 0.06 |  |  | rs10483776 | N-glycan levels | |
| BIK | 2.99E-11 | 0.05 |  |  | rs742134 | Prostate cancer | |
| CYP4F8 | 3.14E-11 | -0.16 |  |  | rs3764563 | Inflammatory biomarkers | |
| BACH2 | 3.24E-11 | -0.07 |  |  | rs1847472 | Crohn's disease | |
| TSEN2 | 3.39E-11 | -0.06 |  |  | rs6766510 | Prostate cancer (gene x gene interaction) | |
| Intergenic | 5.53E-11 | -0.06 |  |  | rs9917256 | Parkinson's disease | |
| PRKAR2B | 5.68E-11 | -0.11 |  |  | rs7798500 | Adverse response to lamotrigine and phenytoin | |
| C6orf10 | 8.64E-11 | -0.05 |  |  | rs2050190 | Activated partial thromboplastin time | |
| FAM167A, BLK | 9.12E-11 | 0.10 |  |  | rs2254546 | Kawasaki disease | |
| BLK | 9.12E-11 | 0.10 |  |  | rs7812879 | Systemic lupus erythematosus | |
| EPS15L1 | 9.62E-11 | -0.06 | rs7256818 | 1.0 | rs10411936 | Multiple sclerosis | |
| WDR12 | 9.73E-11 | 0.10 |  |  | rs6725887 | Myocardial infarction (early onset) | |
| GNL3 | 1.07E-10 | 0.06 |  |  | rs1108842 | Adiponectin levels | |
| MHC, other genes | 1.07E-10 | -0.05 |  |  | rs886424 | Bipolar disorder and schizophrenia | |
| TAS2R38 | 1.09E-10 | 0.21 | rs4726481 | 0.959 | rs713598 | Bitter taste response | |
| CYP2C18 | 1.65E-10 | -0.05 |  |  | rs12772169 | Acenocoumarol maintenance dosage | |
| SHBG | 1.67E-10 | 0.05 |  |  | rs12150660 | Testosterone levels | |
| MPDU1 | 1.67E-10 | -0.05 |  |  | rs4227 | IgA nephropathy | |
| INTS12, NPNT, FLJ20184, GSTCD | 1.68E-10 | 0.06 | rs10050159 | 1.0 | rs11727189 | Pulmonary function | |
| CD40 | 1.93E-10 | 0.09 |  |  | rs1569723 | Kawasaki disease | |
| CD40 | 1.93E-10 | -0.09 |  |  | rs4810485 | Rheumatoid arthritis | |
| CD40 | 1.93E-10 | 0.09 |  |  | rs6074022 | Multiple sclerosis | |
| ATP13A2, SDHB | 1.98E-10 | 0.07 | rs2871776 | 0.883 | rs3738814 | Height | |
| UBXD2 | 2.06E-10 | 0.09 |  |  | rs6430585 | Corneal structure | |
| GNL3 | 2.71E-10 | -0.06 |  |  | rs2590838 | Adiponectin levels | |
| GSTCD | 2.87E-10 | -0.06 |  |  | rs10516526 | Pulmonary function | |
| MHC | 3.26E-10 | 0.16 |  |  | rs2269426 | Eosinophil counts | |
| GSDMA | 4.32E-10 | -0.05 |  |  | rs3859192 | White blood cell count | |
| CHRNA3 | 4.95E-10 | -0.20 |  |  | rs12914385 | Pulmonary function | |
| ALDH5A1, GPLD1 | 5.59E-10 | 0.08 |  |  | rs1883415 | Liver enzyme levels (alkaline phosphatase) | |
| FGB, FGA, FGG | 5.68E-10 | -0.05 |  |  | rs6056 | Fibrinogen | |
| ETS1 | 5.78E-10 | -0.06 |  |  | rs11221332 | Celiac disease | |
| HLA-DQA2 | 6.12E-10 | 0.14 |  |  | rs2858331 | IgE levels | |
| HSP90B1 | 7.30E-10 | -0.10 |  |  | rs1165669 | Coronary heart disease | |
| C5orf30 | 7.52E-10 | 0.10 |  |  | rs26232 | Rheumatoid arthritis | |
| HBS1L, MYB | 8.12E-10 | 0.12 |  |  | rs9494145 | Red blood cell traits | |
| SLC4A7 | 9.67E-10 | -0.09 |  |  | rs4973768 | Breast cancer | |
| HLA-C | 9.96E-10 | -0.06 | rs3873379 | 0.851 | rs9461688 | Protein quantitative trait loci | |
| IL1RL1 | 1.17E-09 | -0.11 | rs12905 | 0.91 | rs1420101 | Eosinophil counts | |
| NR | 1.26E-09 | -0.08 |  |  | rs4799088 | Amyotrophic lateral sclerosis | |
| STARD13 | 1.27E-09 | -0.06 | rs602133 | 0.813 | rs9315204 | Intracranial aneurysm | |
| LOC654254 | 1.31E-09 | 0.14 |  |  | rs11723261 | Immune response to smallpox vaccine (IL-6) | |
| BACH2, MAP3K7 | 1.34E-09 | -0.06 |  |  | rs10806425 | Celiac disease | |
| POL3S | 1.60E-09 | -0.07 |  |  | rs11865038 | Parkinson's disease | |
| HCG27, HLA-C | 1.70E-09 | -0.05 | rs2894181 | 0.852 | rs3869109 | Coronary heart disease | |
| KIAA1598 | 1.71E-09 | -0.06 |  |  | rs740363 | Heart failure | |
| HERV, FRD | 1.78E-09 | 0.35 |  |  | rs4711171 | Phospholipid levels (plasma) | |
| RORA | 1.96E-09 | 0.12 |  |  | rs12591650 | Subcutaneous adipose tissue | |
| AL450992.4 | 2.05E-09 | 0.17 |  |  | rs10788819 | Hair morphology | |
| BACH2, MAP3K7 | 2.16E-09 | -0.07 |  |  | rs370409 | Graves' disease | |
| ACAN | 2.87E-09 | 0.15 |  |  | rs2351491 | Height | |
| Intergenic | 3.02E-09 | 0.07 |  |  | rs11026412 | Parkinson's disease | |
| PARK16, NUCKS1 | 3.38E-09 | 0.15 |  |  | rs823128 | Parkinson's disease | |
| C5orf56 | 3.83E-09 | 0.05 |  |  | rs11745587 | Asthma | |
| NR | 4.51E-09 | 0.21 |  |  | rs9469220 | Crohn's disease | |
| ZNF292 | 4.53E-09 | -0.05 | rs370228 | 0.906 | rs1925690 | MRI atrophy measures | |
| CCHCR1 | 4.55E-09 | 0.05 |  |  | rs130067 | Prostate cancer | |
| KIF16B | 4.72E-09 | -0.10 |  |  | rs6044003 | Intelligence | |
| ALPL,NBPF3 | 5.30E-09 | -0.19 |  |  | rs1697421 | Phosphorus levels | |
| SDAD1 | 5.91E-09 | -0.07 | rs6826085 | 1.0 | rs2273 | Longevity | |
| SLC14A1 | 5.93E-09 | -0.14 |  |  | rs7238033 | Bladder cancer | |
| TMEM110 | 6.94E-09 | -0.10 |  |  | rs3796352 | Immune reponse to smallpox (secreted IL-2) | |
| HLA | 6.97E-09 | 0.05 |  |  | rs9468925 | Vitiligo | |
| HLA class III | 8.17E-09 | -0.15 |  |  | rs185819 | Height | |
| OSBPL7 | 8.93E-09 | 0.10 | rs7214410 | 0.816 | rs7206971 | Cholesterol, total | |
| HCP5 | 9.34E-09 | 0.06 |  |  | rs9469003 | Stevens-Johnson syndrome and toxic epidermal necrolysis (SJS-TEN) | |
| SYCP2L | 9.83E-09 | 0.34 | rs8523 | 0.921 | rs4713103 | Phospholipid levels (plasma) | |
| SYCP2L | 9.83E-09 | -0.34 | rs2295600 | 1.0 | rs6918936 | Phospholipid levels (plasma) | |
| ZNRD1, RNF39 | 1.04E-08 | -0.06 | rs3132685 | 1.0 | rs8321 | AIDS progression | |
| LCAT | 1.05E-08 | 0.06 |  |  | rs16942887 | HDL cholesterol | |
| AXIN1 | 1.06E-08 | 0.06 |  |  | rs9921222 | Bone mineral density | |
| HLA-DQB1 | 1.11E-08 | 0.23 |  |  | rs2856718 | Hepatitis B | |
| HLA-DRA | 1.14E-08 | -0.08 |  |  | rs6903608 | Hodgkin's lymphoma | |
| MYH15 | 1.17E-08 | -0.09 |  |  | rs2603127 | Hemostatic factors and hematological phenotypes | |
| NR | 1.21E-08 | -0.06 |  |  | rs16966142 | Caffeine consumption | |
| MC1R | 1.23E-08 | -0.06 |  |  | rs258322 | Black vs. red hair color | |
| IRF6 | 1.24E-08 | 0.13 | rs861020 | 1.0 | rs642961 | Orofacial clefts | |
| CRLF3, ATAD5, CENTA2, RNF135 | 1.28E-08 | 0.06 |  |  | rs3760318 | Height | |
| MHC, other genes | 1.29E-08 | -0.05 |  |  | rs2524005 | Bipolar disorder and schizophrenia | |
| GNAT2, GNAI3, AMPD2 | 1.34E-08 | -0.19 | rs2304355 | 0.913 | rs6537837 | Major depressive disorder | |
| ELOVL2 | 1.37E-08 | -0.34 |  |  | rs3734398 | Phospholipid levels (plasma) | |
| PMS1 | 1.87E-08 | 0.07 |  |  | rs5743030 | Subcutaneous adipose tissue | |
| LCAT | 1.90E-08 | 0.06 |  |  | rs2271293 | HDL cholesterol | |
| TRIM2 | 2.40E-08 | -0.06 |  |  | rs12644284 | Multiple sclerosis | |
| Unknown | 2.56E-08 | 0.12 |  |  | rs3764147 | Crohn's disease | |
| IHH, CRYBA2, FEV, SLC23A3, TUBA1 | 2.65E-08 | 0.06 |  |  | rs1052483 | Height | |
| NR | 2.83E-08 | 0.07 | rs3763288 | 0.879 | rs9378249 | Bipolar disorder | |
| UBE2D1 | 2.91E-08 | -0.06 |  |  | rs16912145 | Brain imaging | |
| LPL | 3.17E-08 | -0.11 |  |  | rs301 | Metabolic syndrome (bivariate traits) | |
| Intergenic | 3.56E-08 | -0.16 | rs12660883 | 1.0 | rs12526186 | Response to antipsychotic treatment | |
| MRAS | 3.79E-08 | 0.06 |  |  | rs2306374 | Coronary heart disease | |
| PRKCZ | 3.79E-08 | 0.16 |  |  | rs3753242 | Reasoning | |
| MRAS | 3.79E-08 | -0.06 |  |  | rs9818870 | Coronary heart disease | |
| FXR2, SHBG, SAT2, ATP1B2 | 3.86E-08 | 0.09 |  |  | rs727428 | Sex hormone-binding globulin levels | |
| PTGFRN | 4.07E-08 | -0.09 | rs7365057 | 1.0 | rs2806864 | Erectile dysfunction and prostate cancer treatment | |
| HLA-A | 4.19E-08 | 0.05 | rs2735097 | 1.0 | rs2571391 | IgE levels | |
| ATM, C111orf65 | 4.52E-08 | -0.05 |  |  | rs11212617 | Response to metformin | |
| PLCL1 | 4.81E-08 | -0.11 |  |  | rs7595412 | Hip bone size | |
| STBD1 | 5.37E-08 | 0.12 |  |  | rs6812193 | Parkinson's disease | |
| LPL | 5.40E-08 | -0.10 |  |  | rs13702 | HDL Cholesterol - Triglycerides (HDLC-TG) | |
| LPL | 5.40E-08 | 0.10 |  |  | rs15285 | Triglycerides-Blood Pressure (TG-BP) | |
| LMAN1L, EDC3, CYP1A2, CYP1A1, CSK | 5.41E-08 | 0.05 |  |  | rs2470893 | Caffeine consumption | |
| 23231 | 5.46E-08 | -0.10 |  |  | rs959903 | Non-alcoholic fatty liver disease histology (other) | |
| LAMC2 | 5.56E-08 | 0.16 |  |  | rs525410 | Systemic lupus erythematosus | |
| BTNL2 | 5.57E-08 | 0.06 |  |  | rs2076529 | Waist-hip ratio | |
| PLA2G7 | 5.58E-08 | 0.07 |  |  | rs1805017 | Lipoprotein-associated phospholipase A2 activity and mass | |
| HLA-DQB1 | 5.62E-08 | 0.11 |  |  | rs7775228 | Asthma | |
| HLA-DQA1, HLA-DQA2 | 7.27E-08 | 0.06 |  |  | rs2647012 | Systemic lupus erythematosus | |
| CPEB1 | 7.62E-08 | -0.23 | rs8043401 | 0.918 | rs783540 | Chronic lymphocytic leukemia | |
| Intergenic | 7.78E-08 | 0.05 |  |  | rs9262632 | HIV-1 control | |
| HLA-DQA2 | 8.10E-08 | 0.06 |  |  | rs9275572 | Alopecia areata | |
| SLC45A3, NUKS1 | 9.04E-08 | 0.08 | rs3805 | 1.0 | rs12748961 | White blood cell types | |
| FADS3 | 1.14E-07 | 0.13 |  |  | rs1000778 | Sphingolipid levels | |
| c6orf10, BTNL2, HLA-DQB1 | 1.14E-07 | -0.24 |  |  | rs9357152 | Primary biliary cirrhosis | |
| CCR6 | 1.17E-07 | 0.10 |  |  | rs3093023 | Rheumatoid arthritis | |
| CCR6 | 1.17E-07 | 0.10 |  |  | rs3093024 | Rheumatoid arthritis | |
| NR | 1.24E-07 | 0.06 |  |  | rs2326679 | Menopause (age at onset) | |
| HLA-DQA2 | 1.27E-07 | 0.20 |  |  | rs3916765 | Type 2 diabetes | |
| ADAMTS10, MYO1F, PRAM1, OR2Z1 | 1.31E-07 | -0.05 |  |  | rs7249094 | Height | |
| RBP4 | 1.39E-07 | -0.07 |  |  | rs10882272 | Retinol levels | |
| SLA | 1.41E-07 | 0.10 |  |  | rs853308 | Vitiligo | |
| BACH2 | 1.42E-07 | 0.06 |  |  | rs12212193 | Multiple sclerosis | |
| MYCBPAP | 1.46E-07 | 0.23 | rs11869714 | 1.0 | rs6504663 | Visceral fat | |
| SLC22A18 | 1.49E-07 | 0.08 |  |  | rs16928809 | Bilirubin levels | |
| BTN1A1 | 1.56E-07 | 0.10 |  |  | rs13194984 | Iron status biomarkers | |
| NR | 1.56E-07 | -0.10 |  |  | rs712039 | Tuberculosis | |
| NOG, DGKE, TRIM25, COIL, RISK | 1.65E-07 | -0.06 |  |  | rs4794665 | Height | |
| DLG5 | 1.81E-07 | 0.06 | rs10430531 | 0.915 | rs754466 | Liver enzyme levels (gamma-glutamyl transferase) | |
| ENSG00000173957, UBXD4, FKBP1B, FLJ21945 | 1.81E-07 | 0.07 |  |  | rs7561273 | Quantitative traits | |
| PADI4, PADI6,RCC2, ARHGEF10L | 1.86E-07 | -0.19 |  |  | rs7538876 | Basal cell carcinoma | |
| HLA-A | 1.97E-07 | -0.05 |  |  | rs2523822 | Drug-induced liver injury (amoxicillin-clavulanate) | |
| BDNF | 2.13E-07 | -0.07 | rs10734394 | 1.0 | rs988712 | Obesity | |
| HLA-DRB1, HLA-DQA1, HLA-DQB1 | 2.13E-07 | -0.06 |  |  | rs9275596 | Nephropathy | |
| GCM2, SYCP2L | 2.51E-07 | 0.40 |  |  | rs2153157 | Menarche and menopause (age at onset) | |
| HLA-DRA | 3.03E-07 | -0.14 | rs9268832 | 0.961 | rs7192 | Non-obstructive azoospermia | |
| BOLL, PLCL1 | 3.18E-07 | -0.07 |  |  | rs700651 | Intracranial aneurysm | |
| CBX1 | 3.20E-07 | 0.08 |  |  | rs3764400 | Body mass index | |
| TNFSF4 | 3.34E-07 | 0.06 |  |  | rs2205960 | Systemic lupus erythematosus | |
| LPL | 3.36E-07 | -0.10 |  |  | rs2083637 | Waist circumference and related phenotypes | |
| IRF4 | 3.77E-07 | 0.06 |  |  | rs1033180 | Celiac disease | |
| NR | 4.30E-07 | 0.05 |  |  | rs8014204 | Caffeine consumption | |
| WDR92 | 4.33E-07 | 0.13 |  |  | rs4078978 | Immune reponse to smallpox (secreted IFN-alpha) | |
| Intergenic | 4.52E-07 | 0.06 |  |  | rs3115573 | Nephropathy | |
| SYNRG | 6.21E-07 | -0.10 |  |  | rs2074409 | Response to angiotensin II receptor blocker therapy | |
| PARK16, SLC45A3, NUCKS1, RAB7L1, SLC41A1, PM20D1 | 6.32E-07 | -0.21 |  |  | rs947211 | Parkinson's disease | |
| C2,CFB | 6.63E-07 | -0.07 |  |  | rs429608 | Age-related macular degeneration | |
| PUS10 | 6.83E-07 | -0.07 |  |  | rs10188217 | Crohn's disease and celiac disease | |
| METTL1, CYP27B1 | 6.97E-07 | 0.12 |  |  | rs703842 | Multiple sclerosis | |
| NR | 7.43E-07 | 0.07 |  |  | rs1927702 | Body mass index | |
| CYP1A1, CYP1A2 | 8.82E-07 | 0.05 |  |  | rs2472297 | Coffee consumption | |

^1^Slope is given in absolute numbers

^2^Listed are all *cis*-rSNPs associated to a protein coding gene that are also found or are in LD to a SNP in the GWAS catalog

^3^The trait is taken from the GWAS catalog.
