# Supplementary material for: Single Nucleotide Polymorphisms with Cis-Regulatory Effects on Long Non-Coding Transcripts in Human Primary Monocytes
Source: PLoS One. 2014 Jul 15;9(7):e102612. doi: 10.1371/journal.pone.0102612 (PMC4099216; doi:10.1371/journal.pone.0102612)
Supplement: Methods S1 — Quadratic normalization. Detailed explanation of the quadratic normalization performed on the intensity levels obtained from the genotyping. (DOCX) [file pone.0102612.s007.docx]

**Methods S1: Quadratic normalization**

Detailed description of the quadratic normalization of the fluorophore signal intensity levels.

1. Calculate x: the log10 of the total signal from channel X and Y on genomic DNA.
   x = log10(Y.raw.signal.gDNA + X.raw.signal.gDNA)
2. Calculate y: the allele fraction of the genomic DNA
   y = Y.raw.signal.gDNA / (Y.raw.signal.gDNA + X.raw.signal.gDNA)
3. x is binned in bins of size 0.15 and the median of x and y are calculated per bin. This is then used in a regression:
   y.median ~ 1 + x.median + x.median^2^
4. From the regression we predict the allele fraction of genomic DNA and cDNA on the log10 of the total intensities:
   y.predicted.gDNA on log10(Y.raw.signal.gDNA + X.raw.signal.gDNA)
   and
   y.predicted.cDNA on log10(Y.raw.signal.cDNA + X.raw.signal.cDNA)
5. The quadratic normalised allele fractions for genomic DNA and cDNA can now be calculated:
   ((Y.raw.signal.gDNA / (Y.raw.signal.gDNA + X.raw.signal.gDNA)) - y.predicted.gDNA) + 0.5
   and
   ((Y.raw.signal.cDNA / (Y.raw.signal.cDNA + X.raw.signal.cDNA)) - y.predicted.cDNA) + 0.5
6. The difference between the normalised allele fractions is then used to calculate the ASE value.
